# Oil droplet fouling and differential toxicokinetics of polycyclic aromatic hydrocarbons in embryos of Atlantic haddock and cod

**DOI:** 10.1371/journal.pone.0180048

**Published:** 2017-07-05

**Authors:** Lisbet Sørensen, Elin Sørhus, Trond Nordtug, John P. Incardona, Tiffany L. Linbo, Laura Giovanetti, Ørjan Karlsen, Sonnich Meier

**Affiliations:** 1 Institute of Marine Research, Bergen, Norway; 2 Department of Chemistry, University of Bergen, Bergen, Norway; 3 Environmental Technology, SINTEF Ocean, Trondheim, Norway; 4 National Oceanic and Atmospheric Administration, Northwest Fisheries Science Center, Seattle, Washington, United States of America; 5 Department of Environmental Science, University of Siena, Siena, Italy; University of Siena, ITALY

## Abstract

The impact of crude oil pollution on early life stages (ELS) of fish, including larvae and embryos, has received considerable attention in recent years. Of the organic components present in crude oil, polycyclic aromatic hydrocarbons (PAHs) are considered the main class of compounds responsible for toxic effects in marine organisms. Although evidence suggests that they are more toxic, alkylated PAHs remain much less studied than their unsubstituted congeners. Recently, it was established that embryos of Atlantic haddock (*Melanogrammus aeglefinus*) are particularly sensitive to dispersed crude oil, and it was hypothesized that this was caused by direct interaction with crude oil droplets, which adhered to the chorion of exposed embryos. Such a phenomenon would increase the potential for uptake of less water-soluble compounds, including alkylated PAHs. In the current study, we compared the uptake of parent and alkylated PAHs in Atlantic cod (*Gadus morhua*) and haddock embryos exposed to dispersed crude oil at a range of environmentally relevant concentrations (10–600 μg oil/liter seawater). Although the species are biologically very similar, the cod chorion does not become fouled with oil droplets, even when the two species are exposed to dispersions of crude oil droplets under similar conditions. A close correlation between the degree of fouling and toxicological response (heart defects, craniofacial malformation) was observed. Oil droplet fouling in haddock led to both quantitative and qualitative differences in PAH uptake. Finally, kinetic data on a large suite of PAHs showed differential elimination, suggesting differential metabolism of unsubstituted versus alkylated compounds.

## Introduction

The impact of crude oil on marine fish and fisheries has received much attention in recent years, particularly in relation to major spill events such as the 1989 *Exxon Valdez* (EV) spill in the Prince William Sound, Alaska and the 2010 *Deepwater Horizon* (DWH) event in the northern Gulf of Mexico. Due to their high sensitivity to pollution, early life stages (ELS) of fish, including embryo and larval stages, have been studied extensively. The main sub-lethal, toxic responses in ELS of fish include cardiotoxicity and morphogenetic defects [1–3], but the toxicological mechanisms are still not fully understood. Lasting cardiac defects in juvenile fish could underlie population-level impacts years after a spill event [4]. As offshore oil exploration moves north into the Arctic, it occurs in sensitive spawning areas for several commercially important species of marine fish, such as in the Lofoten-Vesterålen area off the Norwegian coast[5, 6]. Consequently, a large effort towards developing risk assessment tools for evaluating the potential impact of oil exploration in these sensitive areas has been made [5–7]. However, to develop robust models for the effects of spilled crude oil on ELS of cold water marine fish, there is a need for more experimental data on bioaccumulation and critical body burdens of toxic oil compounds [8]. Likewise, there is a lack of data on how dispersed crude oil droplets affect fish ELS [8]. Along with dissolution of the water-soluble fraction (WSF), formation of dispersed oil droplets is considered one of the most important processes influencing the fate of spilled crude oil. Crude oil may be dispersed in the water column by turbulent wave action and/or application of chemical dispersants in the event of a surface spill [9, 10] or through high pressure jets during deep water oil and gas blowouts [11]. Formation of micron-sized oil droplets may increase the bioavailability of toxic oil constituents to marine organisms [9].

Polycyclic aromatic hydrocarbons (PAHs) constitute 0.1–1% of most crude oils. 2–3 ring PAHs (e.g. naphthalenes and phenanthrenes) are usually most abundant in fresh oils, while 4–6 ring PAHs become more dominant as the oil weathers. Alkyl substituted PAHs typically comprise > 90% of the total PAH content in petrogenic oils [12, 13]. Previously, it was believed that only water soluble oil constituents, mainly the PAHs, were the responsible for crude oil toxicity in marine systems [14–17], but new observations suggests that the presence of crude oil droplets in the exposure system leads to more severe effects than if only the water accommodated fraction (WAF) is present [18]. Recently, it was established that the Atlantic haddock (*Melanogrammus aeglefinus*) was particularly sensitive to dispersed crude oil, and it was hypothesized that this was caused by direct interaction with crude oil droplets, which adhered to the chorion of the exposed embryos [3, 19, 20]. Such behavior potentially creates a second pathway for uptake of crude oil derived compounds into the embryos, in particular increasing the potential for uptake of less water-soluble compounds, such as heavier and more alkylated PAHs. Although limited data suggests that alkylated PAHs are more toxic, these compounds remain much less studied than their unsubstituted ‘parent’ congeners [21, 22], which are readily available from commercial sources.

Despite the vast number of PAH toxicity studies conducted, a limited number of these studies report PAH body burden concentrations in fish ELS [23–28]. This is due, in part, to the need for large samples masses (1–10 g wet weight) to achieve the instrumental detection limits [29, 30], particularly for samples that contain low PAH concentrations. Recently, methodologies were developed for the analysis of trace amounts of both parent and a wide range of alkylated PAHs in small (< 0.1 g) tissue samples [31, 32]. These methods enable the determination of total PAH body burden in fish egg samples from low dose exposure experiments using as little as 100 individuals or less per sample.

Atlantic haddock and cod are two of the most commercially important fish species in Norway, representing the largest Norwegian fisheries both in landing and in value. Both species have their main spawning grounds in the Lofoten-Vesterålen area [33]. The two species are biologically very similar, both in terms of egg lipid content (0.6–0.7%) [32], egg size (1.3–1.5 mm diameter) [34] and developmental biology [35–37]. Although individual studies of crude oil effects on ELS of Atlantic haddock embryos and larvae and cod larvae have previously been published [3, 17, 19, 20, 38], there has yet to be a direct comparison of the toxicological response in the two species. In the current study, the aim was to compare the uptake of parent and alkylated PAHs in cod and haddock embryos exposed to mechanically dispersed Norwegian sea crude oil at a range of environmentally relevant concentrations (10–600 μg oil/L seawater). The body burden of parent and alkylated PAHs in the embryos was measured at intervals during constant exposure starting at 1 day post fertilization (dpf) lasting until hatching of the larvae at 11 (haddock) or 12 (cod) dpf, when the exposure was stopped and the decline in body burden was followed until 2 days post hatching (dph). Despite their similarities, the cod chorion did not become fouled with oil droplets like the haddock even when the two species are exposed to dispersions of crude oil droplets under the same conditions. A close correlation between the occurrence and degree of oil fouling and morphological and heart malformations was observed. This was explained by increased internal uptake of 3–6 ring PAHs, in particular alkylated PAHs.

## Materials and methods

### Animal husbandry and oil exposure regime

Fertilized Atlantic cod and haddock eggs were collected from brood stocks kept at the Institute of Marine Research (IMR), Austevoll Research station, transferred to indoor egg incubators, and maintained at 7±1°C until transfer to exposure tanks. At 1 day post fertilization (dpf), the eggs were transferred into circular exposure tanks (50 L) of green poly ethylene plastic (approximately 12,000 eggs in each tank). The flow of sea water through the tanks was 32 L/hr, the water temperature maintained at 7±1°C. The light regime for the exposure tanks was 12 hours light:12 hours dark provided by broad spectrum 2x36W Osram Biolux 965 (Munich, Germany, http://www.osram.com) dimmable fluorescent light tubes with 30 min smooth transitions between light and dark.

The crude oil used in the exposure was supplied by SINTEF Materials and Chemistry (Trondheim, Norway), and was a crude oil blend from the Heidrun oil field of the Norwegian Sea. Prior to use, the oil was artificially weathered using a well-established evaporation procedure where evaporation of the lighter components from the fresh oil was achieved as a one-step distillation to the vapor temperature of 200°C. This results in a residue with an evaporative loss corresponding to approximately 0.5–1 day of weathering on the sea surface (water temperature of around 10°C) [39]. The principle of the oil exposure system is detailed elsewhere [40], and oil exposure was performed as described previously [3]. Briefly, crude oil dispersions were made by mixing clean seawater with crude oil using an oil droplet generator. A mechanical valve system allowed systematic dilution of the stock dispersion to nominal oil doses of 10–600 μg/L. In the current study, the results of three separate experiments (performed in the same way) were included. Un-exposed control groups were included for both species in all experiments. The first and second experiment included exposure doses spanning the entire nominal oil dose range for cod and haddock, respectively. Due to high mortality observed at the highest dose for haddock, the third experiment included four low-intermediate doses for haddock embryos (30–300 μg/L nominally). To study the effect of the oil fouling on the haddock egg chorion an exposure with filtered dispersion (referred to as the water-soluble fraction, WSF) of the second highest exposure dose (300 μg/L) was included in the second haddock experiment. To create the WSF, the 300 μg/L dispersion was continuously filtered through a custom-made filter containing fine glass wool on top of a Whatman GF/F glass microfiber filter (Whatman Ltd., Maidston, UK) with nominal particle retention of 0.7 μm. The filter was replaced every 24 hours to prevent clogging. The exposure conditions were otherwise identical to the oil droplet exposures. All exposure experiments started at 1 dpf and were stopped when 50% hatching of embryos was observed, this happened at 12 dpf (11 days of exposure) for cod and 11 dpf (10 days of exposure) for haddock. The hatched larvae remained in clean water until 2 days post hatching (dph). A schematic overview of the experiment can be found in S1 Fig.

### Analytical chemistry

#### Chemical and materials

Certified standard solutions (100–1000 μg/mL) of *n*-alkanes (C14-C32, only even + pristane/phytane), PAHs, alkylated PAHs, heteroaromatics and deuterated PAHs were purchased from Chiron AS (Trondheim, Norway). Spike and calibration standards were prepared by dilution in *n*-hexane or, for body burden analysis, in a matrix extract of unexposed haddock and cod eggs [31]. Details on all analytes are compiled in S1 Table. The deuterated internal standard used as a surrogate spike contained naphthalene-*d*8, biphenyl-*d*8, acenaphthylene-*d*8, anthracene-*d*10, pyrene-*d*10, perylene-*d*12 and indeno[1,2,3-*cd*]pyrene-*d*12. Dichloromethane (DCM) and *n*-hexane were of GC Suprasolv® analytical grade and supplied by Merck (Oslo, Norway).

#### Analysis of cod and haddock eggs

Body burden samples (pooled ~100 mg eggs or exactly 100 individual larvae) were collected at selected time-points during exposure (12 hours, 1, 2, 3, 5, 7, 9 days) and after exposure end (0, 1, 2 days). Triplicate samples were taken from each exposure tank, including controls. Pools of crude oil exposed eggs were quickly rinsed in clean seawater with the purpose to eliminate free oil droplets from the sample. All eggs were examined under the microscope, and any dead eggs were removed from the sample. At day 10 (during hatching), 100 individual unhatched haddock eggs from the highest exposure dose were sampled. The chorions and embryos of these eggs were manually separated using fine forceps and each matrix was analysed separately. The samples were preserved by flash-freezing in liquid nitrogen and stored in poly ethylene cryovials at -80°C until further handling. Extraction was performed as described in Sørensen et al [32]. After transfer to glass vials and addition of surrogate standards (100 ng/g sample), the samples were homogenized in *n*-hexane-dichloromethane (1:1 v/v, 2 mL) using a microprocessor (Virtis Tempest IQ 2.0), followed by addition of sodium sulphate (150 mg), vortex extraction (30 s) and centrifugation (2000 rpm, 2 min). The supernatant was collected and the extraction step was repeated two additional times. The combined organic extract was concentrated to ~1 mL prior to clean-up by solid phase extraction (SPE) using silica (Agilent Bond Elut SI, 500 mg, Agilent Technologies, USA). The extract was eluted with dichloromethane in *n*-hexane (1:9, v/v, 6 mL). Immediately prior to the analysis, the volume of the cleaned extract was reduced to 100 μL under a gentle stream of N_2_. Laboratory blank samples (empty vials) were included in the extraction daily. Background levels of PAHs identified in laboratory blanks were subtracted from the samples. Quality assurance samples comprised of unexposed cod or haddock eggs (150 mg) spiked with a mixture of PAHs (1 ng of each) were included weekly and demonstrated the procedure repeatability over time (% RSD < 25). Recovery of surrogate internal standards were in the range 40–110%.

#### Water samples

Water samples (1 L) were taken from each exposure tank at the beginning, during and at the end of each experiment (total three samples). The samples were preserved by acidification (H_2_SO_4_, pH<2), addition of dichloromethane (30 mL) and stored at4°C in the dark until further processing (no samples were stored longer than one week). The samples were extracted twice by partitioning to dichloromethane (2x30 mL) in a separatory funnel (2 min). Deuterated internal standards were added immediately prior to extraction to account for analyte loss during extraction. Laboratory blank samples (deionized water) were included for each batch extraction, and no significant level of background contamination was observed. Recovery of surrogate internal standards were in the range 60–100%. Water concentrations for individual exposures are reported as the mean of the three samples.

#### PAH, alkyl PAH and alkane analysis

An Agilent 7890 gas chromatograph coupled with an Agilent 7010 triple quadrupole mass spectrometer fitted with an EI source and collision cell was used (Agilent Technologies, Santa Clara, CA, USA). Two Agilent J&W DB-5MS UI GC-columns (15 m × 0.25 mm x 0.25 μm) were coupled in series through a purged ultimate union (PUU). The carrier gas was helium at constant flow (1.2 mL/min). Samples (1μL) were injected at 280°C splitless. The oven temperature was held at 60°C for 1 min, then ramped to 120°C by 40°C/min and finally ramped to 310°C by 5°C/min. The temperature was held at 310°C for 5 minutes while the first column was back-flushed. The transfer line temperature was 280°C, the ion source temperature was 230°C and the quadrupole temperatures were 150°C. The EI source was operated at 70 eV. N_2_ was used as collision gas at a flow of 1.5 mL/min and helium was used as a quench gas at a flow of 4 mL/min. Target PAH analytes were identified by two unique multiple reaction monitoring (MRM) transitions and quantified by the most intense peak [32]. Alkyl PAH clusters were determined by MRM using transitions from the molecular ion, as described previously in Sørensen et al [31]. Calibration tandards were run daily to monitor system performance and a variation of no more than 25% was accepted. Method performance characteristics are reported in Sørensen et al [31, 32]. Alkanes were measured by GC-MS/MS using MRM transitions 99^+^ → 57^+^ and 127^+^ → 85^+^. The analytical system and conditions were otherwise as described above, except that an Agilent 7000C mass spectrometer was used and the quench gas flow was 2.25 mL/min. The rate of oil fouling on haddock eggs over the course of the exposure was estimated by the rate of increase of the relative abundance of the Σ(*n*C19-*n*C32) to internal standard pyrene-*d*12.

### Imaging of live embryos/larvae and measurements of cardiac function

Digital still micrographs of live larvae were obtained with an Olympus SZX-10 stereo microscope equipped with a 5Mp resolution camera (Infinity 2–5c, from Lumenera) while video recordings where obtained using the same microscope and a Nikon SMZ-800, both with 1.2Mp resolution video cameras (Unibrain Fire-I 785c). Image magnification was calibrated with a stage micrometer. BTV Pro 5.4.1 (www.bensoftware.com) was used to control the video camera. Video microscopy was performed at 2 and 3 dph for cod and haddock embryos, respectively. Animals were immobilized in a glass petri dish filled with 3% methylcellulose and kept at 8°C using a temperature controlled microscope stage. Length of larvae, ethmoid plate and area of oedema were measured using ImageJ [41] with the ObjectJ plugin (https://sils.fnwi.uva.nl/bcb/objectj/index.html). ImageJ was also used to measure the ventricular and atrial diastolic (D) and systolic diameter (S) to estimate the fractional shortening (FS = (D−S)/D). Measurements from both images and videos were performed blindly without information of the exposure.

### RNA collection and preparation

Embryos (before hatching) were collected at 10 and 11 dpf for haddock and cod, respectively. All animals collected for RNA extraction were imaged under a microscope before they were frozen in liquid nitrogen and stored at −80°C. Two pools of embryos and larvae from each tank were collected at all sampling times for total RNA extraction. Total RNA was isolated from frozen pools of embryos using Trizol reagent (Invitrogen, Carlsbad, California, USA), according to procedures provided by the manufacturer. All samples were homogenized in their respective lysis buffer 2×20 seconds at 5000 rpm using a Precellys 24 apparatus. The amount of RNA was quantified using a Nanodrop spectrophotometer (NanoDrop Technologies, Wilmington, DE, USA), and quality checked using a 2100 Bioanalyzer (Agilent Technologies, Santa Clara, CA). cDNA was subsequently generated using SuperScript VILO cDNA Synthesis Kit (Life Technologies Corporation), according to the manufacturer’s instructions. The cDNA was normalized to obtain a concentration of 50ng/μL.

### Real time qPCR

Specific primers and probes for real-time, qPCR analysis of Atlantic haddock and cod cytochrome P4501A (*cyp1a*) and the technical reference *ef1a* (elongation factor 1 alpha, housekeeping gene) were designed with Primer Express software (Applied Biosystems, Carlsbad, California, USA) according to the manufacturer’s guidelines. Primer and probe sequences are given in S2 Table. TaqMan PCR assays were performed in duplicate, using 96-well optical plates on an ABI Prism Fast 7900HT Sequence Detection System (Applied Biosystems, Carlsbad, CA, USA) with settings as follows: 50°C for 2min, 95°C for 20 s, followed by 40 cycles of 95°C for 1 s and 60°C for 20 s. Duplicates with standard deviation2 (SD2) ≤ 0.05 were either rerun or eliminated from the dataset. No template, no reverse transcriptase enzyme control and genomic DNA controls were included. For each 10μl PCR reaction, a 2 μl cDNA 1:40 dilution (2.5 ng) was mixed with 200 nM fluorogenic probe, 900 nM sense primer, 900 nM antisense primer in 1xTaqMan Fast Advanced Master Mix (Applied Biosystems, Carlsbad, California, USA). Gene expression data for *cyp1a* was calculated relative to the control samples after normalization to the reference gene (*ef1a*) using the ΔΔCt method as described in detail in Bogerd et al. [42].

### Statistics

Statistical analysis was performed with GraphPad Prism, version 6 (GraphPad Software Inc., 1996, La Jolla, California, USA). Significant differences in structural and functional measurements were tested with one-way ANOVA using the Tukey-Kramer multiple comparison. The level of significance was set at p < 0.05 unless otherwise stated. Principal component analysis (PCA) was performed using singular value decomposition in R software [43]. Prior to statistical treatment, individual PAH or cluster alkyl PAH concentrations (pg/embryo) were normalized to total PAH concentration in the sample, scaled to the mean of each variable and centered. After initial assessment of the data, outliers, in form of samples or variables where the primary variance was caused by analytical variation due to low concentrations, were removed from the data set. Fitting of data was performed using Excel (linear functions) or R (polynomial functions).

### Ethics statement

All animal experiments within the study were approved by NARA, the governmental Norwegian Animal Research Authority (http://www.fdu.no/fdu/, reference number 2012/275334-2). All methods were performed in accordance with approved guidelines. No humane endpoints were used during the experiment because the potential endpoint criteria due to their small size had to be evaluated under a microscope when they were sampled. The animals were monitored every day, and any dead larvae were removed. All embryos and larvae sampled were frozen in liquid nitrogen. The Austevoll Research station has the following permission for catch and maintenance of Atlantic haddock: H-AV 77, H-AV 78 and H-AV 79. These are permits given by the Norwegian Directorate of Fisheries. Furthermore, the Austevoll Research station has a permit to run as a Research Animal facility using fish (all developmental stages), with code 93 from the national Institutional Animal Care and Use Committee (IACUC); NARA.

## Results

### Characterization of the exposure media

Parent and alkyl PAH concentrations in the studied crude oil and exposure groups are summarized in S3 Table. For the range of applied nominal crude oil concentrations (10–600 μg oil/L seawater), the measured concentrations of total PAHs (tPAHs), including alkylated C1-C4 benzothiophenes, naphthalenes, fluorenes, dibenzothiophenes, phenanthrenes, chrysenes and pyrenes measured as clusters, in water samples were 0.1–9 μg/L. Since the focus of this paper is on PAHs, the exposure groups are identified by their measured tPAH concentration. The PAH profile in the water dispersions were similar to the crude oil profile, with naphthalenes comprising more than half of the measured PAHs, followed by a significant fraction of 3-ring PAHs (particularly phenanthrenes and dibenzothiophenes). The 4–6 ring PAHs, including alkylated 4-ring compounds (e.g. C1-chrysenes) were present in quantifiable amounts in the droplet dispersions (> 5% of tPAH), but mostly depleted (< 1% of tPAH) in the filtered solutions (water-soluble fraction, WSF), indicating the absence of oil droplets.

### Oil droplet fouling on the haddock egg chorion

**Fig 1:**
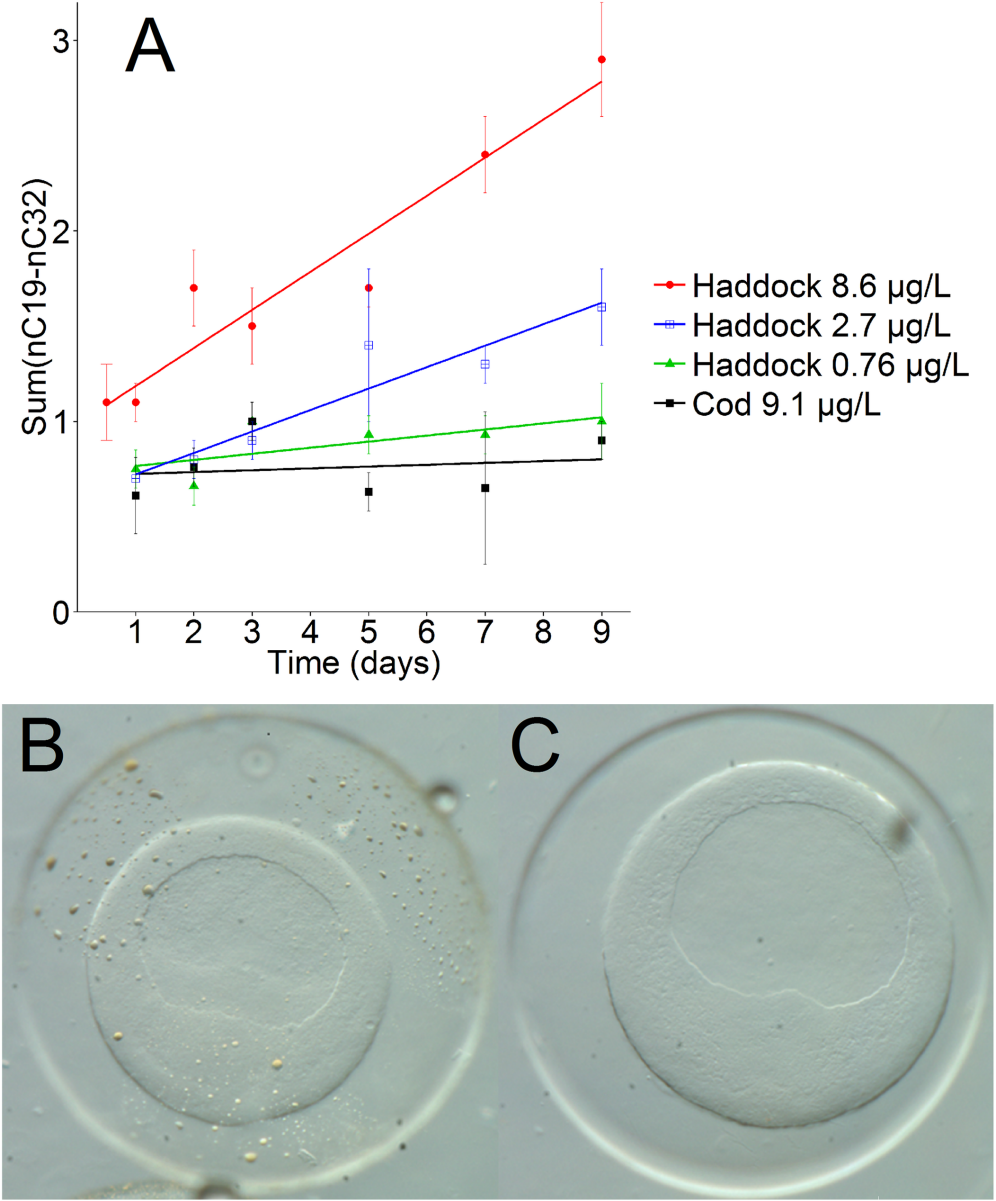
Haddock (B) and cod (C) embryos exposed to 600 μg/L crude oil dispersions for 12 hours, where fouling of oil droplets on the chorion of the haddock egg can be observed. In panel A the relative response of alkanes (Σ(nC19-nC32)) normalized to the response of internal standard pyrene-*d*12, during the uptake period for three doses of crude oil exposed haddock and the highest exposure dose for cod. Error bars represent one standard deviation (*n* = 3). A linear trendline is fitted to each group (*R*^*2*^ = 0.926, 0.921, 0.530 and 0.023 for haddock 8.6, 2.7, 0.76 and cod 9.1, respectively).

Droplet fouling on the chorion of haddock eggs was observed microscopically with exposure to the higher doses of dispersed crude oil (3–9 μg/L tPAH). Identically treated cod eggs showed no fouling, represented for the highest dose in Fig 1. To demonstrate this by a more sensitive and quantitative method than microscopy, we used chemical analysis of large *n*-alkanes (Σ(*n*C19-*n*C32)) in the extracted egg (not dechorionated) samples. These compounds are used as markers of whole oil due to their high abundance in oil and extremely poor water solubility. Concentrations of *n*-alkanes associated with haddock eggs increased linearly with time (Fig 1A) for the highest exposure doses (0.7–9 μg/L tPAH). This shows that the haddock egg extracts contain both internally accumulated and externally adhered oil compounds. For the lowest doses of haddock, all cod samples, and haddock eggs exposed to only water-soluble fraction (WSF) of oil, the alkane pattern could not be distinguished from the background in the control samples (S4 Table).

### Higher frequencies of toxicity phenotypes in haddock vs. cod

**Fig 2:**
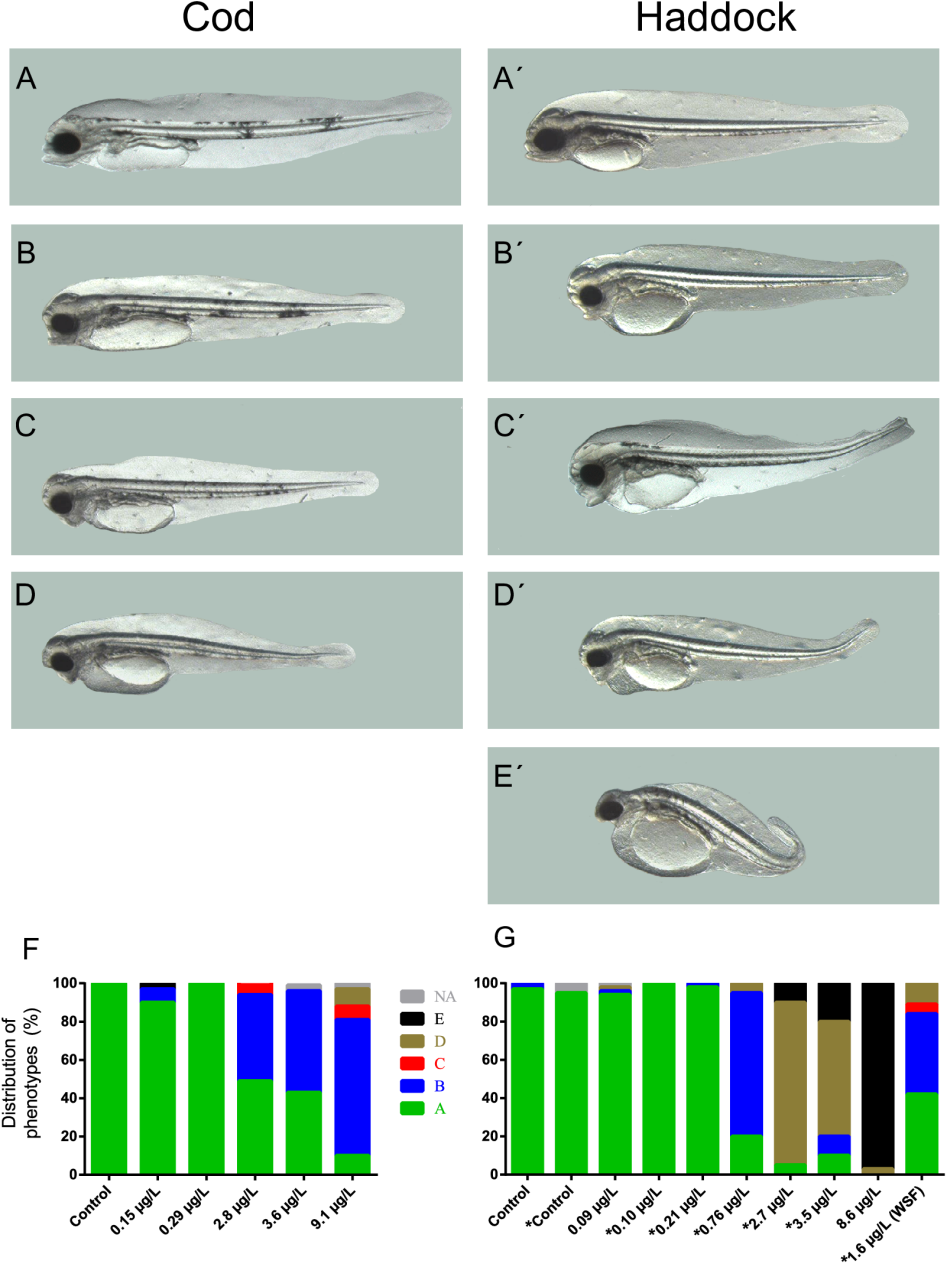
Craniofacial phenotypes in cod (A-D) and haddock (A’-E’) 3 days after hatching. (A,A’) Control and four craniofacial abnormality phenotypes: Larvae with (B, B’) underdeveloped upper jaw, (C, C’), with an underdeveloped upper jaw and posteriorly displaced lower jaw, (D, D’) underdeveloped upper jaw and hanging lower jaw, and (E’) overall extremely reduced jaw structures. (F) and (G) show the distribution of craniofacial phenotypes in increasing concentrations of dispersed crude oil and water soluble fraction in cod and haddock, respectively. *: haddock experiment 2.

We quantified several toxicity phenotypes (cardiac and craniofacial defects) in both haddock and cod. In addition, as a measure of effective exposure doses, we quantified expression of *cyp1a* mRNA, an extremely sensitive biomarker for the presence of PAHs in tissues [44]. At the highest exposure dose (8.6 μg/L tPAH), very few haddock embryos survived through hatching (after 10 days of exposure). Surviving larvae (observed at 2 dph) had severe deformities, such as craniofacial malformations, spinal curvature and significantly shorter body length, and combined yolk sac and heart oedema. Severe cardiotoxic responses were also observed (S5 Table). An overview of the distribution of observed phenotypes is found in Fig 2. A detailed explanation of the applied phenotype characterization and measurement of other malformations can be found in Sørhus et al. [19]. Hatched cod larvae from the intermediate to high exposure doses (2.8, 3.6 and 9.1 μg/L tPAH) showed malformations and defects of increasing severity, but at significantly lower frequencies than observed in haddock exposed to equivalent doses (2.7, 3.5 and 8.6 μg/L tPAH). Effects observed in haddock at a lower dose (0.76 μg/L tPAH) were also more severe than observed in any of the medium cod exposure groups. Defects in haddock exposed to only the WSF (1.6 μg/L tPAH) were at the same frequency as observed in cod exposed at the same nominal dispersed oil concentration (~3 μg/L tPAH). Few to none effects were observed in either cod or haddock embryos exposed to crude oil dispersions with tPAH < 0.3 μg/L.

**Fig 3:**
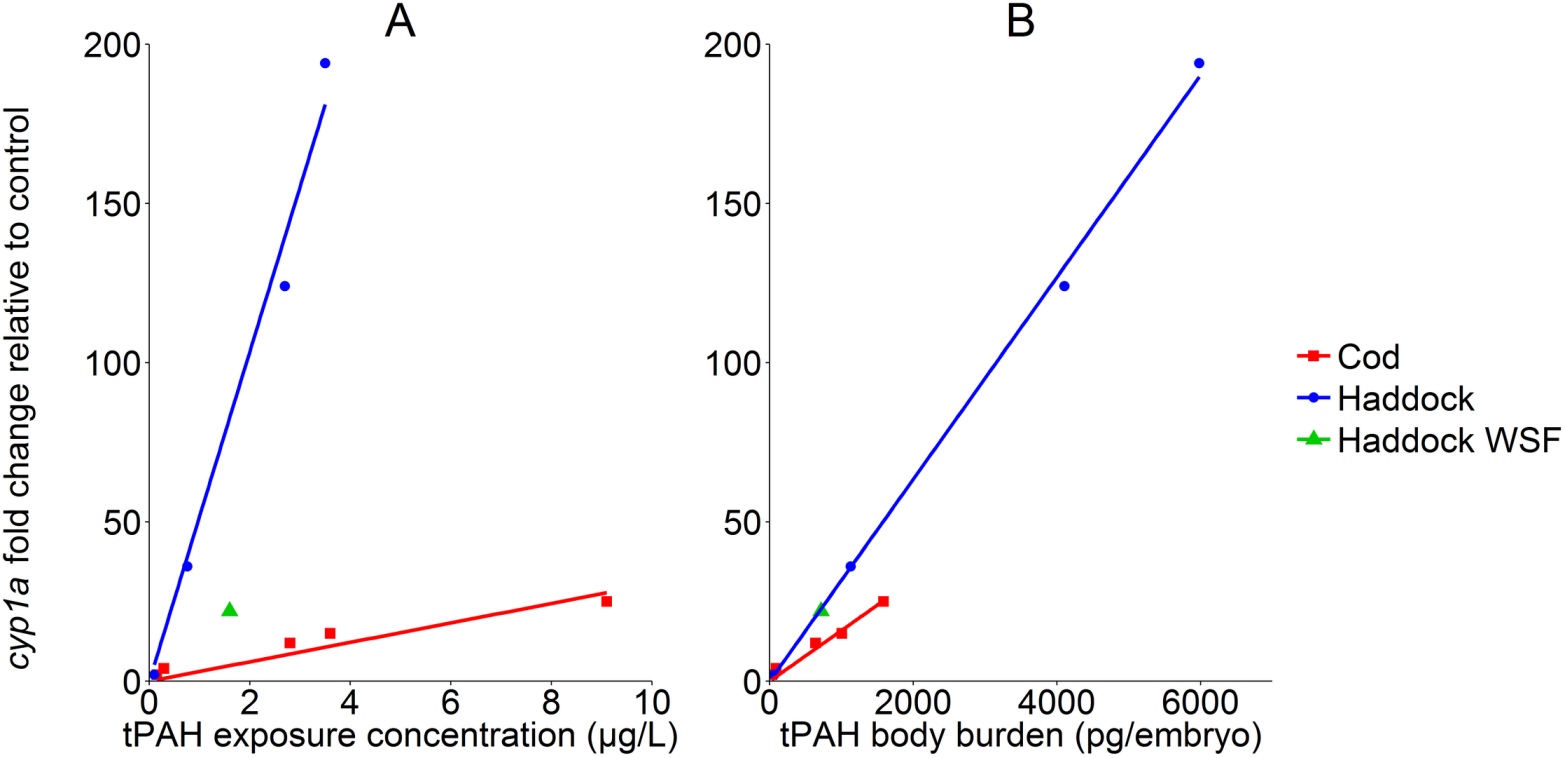
Expression of *cyp1a* relative to control measured two days prior to hatching (10 and 11 dpf for haddock and cod, respectively), expressed as a function of exposure concentration (A) and body burden concentration (B). A linear regression is fitted to each of the cod and haddock data sets (*R*^*2*^ = 0.983, 0.953, 0.998, 0.724 for haddock and cod cyp1a compared to water concentration, and for haddock and cod *cyp1a* compared to body burden concentration, respectively).

Induction of *cyp1a* was linearly related to both dispersed oil concentration (Fig 3A) and tissue dose (Fig 3B) for both haddock and cod. At any given dispersed oil concentration, *cyp1a* induction was orders of magnitude higher for haddock than cod at the equivalent concentration (Fig 3A), consistent with higher tPAH body burdens. Based on tissue dose, there was also a trend for much higher *cyp1a* induction in haddock relative to cod with the same tPAH body burden (Fig 3B), but a statistical difference could not be determined due to sampling design. The single concentration of WSF exposure in haddock led to *cyp1a* levels that were within same linear range based on tPAH body burden (Fig 3B). The higher levels of *cyp1a* induction at the same tPAH tissue dose for haddock relative to cod suggest that PAH composition in haddock was different.

### Oil droplets fouling on the haddock chorions leads to differential PAH uptake

**Fig 4:**
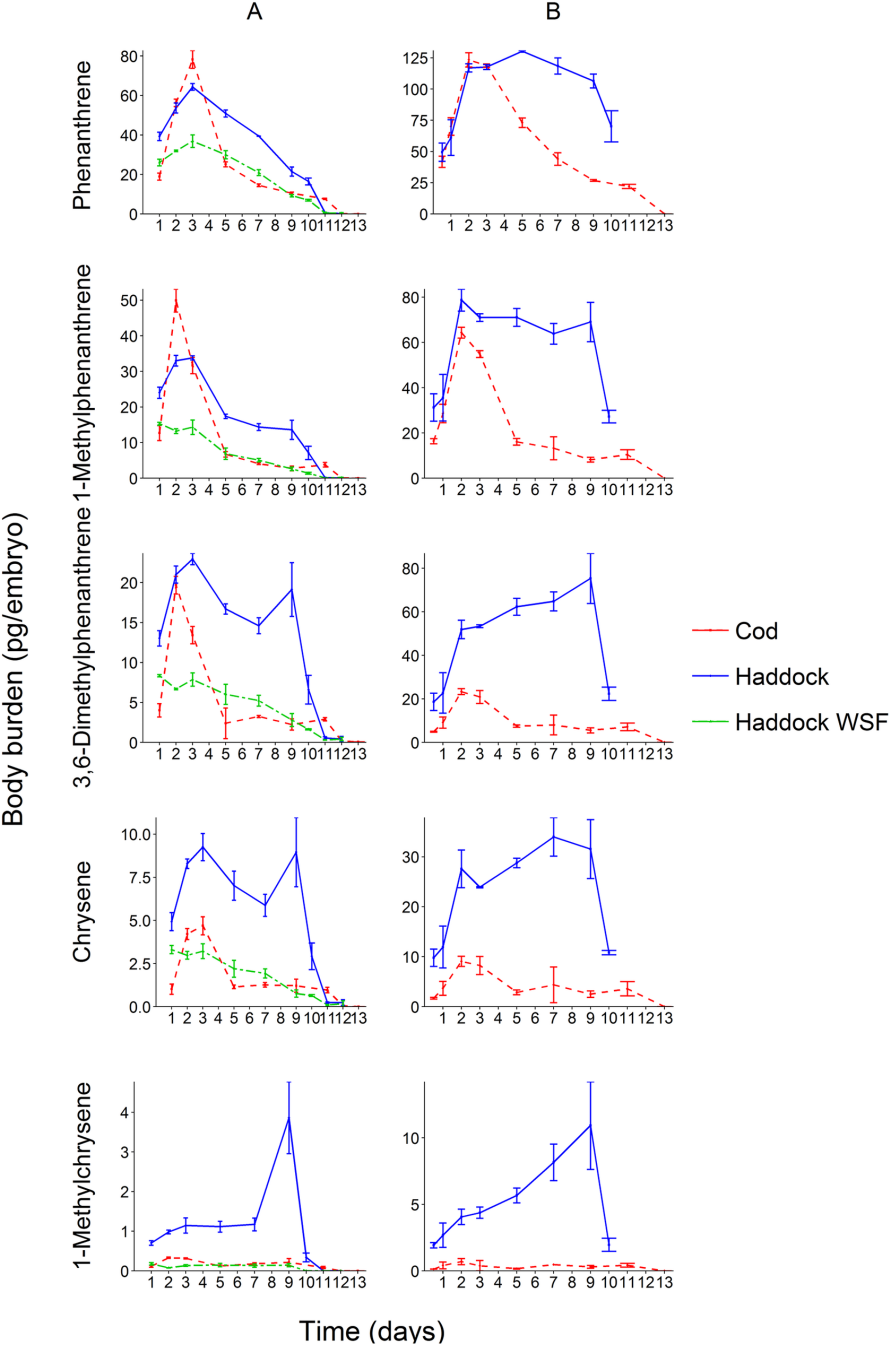
The change in body burden of phenanthrenes and chrysenes in crude oil exposed cod and haddock throughout the exposure period at two different exposure concentrations, ~3 μg/L tPAH (A) and ~9 μg/L tPAH (B). Haddock embryos exposed to only the water-soluble fraction (WSF) at ~3 μg/L tPAH is also included. Body burden in haddock embryos exposed at ~9 μg/L tPAH could not be measured beyond day 10 due to high mortality. Error bars indicate standard deviation (*n* = 3).

PAHs and alkyl PAHs (both individual compounds and clusters) were measured for embryos (exposure days 0–9) and hatched larvae throughout and after exposure end (Table 1, S6 Table). As expected from the observed oil droplet fouling, there were key differences in haddock compared to cod. At each exposure concentration, haddock embryos generally showed a plateau of associated tPAH by day 3 (Fig 4B and S2A and S2B Fig), while cod embryos showed a peak at day 3 followed by a decline (Fig 4, S2 and S5 Figs). Notably, after hatch, the tPAH levels for haddock showed a marked decline (S2A and S2B Fig) due to the loss of the oil-droplet bearing chorion. In contrast to embryos exposed to dispersed oil droplets, haddock embryos exposed to the WSF showed an uptake pattern similar to cod, with a peak at day 3 followed by a decline (Fig 4B and S2B Fig). A similar pattern was observed for individual analytes, for example, parent and methylated phenanthrenes and chrysenes (Fig 4). Most individual PAHs followed the same pattern (S3–S5 Figs). Body burden in haddock embryos exposed at ~9 μg/L tPAH could not be measured beyond day 10 due to high mortality and low hatching success.

**Table 1:** Total PAH body burden (including alkyl clusters) measured in cod and haddock eggs at day 3 and day 9 of exposure.

	C_W_ (μg/L)	Body burden (pg/embryo)						Body burden (ng/g wet weight)						Body burden (ng/g lipid)					
		Day 3			Day 9			Day 3			Day 9			Day 3			Day 9		
**Cod**	0.15 ± 0.01	101	±	33	32.4	±	7.6	22	±	10	7.7	±	1.2	3 143	±	1 414	1 100	±	171
	0.29 ± 0.03	147	±	15	91	±	11	36.3	±	2.5	22.7	±	2.1	5 186	±	357	3 243	±	297
	2.8 ± 0.5	1 902	±	88	639	±	98	542	±	16	159	±	12	77 429	±	2 314	22 714	±	1 722
	3.6 ± 0.6	1 560	±	41	1 006	±	193	438	±	29	258	±	15	62 571	±	4 143	36 857	±	2 143
	9.1 ± 1.4	4 455	±	558	1 585	±	153	1 101	±	64	412	±	41	157 286	±	9 087	58 857	±	5 891
**Haddock**	0.09 ± 0.11	239	±	330	78	±	37	18	±	21	5.7	±	2.1	2 571	±	3 000	814	±	300
	0.17 ± 0.06	161	±	96	78.5	±	3.8	25.3	±	3.2	15.7	±	1.5	3 614	±	457	2 243	±	214
	0.21 ± 0.03	238	±	56	153	±	41	43.7	±	4.0	28.3	±	5.5	6 243	±	571	4 043	±	786
	0.76 ± 0.09	1 050	±	46	1 128	±	209	151	±	13	99	±	14	21 571	±	1 857	14 143	±	2 000
	2.7 ± 0.5	3 280	±	108	4 106	±	482	594	±	15	351	±	30	84 857	±	2 143	50 143	±	4 286
	3.5 ± 0.8	4 959	±	199	5 983	±	991	883	±	64	439	±	29	126 143	±	9 143	62 714	±	4 143
	8.6 ± 1.4	8 790	±	68	13 315	±	2 532	1 080	±	54	1 194	±	111	154 286	±	7 714	170 571	±	15 857

**Fig 5:**
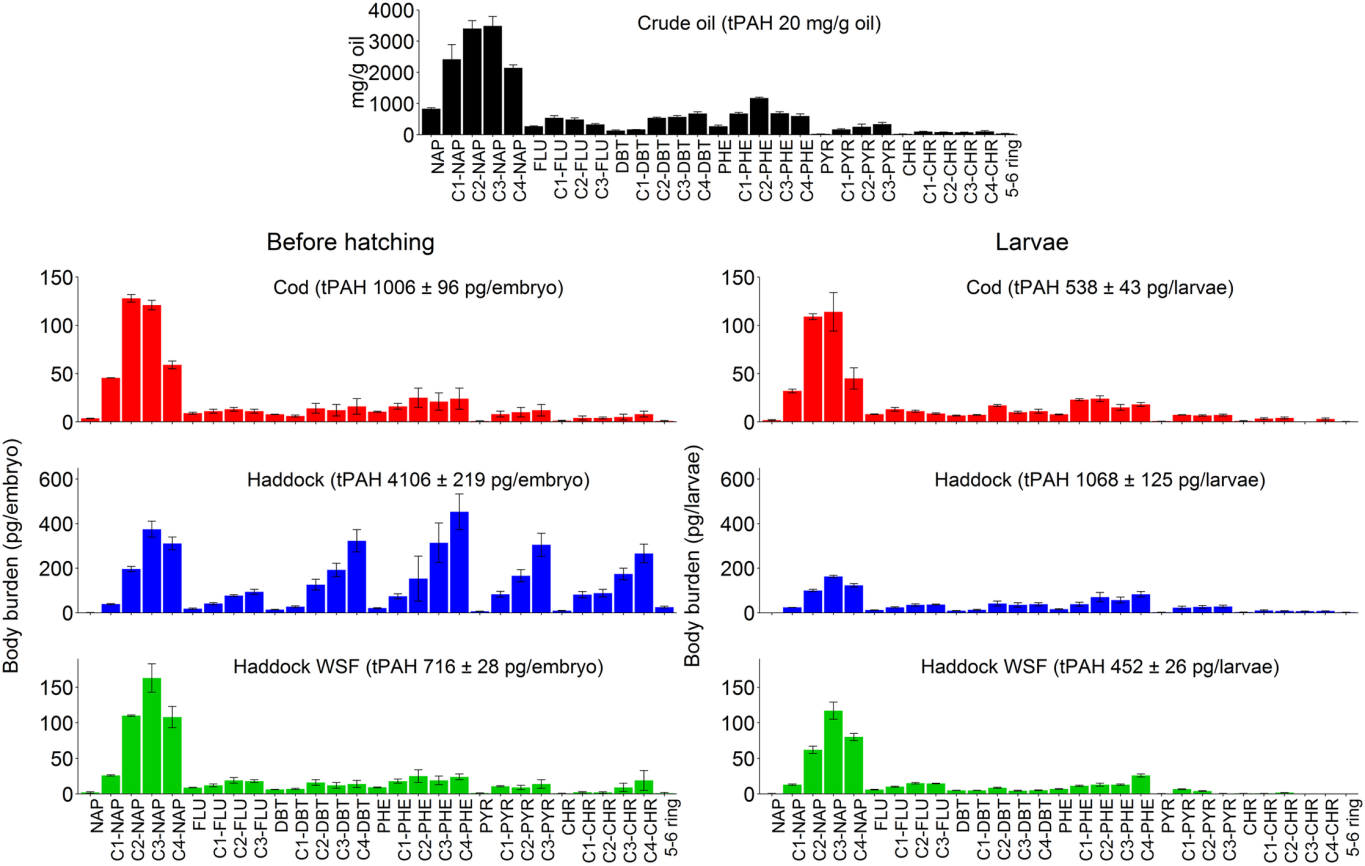
Body burden (pg/embryo or larvae) of PAHs and alkyl PAH clusters in eggs before hatching (left) and larvae (right) exposed to ~3 μg/L tPAH and WSF fraction. Distribution of PAHs (mg/g oil) in the crude oil is shown for comparison. For visibility, all 5–6 ring compounds are grouped. Error bars represent one standard deviation (*n* = 3). NAP = naphthalene, FLU = fluorene, DBT = dibenzothiophene, PYR = pyrene, CHR = chrysene.

The higher concentrations of embryo-associated PAHs in droplet-exposed haddock were expected in part due to whole oil on the chorion. To assess if body burden in the embryo proper was different, we compared PAH concentrations at day 10/11 in hatched larvae of cod and haddock exposed to similar concentrations of dispersed oil (cod 2.8 μg/L tPAH, haddock 2.7 μg/L), and haddock exposed to just the WSF of the same dose just before hatch at day 9 (Fig 5). Prior to hatch (at day 9), haddock exposed to droplets had a tPAH of 4106 ± 219 pg/embryo compared to cod with only 1006 ± 96 pg/embryo. Notably, haddock showed a distinctly different pattern of PAHs, dominated by higher degrees of alkylation for 3- and 4-ring compounds (Fig 5). Although all three treatments showed a reduction in tPAH after hatch, the drop was much larger for droplet-exposed haddock, consistent with loss of a large portion associated with the chorion. However, the internal concentrations of both intermediate and heavier compounds in newly hatched haddock larvae (day 10) were increased compared to both droplet-exposed cod and haddock exposed to only WSF.

**Fig 6:**
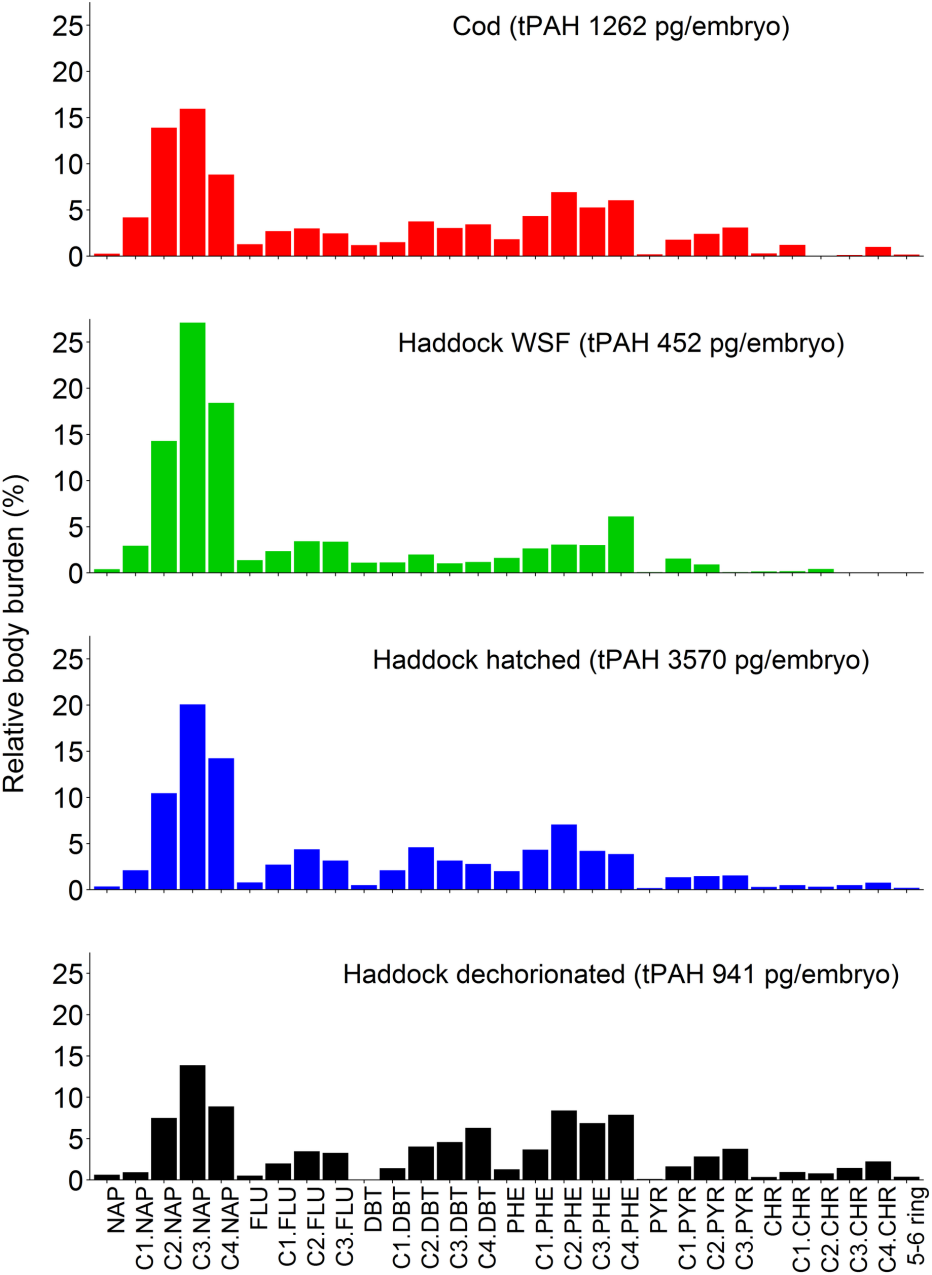
Relative body burden (%) of select 2–6 ring PAHs and alkyl PAHs in larvae of cod exposed to ~9 μg/L tPAH, haddock exposed to WSF and haddock exposed to ~9 μg/L tPAH who hatched in the tank and that was dechorionated. NAP = naphthalene, FLU = fluorene, DBT = dibenzothiophene, PYR = pyrene, CHR = chrysene.

We also attempted to quantify the relative amounts of chorion-associated PAH and true body burden of pre-hatch (day 9) haddock embryos by manually removing the chorions of embryos exposed to the highest dose (9 μg/L tPAH). Due to the difficulty of this process, the yolk mass was lost from some embryos, which was likely to lead to an underestimation of the actual body burden. Despite potential loss of yolk-associated PAHs, dechorionated embryos had a body burden of 984 pg/embryo, roughly the same as cod exposed to only 3 μg/L (S7 Table). Intriguingly, the loss of compounds in manually dechorionated embryos was not uniform across all PAH classes. We compared the percentage of compounds recovered on the chorions, manually extracted embryo and hatched larvae relative to the total in intact eggs (S10 Fig). On average, 50–70% of the heavier compounds (> 3-rings) were found on the chorion, while most of the smaller compounds were found in the larvae. We also compared the PAH compositions in hatched cod larvae exposed to droplets, hatched haddock exposed to WSF (at roughly the same tPAH body burden), and hatched haddock larvae and manually dechorionated embryos from the same droplet exposure (Fig 6). Importantly, the pattern of PAHs in cod exposed to droplets and haddock exposed to WSF were virtually indistinguishable and dominated by 2-ringed naphthalene compounds. In contrast, both hatched haddock larvae and manually-dechorionated embryos from the droplet exposure showed PAH patterns with higher percentages of 3-ringed compounds, and in particular the alkylated homologs.

### Differential elimination of alkyl-PAHs by both cod and haddock

**Fig 7:**
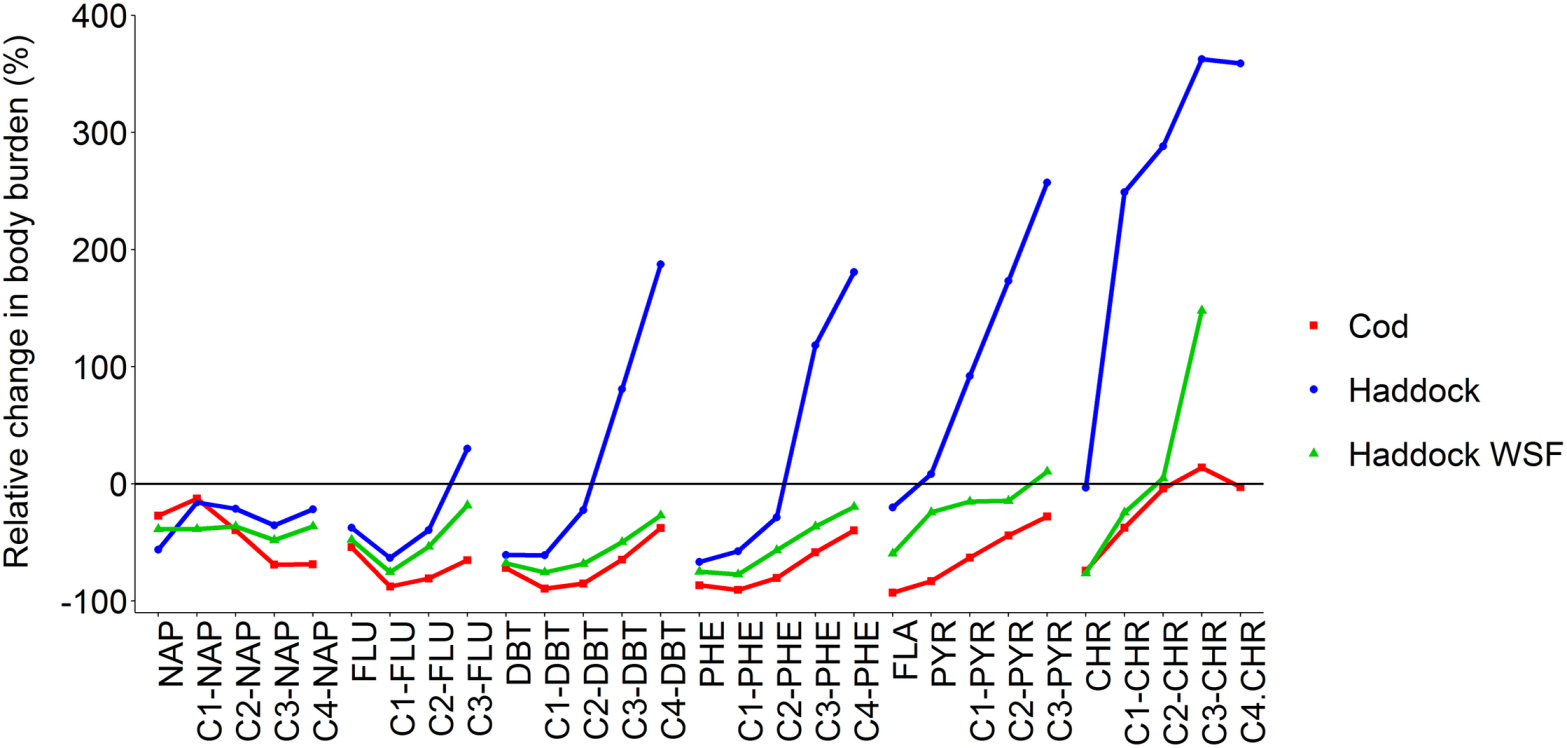
Deviation in body burden (pg/embryo) of PAHs and alkyl PAH groups between day 9 and day 3 for an intermediate exposure concentration (~3 μg/L tPAH). Negative values indicate a decline in body burden of the compound group between the two time points. NAP = naphthalene, FLU = fluorene, DBT = dibenzothiophene, PYR = pyrene, CHR = chrysene.

The drop in tPAH observed in cod embryos between day 3 and day 9 occurred despite continuous exposure, and was unexpected. We therefore examined this in more detail for individual compounds. To investigate the significance of compound structure (ring size and degree of alkylation) on this change in body burden over time, we calculated the relative change (in %) for different compounds groups at day 9 relative to day 3 (Fig 7 and S6 Fig). For cod at all doses (S6B Fig) and non-fouled haddock eggs (exposed to WSF and the lowest dispersed oil dose, tPAH = 0.21 μg/L; S6A Fig), the body burden of all compound groups decreased over time. However, in all treatments there was a lower decrease in body burden for 3 and 4-ring PAHs with increasing alkylation (alkyl-fluorenes, -dibenzothiophenes, -phenanthrenes, -chrysenes and -pyrenes/fluoranthenes). The trend was the opposite for naphthalenes, with increasing alkylation leading to a larger decrease in body burden. Although the body burden of all alkylated 4-ring PAHs and C3-C4 alkylated 3-ring PAHs increased dramatically from day 3 to day 9 for droplet-fouled haddock eggs, concentrations of parent 3-ring, C1-alkylated, and in some cases C2-alkylated homologs decreased (Fig 7 and S6A Fig). Thus, although tPAH continued to increase with continuous oil droplet exposure for haddock, for some individual compounds concentrations declined.

**Fig 8:**
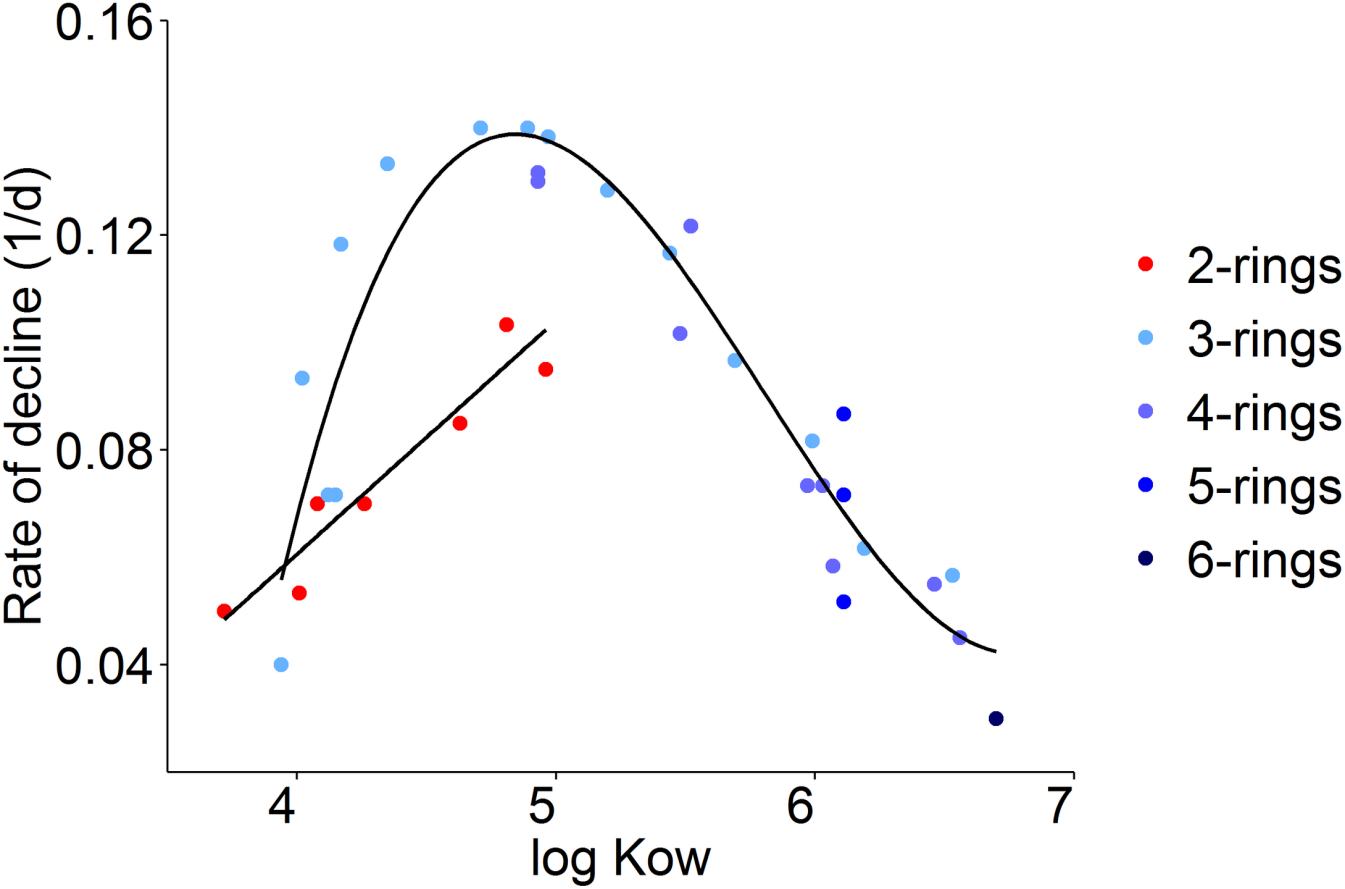
Observed relationship between log *K*_OW_ and rate of decline in body burden concentration from day 3 to day 9 during exposure of crude oil exposed haddock embryos. The fitted line shows a third-degree polynomial fit for 3–6 ring compounds (blue circles). 2-ring PAHs (red circles) showed a linear increase with increasing log *K*_OW_.

To examine this phenomenon in further detail, we plotted the rate of decline of cod embryo body burden from day 3 to day 9 as a function of log *K*_OW_ for all reliably measured compounds (Fig 8). The maximum decline was observed for compounds of intermediate log *K*_OW_ (~5). The values for 3–6 ring compounds (blue shades) could be fitted to a third-degree polynomial function, but 2-ring PAHs (red) did not fit this model well and a linear function is fitted to these data. A similar relationship was observed for both cod and haddock embryos using principal component analysis (PCA) (S8 Fig). The temporal changes in body burden profile in the cod and haddock embryos during the exposure experiment were clearly different. At maximum uptake (day 3), the uptake profile in all embryos was dominated by less alkylated 3-rings compounds (fluorenes and phenanthrenes). At day 9, the embryo-associated chrysenes and heavily alkylated chrysenes and pyrenes increased significantly in medium and high dose droplet-exposed haddock, while the body burden of all larger compounds decreased in cod and WSF exposed haddock embryos. The body burden of the latter was dominated by smaller compounds, mainly 2-ringed naphthalenes and benzothiophenes. At day 10/11, after the loss of chorion, the PAH profile in cod and WSF-exposed haddock was more similar to the profile at day 9. However, the body burden in hatched haddock larvae from the droplet exposure reverted to a profile more similar to what was observed at day 3, with a dominance of intermediate sized compounds.

**Fig 9:**
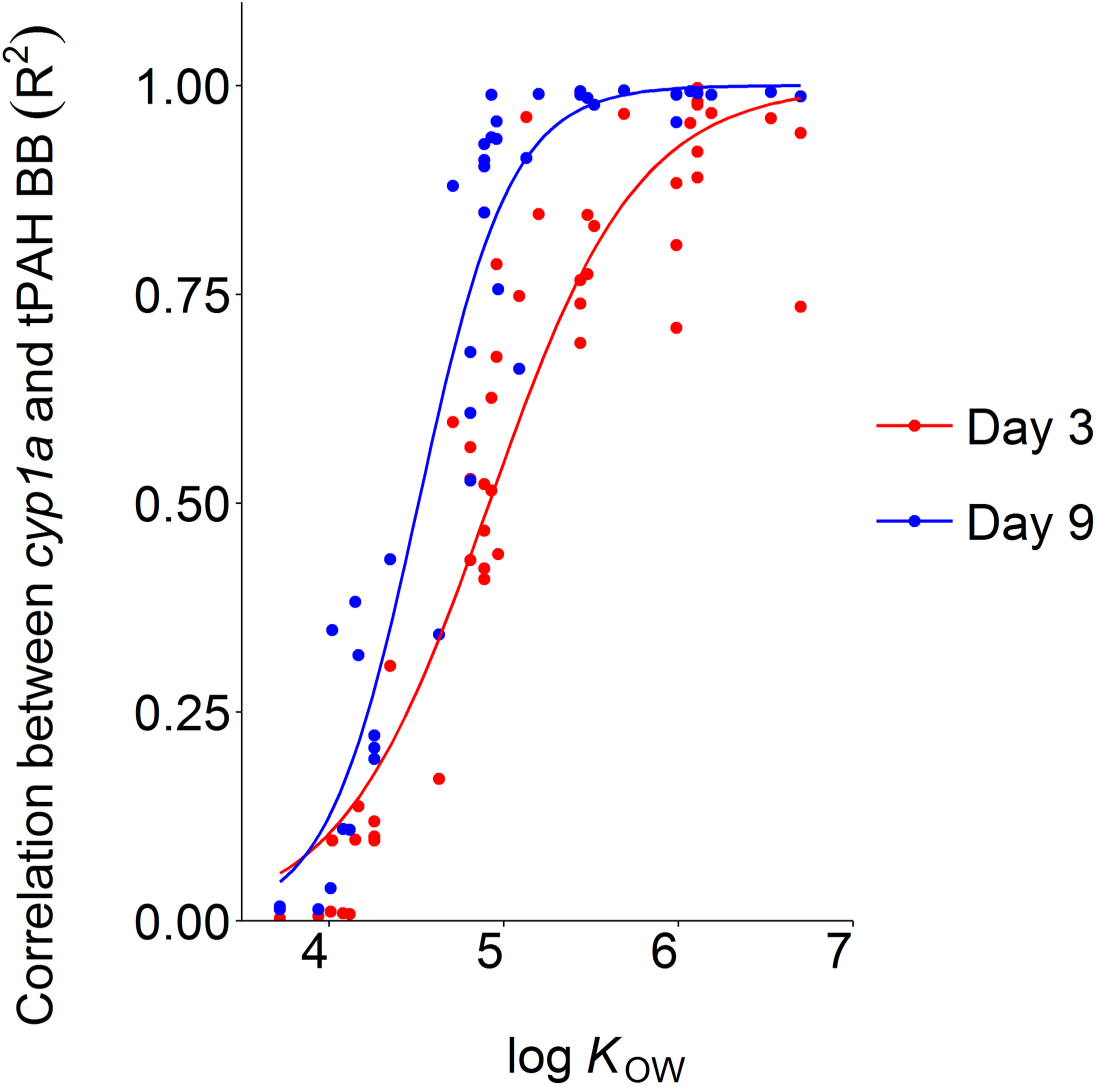
Coefficient of determination (R2) as a function of log KOW for the fit of a linear relationship between cyp1a induction and single compound body burdens.

Finally, we related the log *K*_OW_ of PAHs associated with embryos to levels of *cyp1a* induction. Using the combined PAH data from both haddock (0.10, 0.76, 1.6 (WSF), 2.7 and 3.5 μg/L tPAH) and cod (0.15, 0.29, 2.8, 3.6 and 9.1 μg/L tPAH) (S6 Table), we performed a “blind” correlation of PAH body burden (both total, individual compounds and group clusters) at day 3 and day 9 with *cyp1a* induction measured immediately prior to hatching (Fig 9). A strong correlation (*R*^*2*^ > 0.8) was observed for most compounds with log *K*_OW_ > 5.

## Discussion

### Oil droplet fouling on the haddock egg chorion causes increased internal body burden and more severe toxicity

While it was previously demonstrated that the chorions of haddock embryos are fouled by crude oil droplets upon exposure [3, 19], here we directly compare the closely related species Atlantic cod, and expand on prior work with toxicokinetic data on PAHs. We confirm that haddock eggs are fouled by crude oil, but the same phenomenon was not observed for cod eggs. In the present study we started the exposure of the egg shortly after fertilization and the oil droplets were evenly distributed across the entire haddock chorion, in line with Sørhus et al [19]. In a previous study where exposure of haddock embryos began at a later stage of development, oil droplets were found only to bind at discrete localizations of the chorion [3]. This suggests that the “stickiness” of the surface of the haddock chorion changes throughout embryonic development and declines close to hatching. Measurement of presence and accumulation of *n*-alkanes in the embryo extracts confirmed that only haddock eggs experienced oil droplet fouling. Moreover, *n*-alkane quantification indicated a continuous, linear adhesion uptake of whole oil to haddock embryos, without saturation even at the highest tested dose. However, the lowest dispersed oil concentrations (< 0.7 μg/L tPAH) did not lead to oil fouling on the haddock chorion. This is most likely because such low doses have a limited number of droplets present in the exposure volume, and the statistical chance of egg-droplet interaction is thus low.

Like other species with pelagic eggs, haddock and cod have a thin homogeneous, lamellated chorion [45, 46]. However, haddock eggs have an additional membrane of adhesive material covering the primary egg envelope [46]. Ongoing work includes a chemical characterization of the haddock egg outer membrane, but at present the physical-chemical mechanism of oil droplet fouling is unknown. Nevertheless, these findings emphasize the contribution of the structurally distinct haddock chorion [46] to an interaction with oil droplets that has profound consequences for toxicity. Moreover, the concentrations of dispersed oil leading to toxicity in both species are extremely low and environmentally realistic (< 10 μg/L tPAH). The toxicokinetic data indicate that oil droplet fouling on the haddock chorion influences PAH uptake both quantitatively and qualitatively.

The sensitivity of the haddock embryos observed in the current study was in line with what was observed in previous studies [3, 19, 20], while cod embryos exposed to the same concentrations of crude oil droplets were much less affected. The severity of effects observed in cod embryos was similar to WSF exposed haddock eggs at the same PAH dose (Figs 2 and 3). These results support the hypothesis that cod embryos are mainly affected by the water-soluble fraction of crude oil PAHs, as previously proposed for other species [16, 17, 47]. Further, it indicates that it is the attachment of crude oil droplets on the haddock chorion which is the outer driving force for the increased sensitivity observed in haddock embryos. We propose that the attachment of crude oil particles to the chorion leads to a secondary exposure pathway, facilitating increased uptake of PAHs in the embryos, which further causes the increased severity of deformation and cardiotoxicity in haddock compared to cod.

#### The influence of chorion fouling on internal body burden in haddock embryos

From day 3 to day 9, the oil-fouled haddock eggs showed a significantly different accumulation pattern than the haddock eggs that have only been subject to the WSF and the cod eggs that were not fouled (Fig 7). The measured body burden of (particularly) heavier PAHs is significantly affected by the PAHs found in the droplets that adhere on the egg surface. For the heaviest compounds (log *K*_OW_ > 6) at the highest exposure dose (9 μg/L) the uptake pattern is strikingly similar to the increase in fouling as measured by *n*-alkanes (comparing Fig 1 and S5 Fig), indicating that the main burden of these compounds is found in the droplets on the egg exterior. However, through measurements of accumulated body burden in hatched larvae, and in dechorionated embryos from oil-fouled haddock eggs, we show that adhered oil droplets also lead to an increased internal body burden of PAHs.

Investigation of PAH profiles in cod and haddock embryos at day 3, 9 and 10/11 indicate that all PAHs measured in the cod and haddock WSF samples are mainly located in the internal embryo, as the PAH profiles do not significantly change after hatching (between days 9 and 10/11). However, the profile in hatched, chorion-free haddock embryos from oil droplet exposed eggs revert to a PAH profile more similar to the uptake at day 3 (S8 Fig). This indicates two things. First, the heavier compounds used in the analysis (alkylated 4 ring compounds) have been located mainly on the exterior of the chorion of the haddock eggs. Secondly, the presence of the oil droplet on the chorion during exposure has led to increased internal uptake of alkylated three ring and some four ring compounds. Quantitative investigation of the same samples (Fig 5), as well as samples of dechorionated embryos and hatched larvae from the highest exposure dose (Fig 6) confirm this interpretation. Haddock larvae from crude oil droplet exposures are left with a surplus of larger (> 3-ring) compounds compared to cod and WSF exposed haddock. The increased uptake of heavier PAHs in particular may explain the much higher *cyp1a* induction in haddock embryos, as it is well established that heavier PAHs are more potent *cyp1a* inducers [48–50].

### PAH elimination has major impact on bioaccumulation in fish ELS

While PAH metabolism is known to be an efficient and rapid process in adult and juvenile fish [51–53], it has not previously been considered an important factor in determining the bioaccumulation of PAHs in fish ELS [24, 54]. An unexpected observation in the current study, most obvious in the non-oil-fouled cod eggs and WSF exposed haddock eggs, was that the concentration of internal PAHs generally declined through incubation despite constant exposure (Fig 4 and Table 1). Although the association of PAHs in whole oil on the haddock chorion makes this determination more complicated, careful analysis of individual compounds showed the same pattern.

After peak uptake at day 3, the body burden of all measured PAHs and alkyl PAHs in non-fouled eggs (cod and WSF exposed haddock) declined towards the end of exposure. Both species showed a much higher decline in body burden for non-alkylated compounds relative to the corresponding alkylated homologs. For 3 and 4 ring compounds, there was an inverse relationship between increasing alkylation and loss of PAH body burden (Fig 7). This is in line with previous observations on adult fish [55]. In the current study, we also observed a relationship between log *K*_OW_ and elimination, with maximum decline observed for compounds with log *K*_OW_ close to 5 (Fig 8). 2-ring PAHs showed a different elimination behavior than larger PAHs, where increased alkylation had less impact on the elimination rate (Figs 7 and 8). No steady-state in embryo body burden was reached for compounds below a log *K*_OW_ value of 5 (S3–S5 Figs). For larger compounds (log *K*_OW_ > 5), a steady-state condition was reached after 5–7 days of exposure and only a smaller change in body burden until exposure end at day 11 (after hatching). Given that *cyp1a* is robustly induced by embryos (even at early stages of life and after short exposures [19]), we interpret the observed elimination of PAHs to indicate that xenobiotic metabolism is induced in the embryos. This is in line with previous observations of the effect of retene (a C4-phenanthrene) on rainbow trout ELS [56]. The current experimental design did not allow for a direct, un-biased comparison of PAH elimination or metabolism rates in haddock and cod, and it’s potential impact on toxicity in the two species. Future work should address this by designing experiments where direct, physical interaction between haddock eggs and oil droplets are not possible.

The main reason why the dramatic influence of elimination on PAH accumulation in fish embryos has not often previously been reported, is partly due to the use of radiolabeled chemicals in uptake studies [24, 54, 57]. The use of such chemicals would lead to the co-determination of PAH and all PAH metabolites, and give no information of parent PAH degradation within the egg/embryo. In the current study, elimination affected all PAHs and alkyl PAH compounds groups studied, which is a larger suite of compounds than what have ever been reported in a single study previously. The proposed cause of elimination is biotransformation, metabolism in the embryos starting as early as 3–4 dpf. However, no information about potential degradation products is obtained in the current study. From previous studies, it is known that metabolic products predominantly produced may be dependent upon both PAH structure and species [58–61]. Future studies should focus on confirming the metabolism potential of fish ELS, as well as identifying the metabolites of alkyl and parent PAHs formed in embryos and larvae of different species.

## Conclusion

In the current study, the change in PAH body burden in dispersed crude oil exposed Atlantic haddock and cod embryos during a 10 or 11 days long exposure (1-11/12 days post fertilization (dpf)) was investigated. It was determined that haddock eggs, unlike cod, are fouled by crude oil droplets adhering to the chorion when exposed to concentrations > 0.7 μg/L tPAH. This is correlated with an increased body burden of both parent and alkylated PAHs, and a severe increase in toxicological responses (malformations and cardiotoxicity). Due to the fouling of the haddock chorion with crude oil droplets, total measured egg-associated PAHs prior to hatching is higher (particularly for heavier compounds) than what is actually bioavailable to the embryos, and much higher than what is measured in the non-fouled cod eggs. However, the increased *cyp1a* induction and increased severity of cardiotoxicity and morphological malformations in haddock compared to cod clearly indicate an increased uptake of PAHs in the embryos. This is consistent with a secondary uptake pathway caused by the oil droplets adhering to the surface of the eggs.

This work also highlights the importance of considering the influence of elimination or transformation of PAHs on measured body burdens, even when working with earliest life stages of fish. Both haddock and cod embryos are able to eliminate a wide range of both parent and alkylated PAHs, starting as early as 3–4 dpf when exposed to crude oil dispersions. Furthermore, future research should address the knowledge gap concerning the potential toxicity of PAH metabolites formed in fish ELS.

For the purpose of oil spill damage assessment models, it is desirable to be able to predict the body residue of toxic oil compounds (such as PAHs) in the body of organisms of concern in a given spill scenario. However, as seen for the case of cod and haddock embryos, the body burden of PAHs may be highly dynamic, even in the case of a constant exposure over a reasonably long time. This must be taken into consideration when planning experiments to provide input into crude oil effects models.

## Supporting information

S1 Fig Exposure and sampling regime. Timepoints shaded in grey mark sampling dates for body burden measurements.
(TIF)

S2 Fig Uptake of sum PAHs over nine days. Sum of single compounds measured in eggs during exposure at three doses (A = high, ~9 μg/L, B = medium, ~3 μg/L, C = low, ~0.3 μg/L tPAH). Error bars represent one standard deviation (*n* = 3).
(TIF)

S3 Fig Uptake of PAHs in low (~0.3 μg/L) dose dispersion exposure. Uptake of 28 single PAHs in low dose crude oil exposed haddock (0.21 μg/L tPAH) and cod (0.29 μg/L tPAH) embryos over nine days of exposure. Error bars represent one standard deviation (*n* = 3).
(TIF)

S4 Fig Uptake of PAHs in medium (~3 μg/L tPAH) dose dispersion exposure. Uptake of 28 single PAHs in medium dose crude oil exposed haddock (2.7 μg/L tPAH) and cod (2.9 μg/L tPAH) embryos, as well as water-soluble fraction (WSF) exposed haddock embryos (1.6 μg/L tPAH) over 10 (haddock) or 11 (cod) days of exposure, followed by two days in clean water. Error bars represent one standard deviation (*n* = 3).
(TIF)

S5 Fig Uptake of PAHs in high (~9 μg/L) dose dispersion exposure. Uptake of 28 single PAHs in high dose crude oil exposed haddock (8.6 μg/L tPAH) and cod (9.1 μg/L tPAH) embryos over 10 (haddock) or 11 (cod) days of exposure, followed by two days in clean water for cod. Haddock embryos did not survive hatching in sufficient numbers to be followed further. Error bars represent one standard deviation (*n* = 3).
(TIF)

S6 Fig Relative change in body burden from day 3 to day 9 (%). Deviation in body burden (pg/embryo) of PAHs and alkyl PAH groups between day 9 and day 3 for several exposure concentrations for both haddock (A) and cod (B). Negative values indicate a decline in body burden of the compound group between the two time-points, indicating dominating influence metabolic transformation.
(TIF)

S7 Fig Total body burden. Total body burden (sum of individual PAHs and alkyl PAH clusters) at maximum measured uptake (day 3), last day of embryo sampling (day 9) and exposure end (day 10 for haddock, day 11 for cod). Error bars represent one standard deviation (*n* = 3).
(TIF)

S8 Fig Principal component analysis (PCA) comparing the uptake profile of PAHs. Comparing the change in body burden PAH profile from the point of measured maximum tPAH uptake (day 3), and before (day 9) and after hatching (day 10 (haddock), 11 (cod)). PCA was performed on individual compound or compound group concentrations (pg/embryo) normalized to tPAH, scaled to the mean of each variable and centered.
(TIF)

S9 Fig Principal component analysis (PCA) comparing the uptake profile of PAHs in cod and haddock over time. Comparing the change in body burden PAH profile over the course of the exposure for cod and haddock embryos separately. PCA was performed on individual compound or compound group concentrations (pg/embryo) normalized to tPAH, scaled to the mean of each variable and centered.
(TIF)

S10 Fig Comparison of percentage of different PAH groups recovered on the chorions, manually extracted embryo and hatched larvae relative to the total in intact haddock eggs fouled by oil droplets. Eggs were sampled immediately prior to hatch, and hatched larvae immediately post-hatch.
(TIF)

S1 Table Properties of PAH analytes and their GC-MS/MS analytical conditions. (DOC)

S2 Table Real time qPCR primer and probe sequences for *cyp1a*. (DOC)

S3 Table Measured PAH exposure concentrations. Concentration of individual and alkyl cluster PAHs measured in water samples (ng/L) of the individual exposure groups, and in the crude oil (μg/g) used in the experiments, given with one standard deviation (*n* = 3). LOQ = limit of quantification.
(DOC)

S4 Table Alkane uptake. Relative response of alkanes (Σ(*n*C19-*n*C32)) normalized to the response of internal standard pyrene-*d*12. Given with one standard deviation (*n* = 3).
(DOC)

S5 Table Characterization of cardiac function and morphology at 2 dph (cod) and 3 dph (haddock). ^1^VFS, ventriclular fractional shortening; AFS, atrial fractional shortening; SV, silent ventricle. ^a–d^Letters indicate significant differences between groups within the same experiment (*p* = <0.05) (groups with same letters are not significantly different from each other). *very underdeveloped ventricle treated as silent ventricle.
(DOC)

S6 Table Correlation between Cyp1a and PAH body burden. Linear regression performed on data sets of body burden (pg) embryo and Cyp1a fold change normalized to control in for both cod and haddock at day 3 and day 9. Correlation expressed in terms of the coefficient of determination (*R*^*2*^) for the regression. ND = not determined (too many missing body burden values).
(DOC)

S7 Table Body burden data. Uptake of PAHs and alkylated PAHs in cod and haddock embryos. Given as pg/embryo with one SD (*n* = 3). LOQ = limit of quantification. NA = not analysed.
(DOC)

S1 File NC3Rs ARRIVE guidelines checklist. (PDF)
