# Correction: Oil droplet fouling and differential toxicokinetics of polycyclic aromatic hydrocarbons in embryos of Atlantic haddock and cod

**DOI:** 10.1371/journal.pone.0343487

**Published:** 2026-02-23

**Authors:** Lisbet Sørensen, Elin Sørhus, Trond Nordtug, John P. Incardona, Tiffany L. Linbo, Laura Giovanetti, Ørjan Karlsen, Sonnich Meier

After publication of this article [1], the authors notified the journal office that some data in Table 1 and S5 Table are incorrect.

The error in Table 1 is in the columns concerning total PAH body burden reported on a ng/g wet weight and ng/g lipid weight basis. The incorrect number of compounds was summed when calculating the ng/g wet weight. Only single compounds were included in the sum, not clusters as the caption describes. This error led to the authors reporting lower values for ng/g wet weight body burden than they measured. Furthermore, the error propagated to the ng/g lipid weight values because these were calculated from the ng/g wet weight by using the average lipid content (7%) of the eggs on a mass basis.

The errors in S5 Table are from errors in copy-pasting while preparing the table. The values for all exposure groups in section Haddock 2 are incorrect in the original S5 Table. The corrections are minor and do not change the overall outcome of the table or the interpretations in the article.

A member of the *PLOS One* Editorial Board reviewed the corrections to both tables and agrees that the changes do not affect the conclusions and results of the study.

An updated, correct version of S5 Table is included with this notice.

Please see the corrected Table 1 here.

**Table 1:** Total PAH body burden (including alkyl clusters) measured in cod and haddock eggs at day 3 and day 9 of exposure.

	CW (µg/L)	Body burden (pg/embryo)						Body burden (ng/g wet weight)						Body burden (ng/g lipid)					
		Day 3			Day 9			Day 3			Day 9			Day 3			Day 9		
**Cod**	0.15 ± 0.01	101	±	33	32.4	±	7.6	60	±	20	19	±	4	8 572	±	2 857	2 714	±	571
	0.29 ± 0.03	147	±	15	91	±	11	86	±	13	54	±	5	12 286	±	1 857	7 714	±	714
	2.8 ± 0.5	1 902	±	88	639	±	98	1 126	±	48	368	±	63	160 860	±	6 857	52 572	±	9 000
	3.6 ± 0.6	1 560	±	41	1 006	±	193	926	±	78	603	±	96	132 288	±	11 143	86 145	±	13 715
	9.1 ± 1.4	4 455	±	558	1 585	±	153	2 527	±	307	920	±	130	361 007	±	43 858	131 431	±	18 572
**Haddock**	0.09 ± 0.11	239	±	330	78	±	37	115	±	159	40	±	20	16 429	±	22 715	5 714	±	2 857
	0.17 ± 0.06	161	±	96	78.5	±	3.8	79	±	46	41	±	3	11 286	±	6 572	5 857	±	429
	0.21 ± 0.03	238	±	56	153	±	41	115	±	33	79	±	22	16 429	±	4 714	11 286	±	3 143
	0.76 ± 0.09	1 050	±	46	1 128	±	209	492	±	41	587	±	131	70 287	±	5 857	83 859	±	18 715
	2.7 ± 0.5	3 280	±	108	4 106	±	482	1 840	±	90	2 079	±	219	262 862	±	12 857	297 006	±	31 286
	3.5 ± 0.8	4 959	±	199	5 983	±	991	2 694	±	278	3 131	±	384	384 865	±	39 715	447 295	±	54 858
	8.6 ± 1.4	8 790	±	68	13 315	±	2 532	4 177	±	312	6 625	±	1 321	596 726	±	44 572	946 448	±	188 718

## Supporting information

S5 Table Characterization of cardiac function and morphology at 2 dph (cod) and 3 dph (haddock). (DOCX)
