# Supplementary figures and images for: Oil droplet fouling and differential toxicokinetics of polycyclic aromatic hydrocarbons in embryos of Atlantic haddock and cod
Source: PLoS One. 2017 Jul 5;12(7):e0180048. doi: 10.1371/journal.pone.0180048 (PMC5497984; doi:10.1371/journal.pone.0180048)

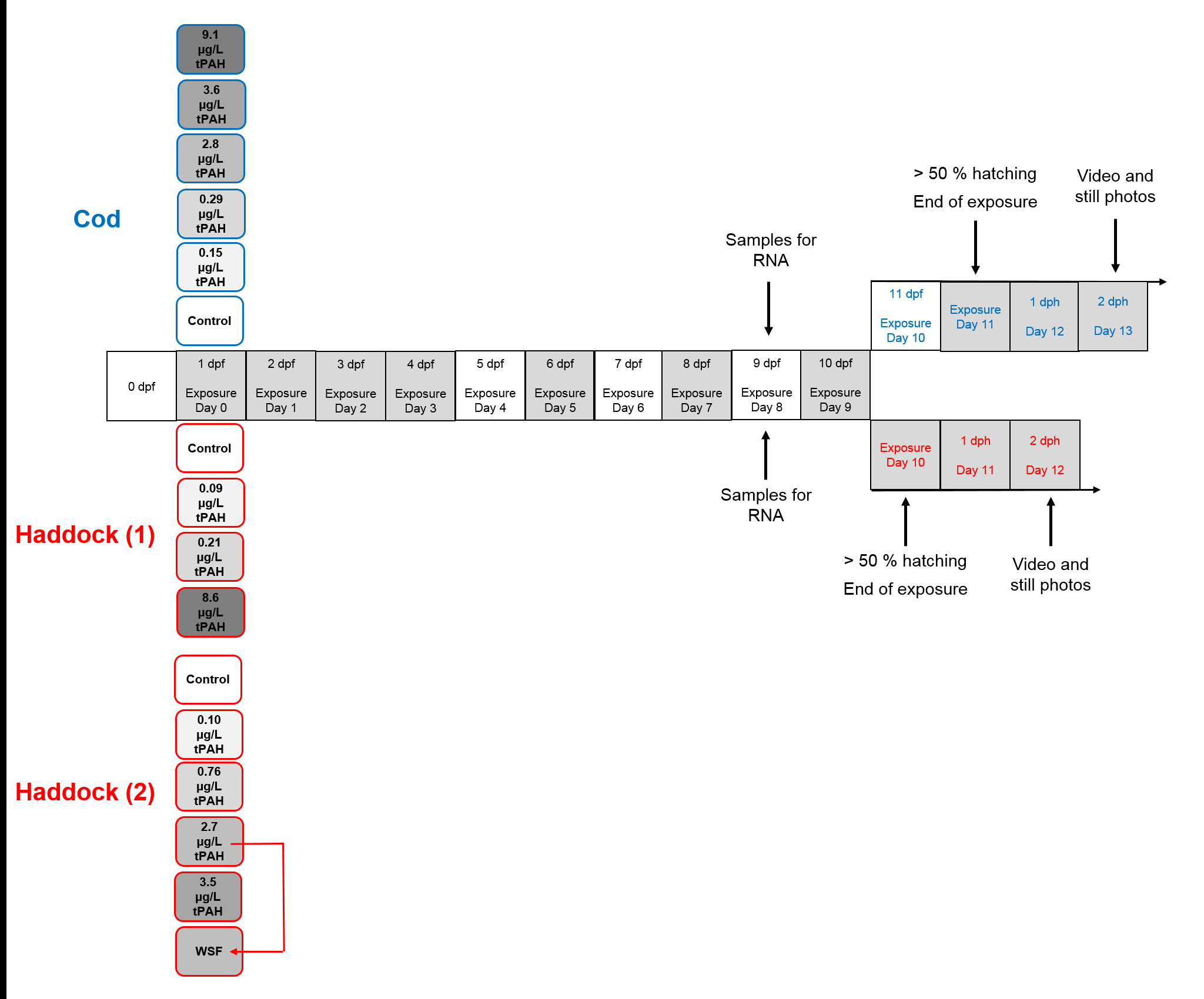

Supplement: S1 Fig — Timepoints shaded in grey mark sampling dates for body burden measurements. (TIF) [file pone.0180048.s001.tif]

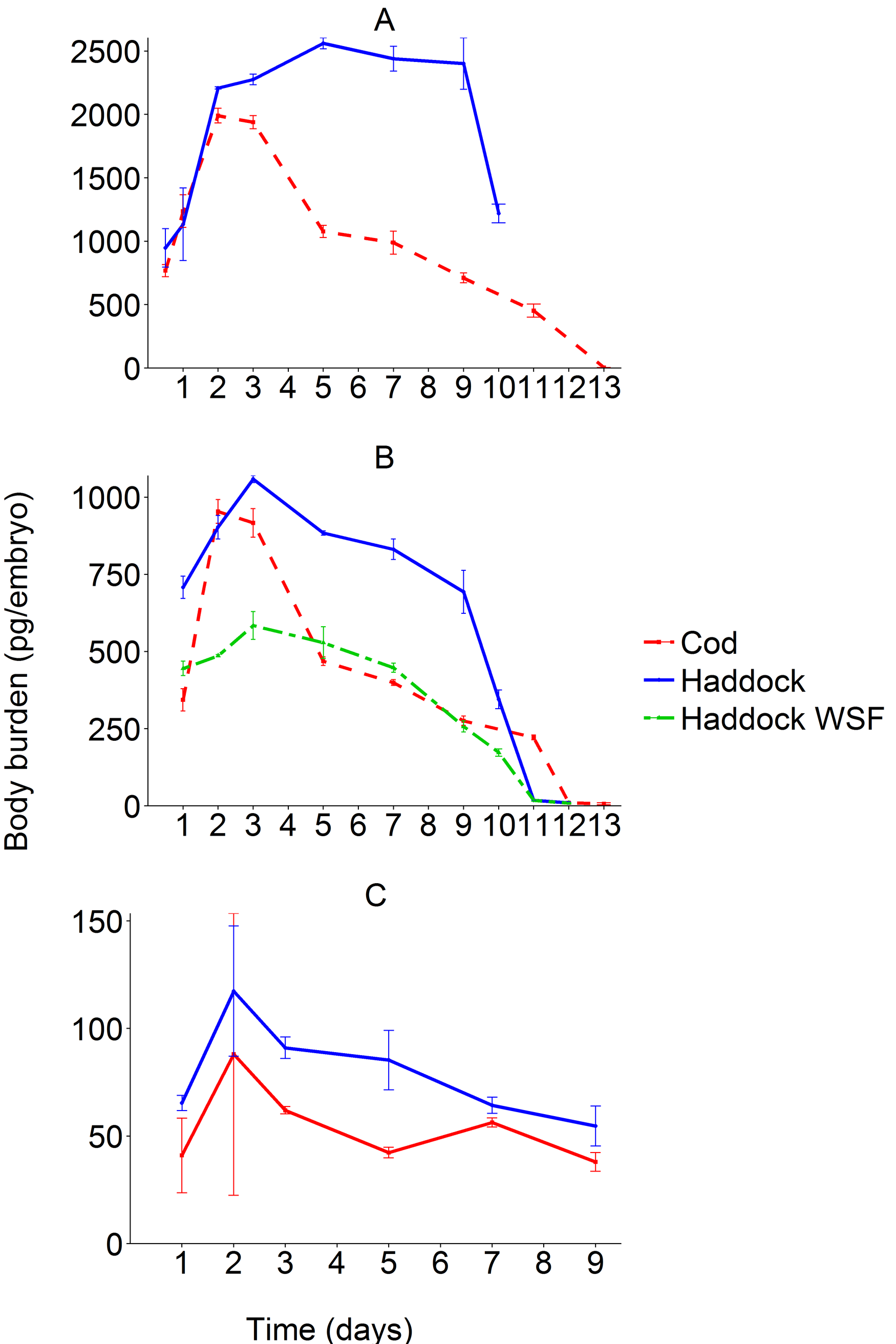

Supplement: S2 Fig — Sum of single compounds measured in eggs during exposure at three doses (A = high, ~9 μg/L, B = medium, ~3 μg/L, C = low, ~0.3 μg/L tPAH). Error bars represent one standard deviation (n = 3). (TIF) [file pone.0180048.s002.tif]

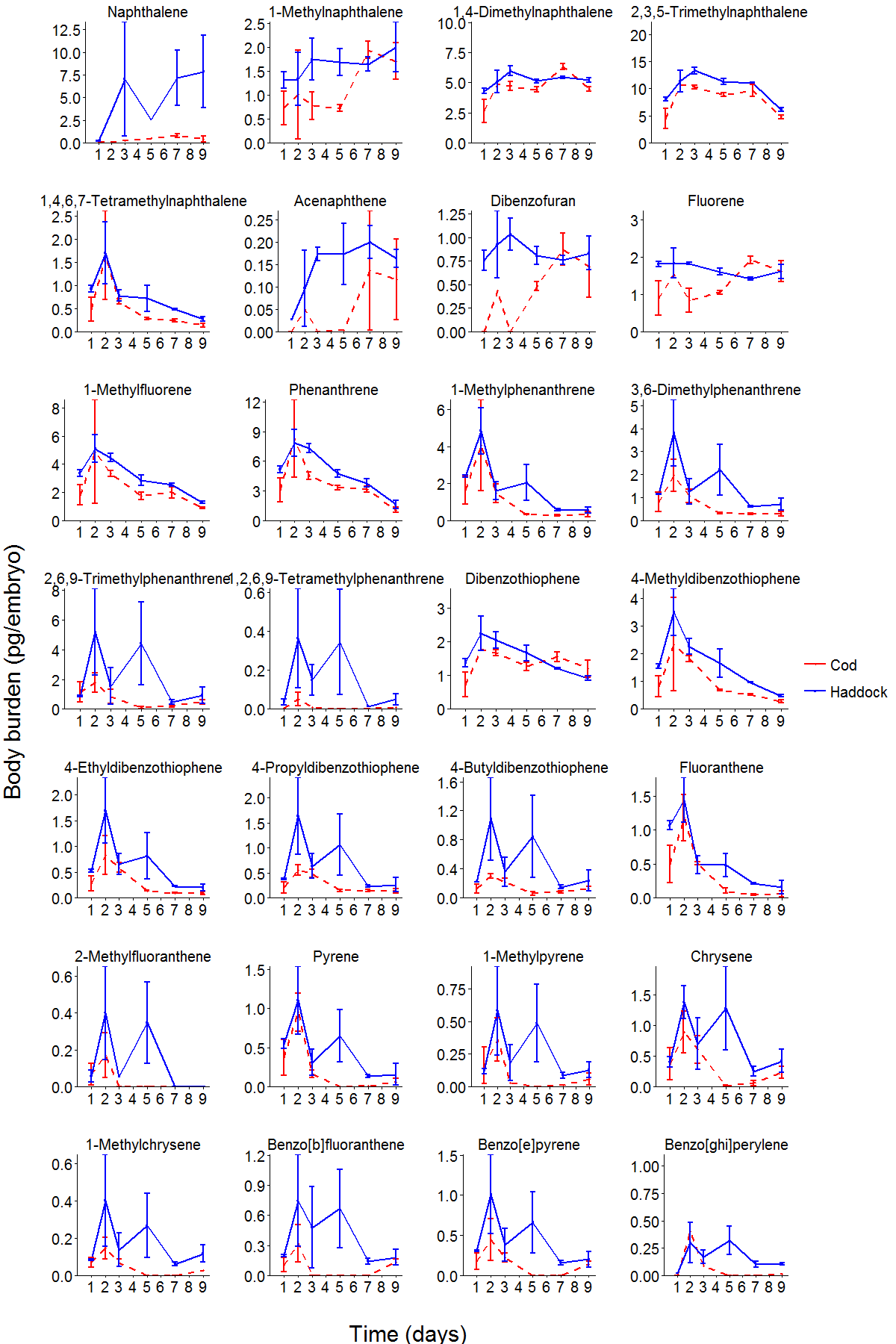

Supplement: S3 Fig — Uptake of 28 single PAHs in low dose crude oil exposed haddock (0.21 μg/L tPAH) and cod (0.29 μg/L tPAH) embryos over nine days of exposure. Error bars represent one standard deviation (n = 3). (TIF) [file pone.0180048.s003.tif]

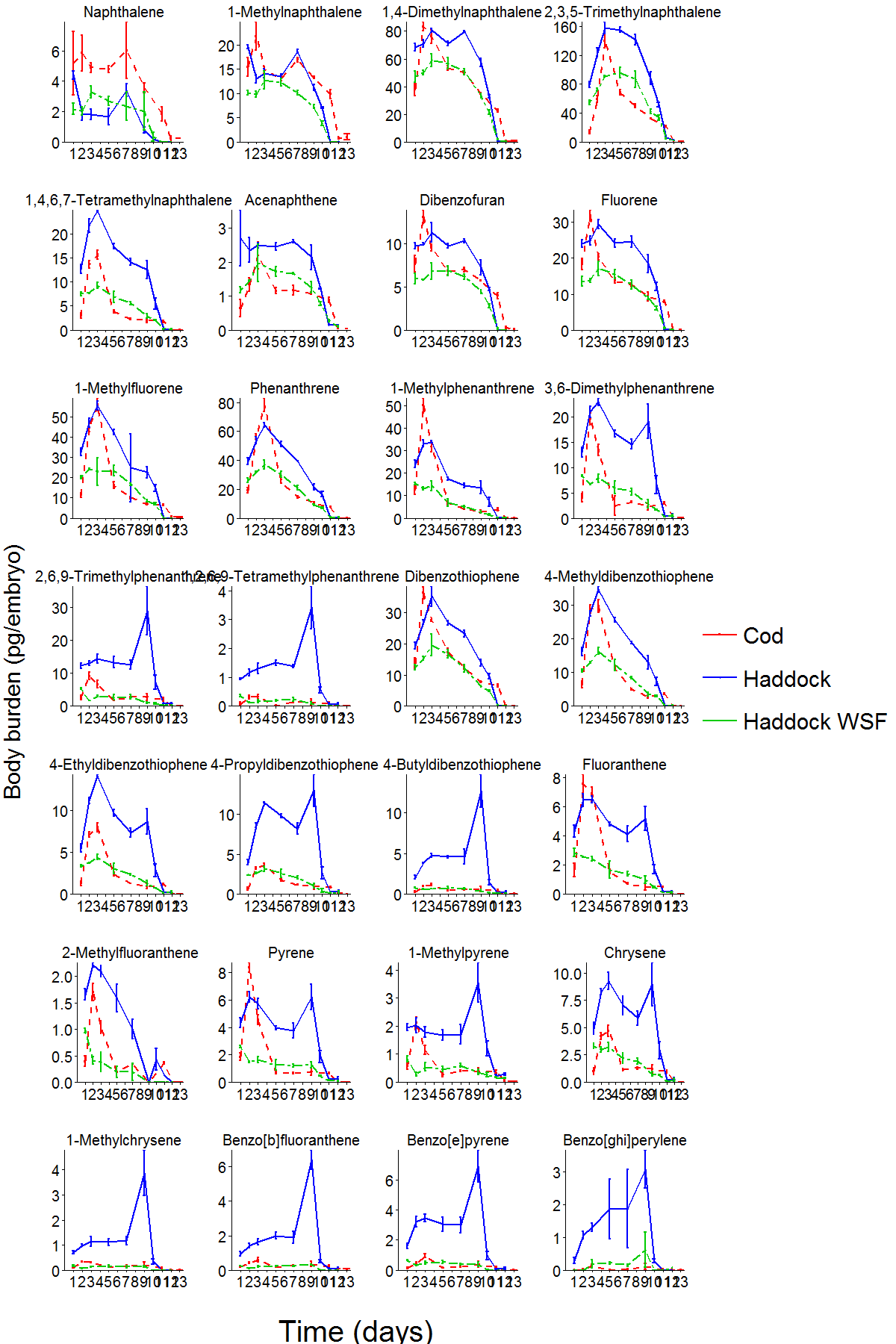

Supplement: S4 Fig — Uptake of 28 single PAHs in medium dose crude oil exposed haddock (2.7 μg/L tPAH) and cod (2.9 μg/L tPAH) embryos, as well as water-soluble fraction (WSF) exposed haddock embryos (1.6 μg/L tPAH) over 10 (haddock) or 11 (cod) days of exposure, followed by two days in clean water. Error bars represent one standard deviation (n = 3). (TIF) [file pone.0180048.s004.tif]

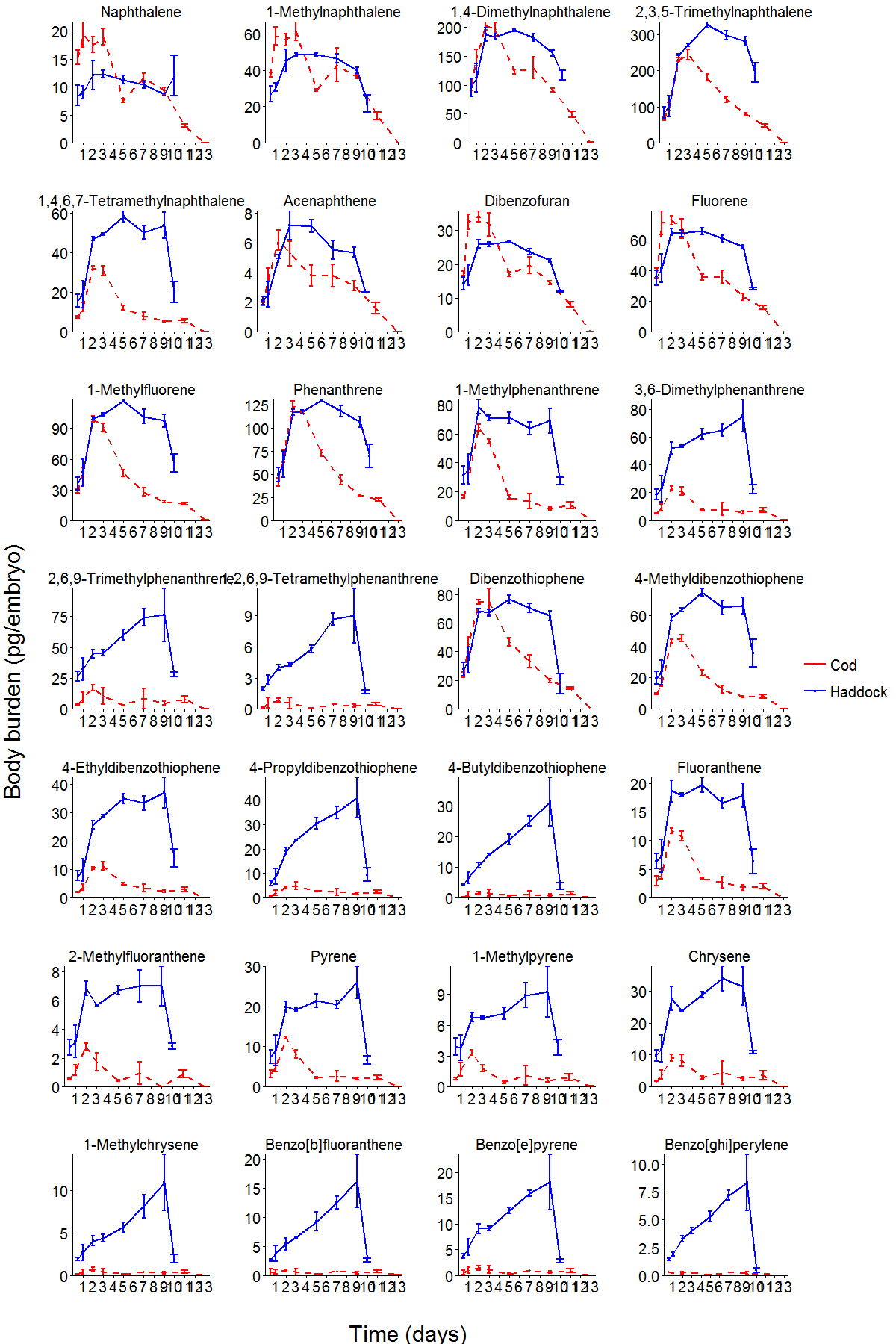

Supplement: S5 Fig — Uptake of 28 single PAHs in high dose crude oil exposed haddock (8.6 μg/L tPAH) and cod (9.1 μg/L tPAH) embryos over 10 (haddock) or 11 (cod) days of exposure, followed by two days in clean water for cod. Haddock embryos did not survive hatching in sufficient numbers to be followed further. Error bars represent one standard deviation (n = 3). (TIF) [file pone.0180048.s005.tif]

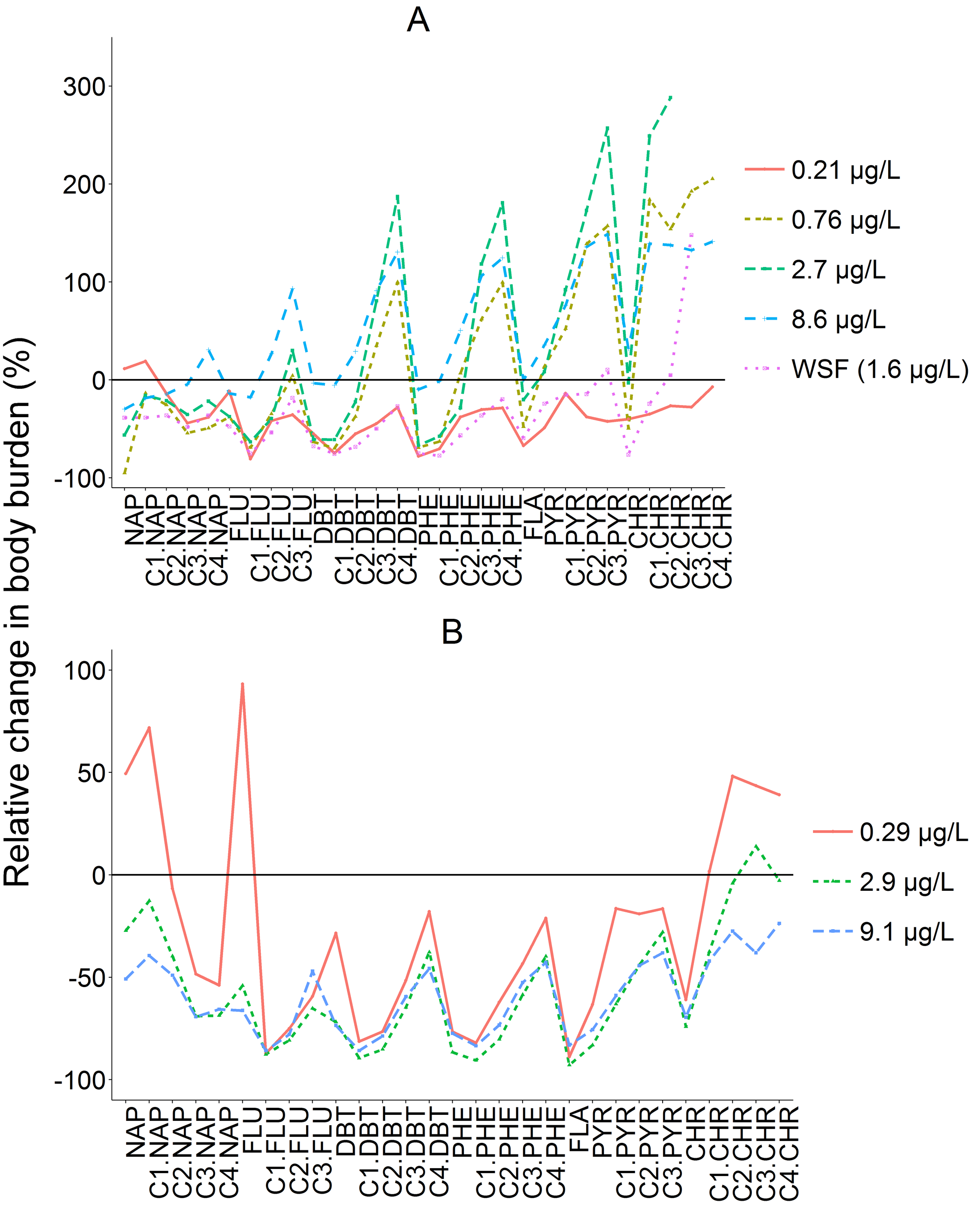

Supplement: S6 Fig — Deviation in body burden (pg/embryo) of PAHs and alkyl PAH groups between day 9 and day 3 for several exposure concentrations for both haddock (A) and cod (B). Negative values indicate a decline in body burden of the compound group between the two time-points, indicating dominating influence metabolic transformation. (TIF) [file pone.0180048.s006.tif]

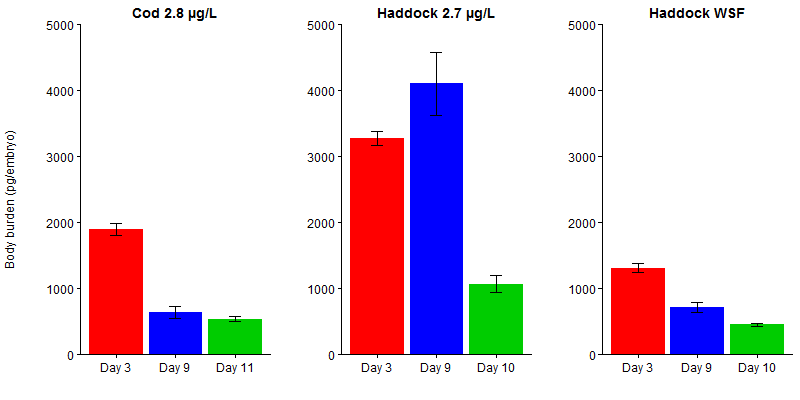

Supplement: S7 Fig — Total body burden (sum of individual PAHs and alkyl PAH clusters) at maximum measured uptake (day 3), last day of embryo sampling (day 9) and exposure end (day 10 for haddock, day 11 for cod). Error bars represent one standard deviation (n = 3). (TIF) [file pone.0180048.s007.tif]

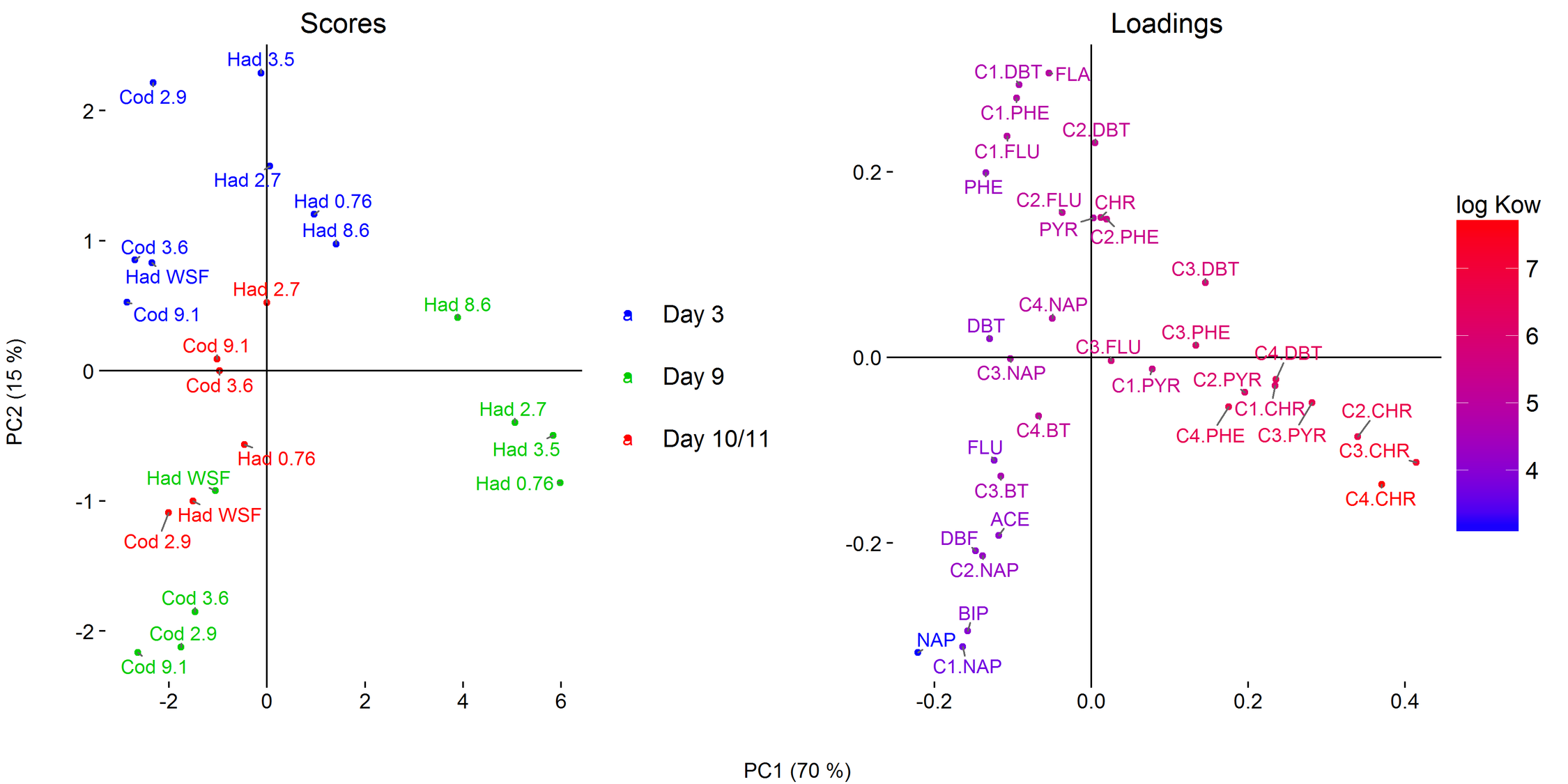

Supplement: S8 Fig — Comparing the change in body burden PAH profile from the point of measured maximum tPAH uptake (day 3), and before (day 9) and after hatching (day 10 (haddock), 11 (cod)). PCA was performed on individual compound or compound group concentrations (pg/embryo) normalized to tPAH, scaled to the mean of each variable and centered. (TIF) [file pone.0180048.s008.tif]

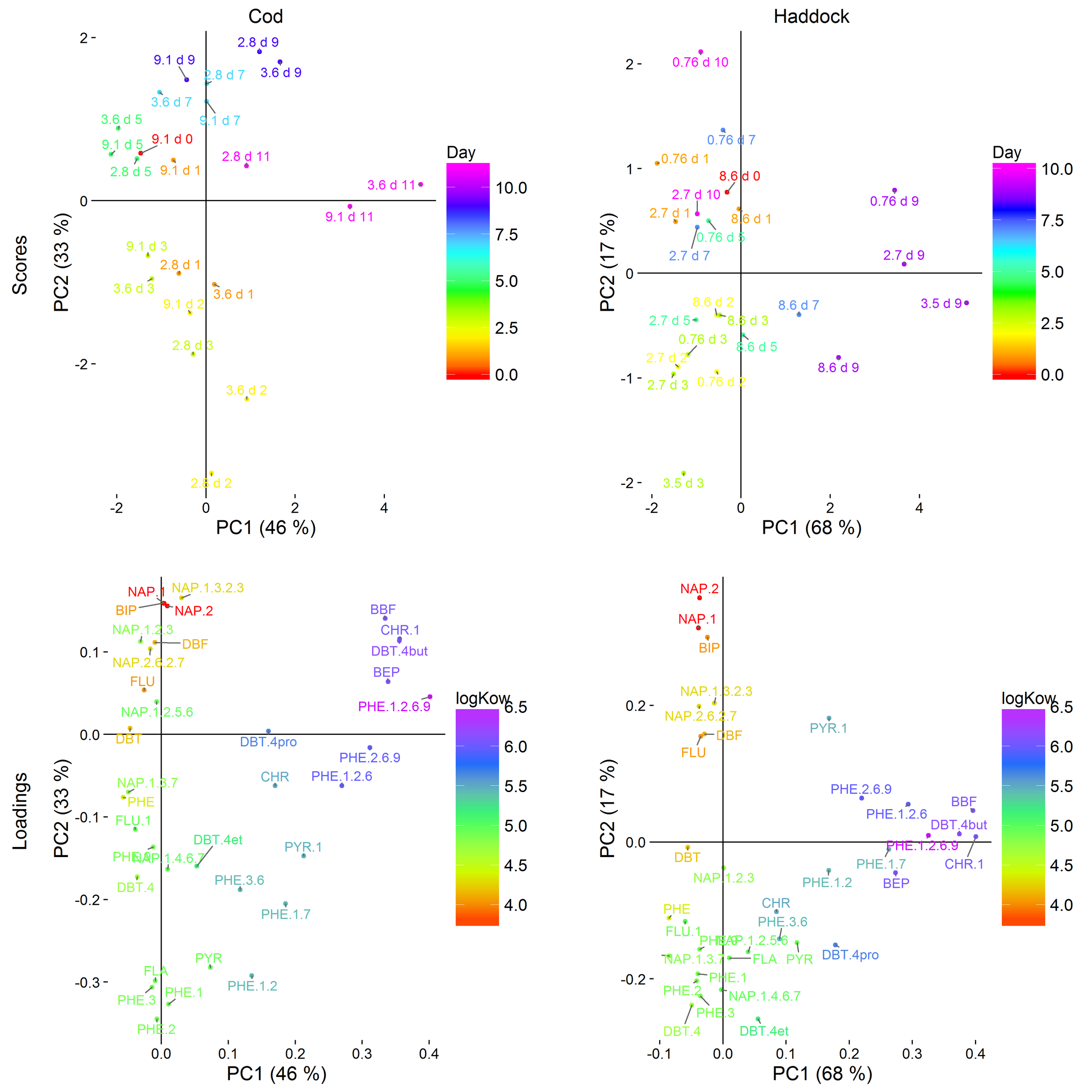

Supplement: S9 Fig — Comparing the change in body burden PAH profile over the course of the exposure for cod and haddock embryos separately. PCA was performed on individual compound or compound group concentrations (pg/embryo) normalized to tPAH, scaled to the mean of each variable and centered. (TIF) [file pone.0180048.s009.tif]

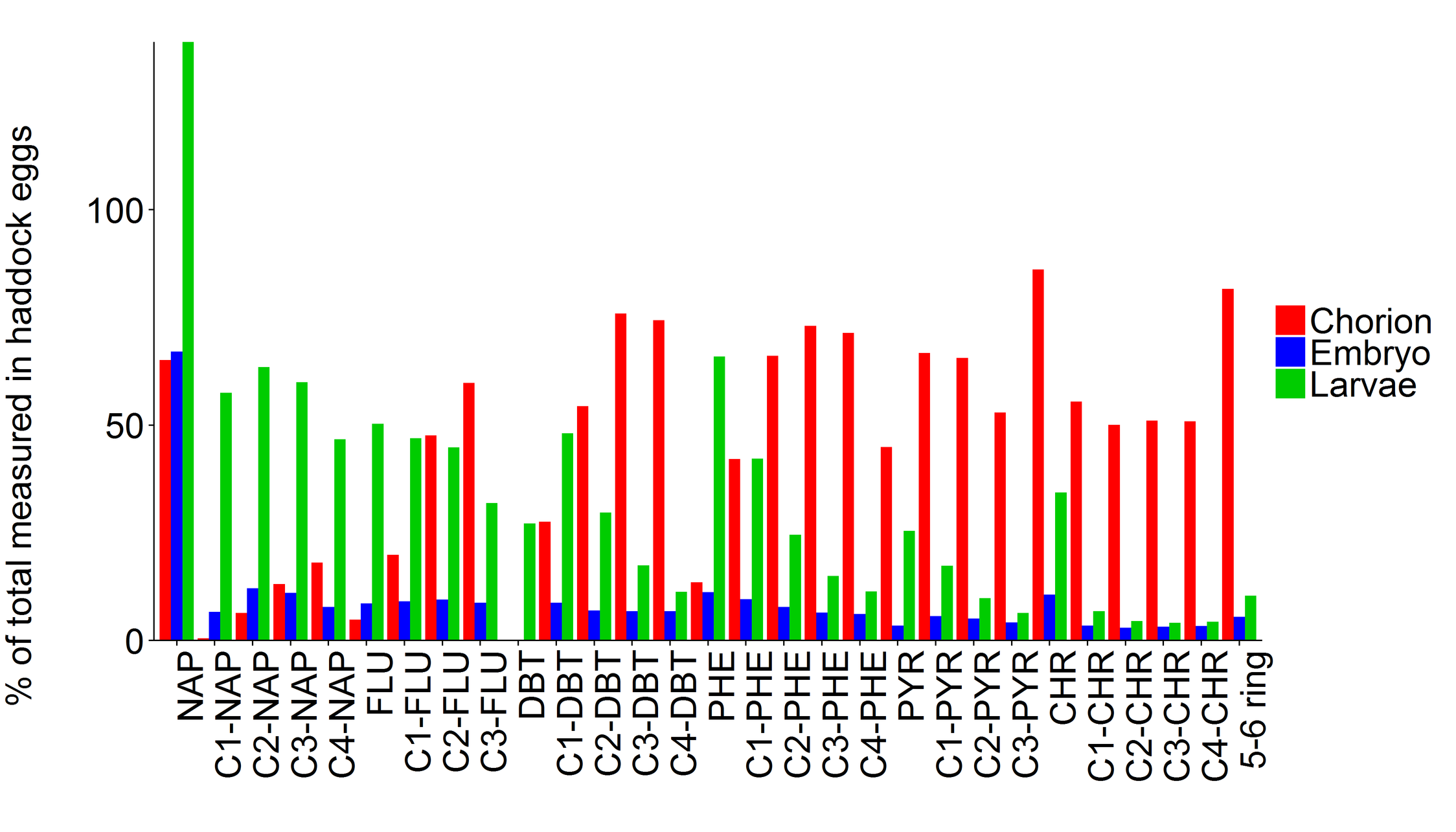

Supplement: S10 Fig — Eggs were sampled immediately prior to hatch, and hatched larvae immediately post-hatch. (TIF) [file pone.0180048.s010.tif]
