# Supplementary material for: Oil droplet fouling and differential toxicokinetics of polycyclic aromatic hydrocarbons in embryos of Atlantic haddock and cod
Source: PLoS One. 2017 Jul 5;12(7):e0180048. doi: 10.1371/journal.pone.0180048 (PMC5497984; doi:10.1371/journal.pone.0180048)
Supplement: S1 Table — (DOC) [file pone.0180048.s011.doc]

**S1 Table. Properties of PAH analytes and their GC-MS/MS analytical conditions.**

|  |  |  |  | **MRM** | **CE** | **MRM** | **CE** |
| --- | --- | --- | --- | --- | --- | --- | --- |
| **Analyte** | **Short** | **Mw (g/mol)** | **log Kow[1]** | **Quant** | **(V)** | **Qual** | **(V)** |
| Biphenyl | BIP | 154.2 | 4.01 | 154-153 | 20 | 154-152 | 30 |
| Benzothiophene | BT | 134.2 | 3.12 | 134-89 | 30 | 134-90 | 30 |
| Dimethylbenzothiophene, 2,5- | BT-2,5 | 162.3 | 4.08 | 162-161 | 15 | 161-128 | 20 |
| Trimethylbenzothiophene, 2,5,7- | BT-2,5,7 | 176.3 | 4.63 | 176-161 | 15 | 176-175 | 20 |
| Naphthalene | NAP | 128.2 | 3.17 | 128-102 | 25 | 128-78 | 25 |
| Methylnaphthalene, 2- | NAP-2 | 142.2 | 3.72 | 142-141 | 20 | 142-115 | 40 |
| Methylnaphthalene, 1- | NAP-1 | 142.2 | 3.72 | 142-141 | 20 | 142-115 | 40 |
| Dimethylnaphthalene, 2,6&2,7- | NAP-2,6&2,7 | 156.2 | 4.26 | 156-141 | 20 | 141-115 | 25 |
| Dimethylnaphthalene, 1,4- | NAP-1,4 | 156.2 | 4.26 | 156-141 | 20 | 141-115 | 25 |
| Dimethylnaphthalene, 1,3&2,3- | NAP-1,3&2,3 | 156.2 | 4.26 | 156-141 | 20 | 141-115 | 25 |
| Trimethylnaphthalene, 1,3,7- | NAP-1,3,7 | 170.3 | 4.81 | 170-155 | 15 | 155-153 | 15 |
| Trimethylnaphthalene, 2,3,5- | NAP-2,3,5 | 170.3 | 4.81 | 170-155 | 15 | 155-153 | 15 |
| Trimethylnaphthalene, 1,2,3- | NAP-1,2,3 | 170.3 | 4.81 | 170-155 | 20 | 155-153 | 20 |
| Tetramethylnaphthalene, 1,2,5,6- | NAP-1,2,5,6 | 184.3 | 4.96 | 184-169 | 20 | 169-154 | 15 |
| Tetramethylnaphthalene, 1,4,6,7- | NAP-1,4,6,7 | 184.3 | 4.96 | 184-169 | 20 | 169-154 | 15 |
| Acenaphthylene | ACY | 152.2 | 3,94 | 152-151 | 25 | 152-150 | 45 |
| Acenaphthene | ACE | 154.2 | 4.15 | 154-153 | 25 | 153-152 | 25 |
| Dibenzofuran | DBF | 168.2 | 4,12 | 168-139 | 30 | 139-89 | 45 |
| Fluorene | FLU | 166.2 | 4.02 | 166-165 | 25 | 165-164 | 25 |
| Ethylfluorene, 9- | FLU-9et | 194.3 | 5.09 | 180-165 | 25 | 165-164 | 20 |
| Methylfluorene, 1- | FLU-1 | 180.3 | 4.97 | 194-165 | 20 | 165-164 | 20 |
| Propylfluorene, 9-n- | FLU-9pro | 208.3 | 5.13 | 208-165 | 30 | 165-164 | 30 |
| Dibenzothiophene | DBT | 184.3 | 4.17 | 184-139 | 45 | 184-152 | 25 |
| Methyldibenzothiophene, 4- | DBT-4 | 198.3 | 4.71 | 198-197 | 20 | 197-165 | 25 |
| Ethyldibenzothiophene, 4- | DBT-4et | 212.3 | 5.20 | 212-197 | 20 | 197-165 | 25 |
| Propyldibenzothiophene, 4-n-, | DBT-4pro | 226.4 | 5.69 | 226-197 | 20 | 197-165 | 25 |
| Butyldibenzothiophene, 4-n- | DBT-4but | 240.4 | 6.19 | 240-197 | 30 | 197-165 | 30 |
| Phenanthrene | PHE | 178.2 | 4.35 | 178-176 | 45 | 178-177 | 30 |
| Anthracene | ANT | 178.2 | 4.35 | 178-176 | 45 | 178-177 | 30 |
| Methylphenanthrene, 3- | PHE-3 | 192.3 | 4.89 | 192-191 | 25 | 191-189 | 25 |
| Methylphenanthrene, 2- | PHE-2 | 192.3 | 4.89 | 192-191 | 25 | 191-189 | 25 |
| Methylphenanthrene, 9- | PHE-9 | 192.3 | 4.89 | 192-191 | 25 | 191-189 | 25 |
| Methylphenanthrene, 1- | PHE-1 | 192.3 | 4.89 | 192-191 | 25 | 191-189 | 25 |
| Dimethylphenanthrene, 3,6- | PHE-3,6 | 206.3 | 5.44 | 206-189 | 45 | 206-191 | 20 |
| Dimethylphenanthrene, 1,7- | PHE-1,7 | 206.3 | 5.44 | 206-189 | 45 | 206-191 | 20 |
| Dimethylphenanthrene, 1,2- | PHE-1,2 | 206.3 | 5.44 | 206-191 | 20 | 206-189 | 45 |
| Trimethylphenanthrene, 2,6,9- | PHE-2,6,9 | 220.3 | 5.99 | 220-205 | 20 | 205-189 | 35 |

S1 Table continued

|  |  |  |  | **MRM** | **CE** | **MRM** | **CE** |
| --- | --- | --- | --- | --- | --- | --- | --- |
| **Analyte** | **Short** | **Mw (g/mol)** | **log Kow** | **Quant** | **(V)** | **Qual** | **(V)** |
| Trimethylphenanthrene, 1,2,6- | PHE-1,2,6 | 220.3 | 5.99 | 220-205 | 20 | 205-189 | 35 |
| Trimethylphenanthrene, 1,2,7- | PHE-1,2,7 | 220.3 | 5.99 | 220-205 | 20 | 205-189 | 35 |
| Tetramethylphenanthrene, 1,2,6,9- | PHE-1,2,6,9 | 234.3 | 6.53 | 234-219 | 20 | 234-203 | 35 |
| Fluoranthene | FLA | 202.3 | 4.93 | 202-200 | 40 | 202-201 | 25 |
| Pyrene | PYR | 202.1 | 4.93 | 202-200 | 45 | 202-201 | 25 |
| Methylfluoranthene, 2- | FLA-2 | 216.3 | 5.48 | 216-215 | 30 | 215-213 | 40 |
| Methylpyrene, 1- | PYR-1 | 216.3 | 5.48 | 216-215 | 30 | 215-189 | 30 |
| Dimethylpyrene, 4,5- | PYR-4,5 | 230.3 | 6.03 | 230-215 | 30 | 215-189 | 30 |
| Propylpyrene, 1-n- | PYR-1pro | 244.3 | 6.46 | 215-189 | 40 | 230-215 | 30 |
| Ethylpyrene, 1- | PYR-1et | 230.3 | 5.97 | 215-189 | 35 | 215-213 | 45 |
| Butylpyrene, 1-n- | PYR-1but | 258.3 | 6.95 | 215-189 | 40 | 215-213 | 40 |
| Benz[a]anthracene | BAA | 228.3 | 5.52 | 228-226 | 45 | 226-224 | 45 |
| Chrysene | CHR | 228.3 | 5.52 | 228-226 | 45 | 228-227 | 25 |
| Methylchrysene, 1- | CHR-1 | 242.3 | 6.07 | 242-241 | 20 | 242-239 | 40 |
| Ethylchrysene, 6- | CHR-6et | 256.3 | 6.56 | 256-241 | 15 | 256-239 | 45 |
| Propylchrysene, 6-n- | CHR-6pro | 270.4 | 7.05 | 241-239 | 35 | 270-241 | 20 |
| Butylchrysene, 6-n- | CHR-6but | 284.4 | 7.54 | 241-239 | 40 | 284-241 | 25 |
| Benzo[b]fluoranthene | BBF | 252.3 | 6,11 | 252-250 | 45 | 250-248 | 45 |
| Benzo[k]fluoranthene | BKF | 252.3 | 6,11 | 252-250 | 45 | 250-248 | 45 |
| Benzo[e]pyrene | BEP | 252.3 | 6,11 | 252-250 | 45 | 250-248 | 45 |
| Benzo[a]pyrene | BAP | 252.3 | 6,11 | 252-250 | 45 | 250-248 | 45 |
| Perylene | PER | 252.3 | 6,11 | 252-250 | 45 | 250-248 | 45 |
| Indeno[1,2,3-cd]pyrene | IND | 276.3 | 6.70 | 276-274 | 45 | 274-272 | 45 |
| Dibenz[a,h]anthracene | DBA | 278.3 | 6.70 | 278-276 | 45 | 276-274 | 45 |
| Benzo[g,h,i]perylene | BGP | 276.3 | 6.70 | 276-274 | 45 | 274-272 | 45 |
| Naphthalene-*d*8 |  | 136.2 |  | 136-108 | 25 | 136-131 | 25 |
| Biphenyl-*d*10 |  | 164.3 |  | 164-122 | 40 | 164-131 | 30 |
| Acenapthylene-*d*8 |  | 160.2 |  | 160-158 | 30 | 160-132 | 30 |
| Anthracene-*d*10 |  | 188.3 |  | 188-161 | 35 | 188-181 | 40 |
| Pyrene-*d*10 |  | 212.3 |  | 212-208 | 40 | 212-210 | 35 |
| Perylene-*d*12 |  | 264.4 |  | 264-262 | 40 |  |  |
| Indeno[1,2,3,*cd*]pyrene-*d*12 |  | 288.4 |  | 288-282 | 50 | 288-286 | 40 |

S1 Table continued

| **Analyte** | **Short** | **Mw (g/mol)** | **log Kow** | **MRM**  **Quant** | **CE**  **(V)** | **MRM**  **Qual** | **CE**  **(V)** |
| --- | --- | --- | --- | --- | --- | --- | --- |
| C1-benzothiophenes | C1-BT | 148.3 | 3.54 | 148-133  148-147 | 15  15 |  |  |
| C2- benzothiophenes | C2-BT | 162.3 | 4.08 | 162-147  162-161 | 15  15 |  |  |
| C3- benzothiophenes | C3-BT | 176.3 | 4,63 | 176-161  176-175 | 15  20 |  |  |
| C4- benzothiophenes | C4-BT | 190.4 | 5.18 | 190-161  190-175 | 15  15 |  |  |
| C2-naphthalenes | C2-NAP | 156.2 | 4.26 | 156-141 | 20 |  |  |
| C3- naphthalenes | C3-NAP | 170.3 | 4.81 | 170-141  170-155 | 20  15 |  |  |
| C4- naphthalenes | C4-NAP | 184.3 | 4.96 | 184-141  184-155  184-169 | 20  20  20 |  |  |
| C1-fluorenes | C1-FLU | 180.3 | 4.97 | 180-179 | 30  30 |  |  |
| C2- fluorenes | C2-FLU | 194.3 | 5.11 | 194-165  194-179 | 30  30 |  |  |
| C3- fluorenes | C3-FLU | 208.3 | 5.66 | 208-179  208-193 | 30  30 |  |  |
| C1- fluorenes | C1-DBT | 198.3 | 4.71 | 206-191 | 20 |  |  |
| C2- fluorenes | C2-DBT | 212.3 | 5.26 | 220-191  220-205 | 25  20 |  |  |
| C3- fluorenes | C3-DBT | 226.4 | 5.81 | 234-191  234-205  234-219 | 25  25  20 |  |  |
| C4- fluorenes | C4-DBT | 240.4 | 6.35 | 198-197 | 20 |  |  |
| C2-phenanthrenes | C2-PHE | 206.3 | 5.44 | 212-197  212-211 | 20  20 |  |  |
| C3- phenanthrenes | C3-PHE | 220.3 | 5.99 | 226-197  226-211 | 20  20 |  |  |
| C4- phenanthrenes | C4-PHE | 234.3 | 6.53 | 240-197  240-211  240-225 | 30  25  20 |  |  |
| C1-pyrenes | C1-PYR | 216.3 | 5.48 | 216-215 | 30 |  |  |
| C2- pyrenes | C2-PYR | 230.3 | 6.03 | 230-215 | 30 |  |  |
| C3- pyrenes | C3-PYR | 244.3 | 6.57 | 244-215  244-229 | 30  30 |  |  |
| C1-chrysenes | C1-CHR | 242.3 | 6.07 | 242-241 | 20 |  |  |
| C2- chrysenes | C2-CHR | 256.3 | 6.62 | 256-241 | 15 |  |  |
| C3- chrysenes | C3-CHR | 270.4 | 7.16 | 270-241  270-255 | 20  20 |  |  |
| C4- chrysenes | C4-CHR | 284.4 | 7.71 | 284-241  284-255  284-269 | 20  20  20 |  |  |

1. U.S. EPA. Estimation Programs Interface Suite™ for Microsoft® Windows v 4.11. United States Environmental Protection Agency

Washington, DC, USA2012.
