# Supplementary material for: Oil droplet fouling and differential toxicokinetics of polycyclic aromatic hydrocarbons in embryos of Atlantic haddock and cod
Source: PLoS One. 2017 Jul 5;12(7):e0180048. doi: 10.1371/journal.pone.0180048 (PMC5497984; doi:10.1371/journal.pone.0180048)
Supplement: S2 Table — (DOC) [file pone.0180048.s012.doc]

**sdfS2 Table. Real time qPCR primer and probe sequences for *cyp1a*.**

| **Gene** | **Forward primer 5`-3`** | **Reverse primer 5`-3`** | **Probe 5`-3`** | **Quencher** |
| --- | --- | --- | --- | --- |
|  |  |  |  |  |
| *cyp1a* | CCTCCTTCCTGCCCTTCAC | TTGGGAATGAAGTAGCCATTGA | 6FAM-CCTCACTGCGCCACAAAAGACACATC | *Tamra* |
| *ef1α* | ATCGGCGGTATCGGAACAG | GCTTGAGGACACCGGTCTCA | 6FAM-ACCCGTGGGCCGTG | none |
