# Supplementary material for: Oil droplet fouling and differential toxicokinetics of polycyclic aromatic hydrocarbons in embryos of Atlantic haddock and cod
Source: PLoS One. 2017 Jul 5;12(7):e0180048. doi: 10.1371/journal.pone.0180048 (PMC5497984; doi:10.1371/journal.pone.0180048)
Supplement: S3 Table — Concentration of individual and alkyl cluster PAHs measured in water samples (ng/L) of the individual exposure groups, and in the crude oil (μg/g) used in the experiments, given with one standard deviation (n = 3). LOQ = limit of quantification. (DOC) [file pone.0180048.s013.doc]

**S3 Table. Measured PAH exposure concentrations.** Concentration of individual and alkyl cluster PAHs measured in water samples (ng/L) of the individual exposure groups, and in the crude oil (µg/g) used in the experiments, given with one standard deviation (n=3). LOQ=limit of quantification.

| **Exposure (ng/L)** | **Cod**  **Control** | **Cod**  **0.15 µg/L** | **Cod**  **0.29 µg/L** | **Cod**  **2.9 µg/L** | **Cod**  **3.6 µg/L** | **Cod**  **9.1 µg/L** | **Haddock**  **Control 1** | **Haddock**  **0.09 µg/L** | **Haddock**  **0.21 µg/L** |
| --- | --- | --- | --- | --- | --- | --- | --- | --- | --- |
| BIP | 1.1 ± 0.2 | 2.57 ± 0.19 | 5.04 ± 0.77 | 50 ± 10 | 61 ± 8 | 158 ± 16 | 0.5 ± 0.2 | 0.6 ± 0.1 | 3.8 ± 0.6 |
| BT | <LOQ | <LOQ | <LOQ | <LOQ | <LOQ | <LOQ | <LOQ | <LOQ | <LOQ |
| BT-2,5 | 0.07 ± 0.01 | 0.13 ± 0.01 | 0.23 ± 0.03 | 2 ± 0.4 | 2.6 ± 0.3 | 6.6 ± 0.6 | 0.072 ± 0.007 | 0.067 ± 0.003 | 0.17 ± 0.01 |
| BT-2,5,7 | <LOQ | 0.37 ± 0.02 | 0.42 ± 0.02 | 3.1 ± 0.3 | 5.5 ± 0.3 | 11.5 ± 0.7 | <LOQ | <LOQ | 0.39 ± 0.03 |
| NAP | 5 ± 5 | 8 ± 3 | 14 ± 2 | 135 ± 15 | 172 ± 12 | 426 ± 12 | 1.4 ± 0.3 | 1.4 ± 0.2 | 9.5 ± 0.8 |
| NAP-2 | 3.1 ± 0.6 | 13.8 ± 0.3 | 29 ± 3 | 297 ± 53 | 348 ± 41 | 905 ± 57 | 1.8 ± 0.5 | 1.9 ± 0.3 | 20 ± 2 |
| NAP-1 | 4 ± 5 | 5.9 ± 0.6 | 13 ± 1 | 123 ± 26 | 146 ± 17 | 377 ± 28 | 0.8 ± 0.3 | 1 ± 0.3 | 8 ± 1 |
| NAP-2,6&2,7 | 3 ± 2 | 8 ± 0.8 | 17 ± 3 | 149 ± 31 | 185 ± 27 | 489 ± 56 | 1 ± 0.2 | 1 ± 0.2 | 11 ± 2 |
| NAP-1,4 | 3 ± 2 | 6.4 ± 0.8 | 14 ± 3 | 123 ± 26 | 154 ± 18 | 405 ± 47 | 0.6 ± 0.3 | 0.7 ± 0.2 | 9 ± 2 |
| NAP-1,3&2,3 | 2 ± 2 | 3.3 ± 0.5 | 7 ± 2 | 64 ± 13 | 80 ± 11 | 213 ± 24 | 0.2 ± 0.1 | 0.2 ± 0.1 | 5 ± 0.8 |
| NAP-1,3,7 | <LOQ | 1.1 ± 0.3 | 4 ± 1 | 36 ± 8 | 45 ± 11 | 123 ± 18 | <LOQ | <LOQ | 2.2 ± 0.7 |
| NAP-2,3,5 | 1 ± 2 | 5 ± 1 | 12 ± 3 | 110 ± 24 | 140 ± 30 | 358 ± 55 | <LOQ | <LOQ | 7 ± 2 |
| NAP-1,2,3 | <LOQ | <LOQ | <LOQ | 3.7 ± 0.9 | 4 ± 1 | 12 ± 2 | <LOQ | <LOQ | <LOQ |
| NAP-1,2,5,6 | <LOQ | 0.06 ± 0.05 | 0.5 ± 0.2 | 9 ± 2 | 11 ± 2 | 29 ± 6 | <LOQ | <LOQ | 0.2 ± 0.2 |
| NAP-1,4,6,7 | <LOQ | 0.18 ± 0.07 | 0.7 ± 0.3 | 12 ± 3 | 14 ± 3 | 38 ± 9 | <LOQ | <LOQ | 0.4 ± 0.2 |
| ACY | 0.1 ± 0.1 | 0.011 ± 0.009 | 0.02 ± 0.02 | 0.17 ± 0.05 | 0.26 ± 0.06 | 0.5 ± 0.1 | 0.02 ± 0.02 | 0.02 ± 0.01 | 0.006 ± 0.009 |
| ACE | 0.32 ± 0.08 | 0.43 ± 0.04 | 0.7 ± 0.1 | 4.6 ± 0.9 | 5.8 ± 0.7 | 15 ± 2 | 0.23 ± 0.03 | 0.21 ± 0.01 | 0.55 ± 0.02 |
| DBF | 0.51 ± 0.03 | 1 ± 0.06 | 1.8 ± 0.2 | 14 ± 2 | 18 ± 2 | 46 ± 5 | 0.34 ± 0.03 | 0.35 ± 0.02 | 1.3 ± 0.1 |
| FLU | 2 ± 2 | 2.1 ± 0.2 | 3.8 ± 0.4 | 32 ± 6 | 41 ± 4 | 108 ± 19 | 0.8 ± 0.1 | 0.7 ± 0.2 | 2.9 ± 0.3 |
| FLU-9et | <LOQ | <LOQ | <LOQ | 0.9 ± 0.2 | 1.1 ± 0.2 | 2.9 ± 0.5 | <LOQ | <LOQ | <LOQ |
| FLU-1 | <LOQ | 1.3 ± 0.2 | 2.9 ± 0.6 | 33 ± 6 | 42 ± 7 | 110 ± 22 | 0.03 ± 0.06 | 0.02 ± 0.04 | 2 ± 0.4 |
| FLU-9pro | <LOQ | <LOQ | <LOQ | <LOQ | <LOQ | <LOQ | <LOQ | <LOQ | <LOQ |
| DBT | 2 ± 2 | 1.2 ± 0.1 | 1.9 ± 0.3 | 17 ± 3 | 21 ± 2 | 56 ± 12 | 0.68 ± 0.03 | 0.6 ± 0.2 | 1.5 ± 0.2 |
| DBT-4 | 0.04 ± 0.07 | 0.4 ± 0.2 | 1.1 ± 0.4 | 12 ± 3 | 15 ± 2 | 39 ± 7 | <LOQ | 0.1 ± 0.2 | 0.7 ± 0.2 |
| DBT-4et | <LOQ | <LOQ | 0.02 ± 0.03 | 4 ± 1 | 4.7 ± 0.6 | 13 ± 3 | <LOQ | <LOQ | <LOQ |
| DBT-4pro | 0.04 ± 0.08 | 0.02 ± 0.04 | 0.18 ± 0.07 | 4 ± 2 | 3.1 ± 0.7 | 11 ± 3 | <LOQ | 0.1 ± 0.3 | 0.07 ± 0.04 |
| DBT-4but | 0.2 ± 0.3 | 0.26 ± 0.05 | 0.36 ± 0.02 | 4 ± 1 | 3.7 ± 0.4 | 12 ± 3 | <LOQ | 0.4 ± 0.6 | 0.28 ± 0.02 |
| PHE | 2 ± 2 | 1.9 ± 0.3 | 4.1 ± 0.6 | 33 ± 6 | 41 ± 6 | 105 ± 26 | 0.2 ± 0.2 | 0.4 ± 0.6 | 2.7 ± 0.4 |
| ANT | <LOQ | <LOQ | <LOQ | <LOQ | <LOQ | <LOQ | <LOQ | <LOQ | <LOQ |
| PHE-3 | 0.3 ± 0.4 | 1.2 ± 0.2 | 2.3 ± 0.4 | 26 ± 7 | 30 ± 3 | 81 ± 15 | 0.26 ± 0.07 | 0.5 ± 0.4 | 1.6 ± 0.2 |
| PHE-2 | 1 ± 2 | 0.6 ± 0.1 | 1.6 ± 0.4 | 21 ± 6 | 24 ± 3 | 65 ± 12 | <LOQ | 0.1 ± 0.2 | 1 ± 0.2 |
| PHE-9 | 0.5 ± 0.5 | 1.6 ± 0.3 | 3 ± 0.5 | 32 ± 8 | 37 ± 4 | 94 ± 14 | 0.2 ± 0.1 | 0.6 ± 0.7 | 2.2 ± 0.3 |
| PHE-1 | 1 ± 1 | 1 ± 0.2 | 1.9 ± 0.4 | 21 ± 6 | 23 ± 3 | 63 ± 14 | 0.02 ± 0.04 | 0.2 ± 0.4 | 1.4 ± 0.2 |
| PHE-3,6 | 2 ± 2 | 0.5 ± 0.2 | 1 ± 0.2 | 12 ± 4 | 12 ± 2 | 36 ± 9 | 0.08 ± 0.05 | 0.3 ± 0.5 | 0.7 ± 0.06 |
| PHE-1,7 | 0.5 ± 0.5 | 0.8 ± 0.2 | 1.5 ± 0.2 | 15 ± 5 | 14 ± 2 | 43 ± 10 | 0.16 ± 0.03 | 0.8 ± 1.1 | 1.1 ± 0.1 |
| PHE-1,2 | 0.1 ± 0.1 | 0.19 ± 0.02 | 0.28 ± 0.04 | 2.4 ± 0.6 | 2.7 ± 0.4 | 9 ± 2 | <LOQ | 0.1 ± 0.2 | 0.23 ± 0.03 |
| PHE-2,6,9 | 0.1 ± 0.2 | <LOQ | 1 ± 0.4 | 19 ± 7 | 23 ± 2 | 63 ± 15 | <LOQ | 0.5 ± 0.9 | 0.31 ± 0.01 |
| PHE-1,2,6 | <LOQ | <LOQ | <LOQ | 3 ± 1 | 4.4 ± 0.6 | 10 ± 2 | <LOQ | <LOQ | <LOQ |
| PHE-1,2,7 | <LOQ | <LOQ | <LOQ | 1.1 ± 0.4 | 1.5 ± 0.2 | 3.5 ± 0.5 | <LOQ | <LOQ | <LOQ |
| PHE-1,2,6,9 | <LOQ | <LOQ | <LOQ | 1.1 ± 0.9 | 1.7 ± 0.2 | 3 ± 2 | <LOQ | <LOQ | <LOQ |
| FLA | 2 ± 2 | 0.4 ± 0.2 | 0.5 ± 0.09 | 2.3 ± 0.9 | 1.3 ± 0.4 | 5 ± 2 | 0.24 ± 0.05 | 1 ± 1 | 0.32 ± 0.06 |
| PYR | 3 ± 3 | 0.5 ± 0.7 | 0.5 ± 0.4 | 2 ± 1 | 1.7 ± 0.6 | 6 ± 2 | 0.2 ± 0.2 | 7 ± 12 | 0.2 ± 0.1 |
| FLA-2 | <LOQ | <LOQ | <LOQ | 1 ± 0.4 | 0.9 ± 0.2 | 3 ± 1 | <LOQ | <LOQ | <LOQ |
| PYR-1 | <LOQ | <LOQ | <LOQ | 1.5 ± 0.9 | 0.02 ± 0.03 | 2 ± 1 | <LOQ | 0.04 ± 0.07 | <LOQ |
| PYR-4,5 | <LOQ | <LOQ | <LOQ | <LOQ | <LOQ | <LOQ | <LOQ | <LOQ | <LOQ |
| PYR-1pro | <LOQ | <LOQ | <LOQ | <LOQ | <LOQ | <LOQ | <LOQ | <LOQ | <LOQ |
| PYR-1et | <LOQ | <LOQ | <LOQ | <LOQ | <LOQ | <LOQ | <LOQ | <LOQ | <LOQ |
| PYR-1but | <LOQ | <LOQ | <LOQ | <LOQ | <LOQ | <LOQ | <LOQ | <LOQ | <LOQ |
| BAA | 1 ± 2 | <LOQ | <LOQ | <LOQ | <LOQ | <LOQ | <LOQ | <LOQ | <LOQ |
| CHR | 1 ± 1 | 0.11 ± 0.02 | 0.41 ± 0.04 | 4 ± 1 | 5.6 ± 0.5 | 14 ± 3 | <LOQ | 0.1 ± 0.2 | 0.21 ± 0.03 |
| CHR-1 | <LOQ | <LOQ | <LOQ | 3.5 ± 0.2 | 6.4 ± 0.1 | 13.6 ± 0.5 | <LOQ | <LOQ | <LOQ |
| CHR-6et | <LOQ | <LOQ | <LOQ | <LOQ | <LOQ | <LOQ | <LOQ | <LOQ | <LOQ |
| CHR-6pro | <LOQ | <LOQ | <LOQ | <LOQ | <LOQ | <LOQ | <LOQ | <LOQ | <LOQ |
| CHR-6but | <LOQ | <LOQ | <LOQ | <LOQ | <LOQ | <LOQ | <LOQ | <LOQ | <LOQ |
| BBF | <LOQ | 0.19 ± 0.01 | 0.29 ± 0.03 | 2.1 ± 0.4 | 3.2 ± 0.1 | 7.3 ± 0.6 | <LOQ | 0.15 ± 0.03 | 0.21 ± 0.02 |
| BKF | 2 ± 4 | 0.2 ± 0.3 | <LOQ | <LOQ | <LOQ | <LOQ | <LOQ | <LOQ | <LOQ |
| BEP | 1 ± 2 | 0.07 ± 0.03 | 0.17 ± 0.02 | 1.5 ± 0.4 | 1.9 ± 0.2 | 5.1 ± 0.9 | <LOQ | 0.1 ± 0.2 | 0.11 ± 0.02 |
| BAP | <LOQ | <LOQ | <LOQ | 1.8 ± 0.6 | 0.8 ± 0.5 | 5 ± 1 | <LOQ | <LOQ | <LOQ |
| PER | 1 ± 2 | <LOQ | <LOQ | 1.15 ± 0.09 | 2.11 ± 0.09 | 4.5 ± 0.2 | <LOQ | <LOQ | <LOQ |
| IND | <LOQ | <LOQ | <LOQ | 0.1 ± 0.2 | <LOQ | 0.1 ± 0.1 | <LOQ | <LOQ | <LOQ |
| DBA | 3 ± 4 | 0.42 ± 0.04 | 0.417 ± 0.008 | 6.3 ± 0.2 | 12.1 ± 0.3 | 24.3 ± 0.9 | 0.47 ± 0.08 | 0.41 ± 0.03 | <LOQ |
| BGP | <LOQ | <LOQ | <LOQ | 1.1 ± 0.3 | 1.58 ± 0.06 | 3.7 ± 0.5 | <LOQ | <LOQ | <LOQ |
| C1-NAP | 11 ± 8 | 21 ± 3 | 41 ± 6 | 422 ± 63 | 455 ± 255 | 1373 ± 175 | 3 ± 2 | 3.7 ± 0.6 | 31 ± 4 |
| C2-NAP | 14 ± 18 | 35 ± 4 | 58 ± 11 | 552 ± 89 | 703 ± 171 | 1859 ± 354 | <LOQ | 5.2 ± 0.8 | 49 ± 7 |
| C3-NAP | 8 ± 7 | 29 ± 1 | 56 ± 8 | 508 ± 110 | 728 ± 77 | 1631 ± 240 | <LOQ | 5.7 ± 0.8 | 41 ± 2 |
| C4-NAP | <LOQ | 20 ± 3 | 37 ± 4 | 300 ± 51 | 447 ± 70 | 927 ± 131 | <LOQ | 10 ± 3 | 29 ± 6 |
| C1-PHE | 3 ± 3 | 5 ± 1 | 10 ± 1 | 106 ± 26 | 135 ± 18 | 329 ± 64 | <LOQ | 2 ± 3 | 7 ± 1 |
| C2-PHE | 3 ± 5 | 7 ± 1 | 15 ± 1 | 161 ± 35 | 186 ± 32 | 475 ± 103 | <LOQ | 5 ± 9 | 10 ± 1 |
| C3-PHE | 33 ± 56 | <LOQ | 9.5 ± 0.7 | 103 ± 34 | 121 ± 10 | 330 ± 72 | <LOQ | 10 ± 18 | 5 ± 4 |
| C4-PHE | <LOQ | <LOQ | 7 ± 1 | 67 ± 21 | 79 ± 10 | 216 ± 41 | <LOQ | 6 ± 10 | 2 ± 3 |
| C1-DBT | 1 ± 2 | 3 ± 1 | 5 ± 1 | 54 ± 15 | 66 ± 8 | 168 ± 36 | <LOQ | 1 ± 2 | 3 ± 1 |
| C2-DBT | 5 ± 8 | 9 ± 4 | 14 ± 2 | 127 ± 30 | 153 ± 16 | 405 ± 76 | <LOQ | 9 ± 15 | 10 ± 2 |
| C3-DBT | 8 ± 14 | 9 ± 5 | 14 ± 1 | 131 ± 37 | 163 ± 22 | 427 ± 85 | <LOQ | 20 ± 35 | 9 ± 2 |
| C1-CHR | <LOQ | <LOQ | <LOQ | 11 ± 3 | 13 ± 2 | 38 ± 9 | <LOQ | 0.3 ± 0.5 | <LOQ |
| C2-CHR | <LOQ | <LOQ | <LOQ | 33 ± 8 | 36 ± 4 | 102 ± 18 | <LOQ | <LOQ | <LOQ |
| C3-CHR | <LOQ | <LOQ | <LOQ | 28 ± 8 | 33 ± 5 | 92 ± 18 | <LOQ | <LOQ | <LOQ |
| Total PAH (ng/L) | 113 ± 92 | 154 ± 12 | 290 ± 31 | 2845 ± 522 | 3591 ± 613 | 9143 ± 1389 | 5 ± 3 | 88 ± 109 | 211 ± 27 |
| THC (µg/L) | 23 ± 4 | 31 ± 13 | 34 ± 14 | 130 ± 17 | 150 ± 22 | 366 ± 59 | 24 ± 2 | 39 ± 34 | 23 ± 2 |

**Table S3 continued.**

| **S3 Table**  **Exposure (ng/L)** | **Haddock**  **8.6 µg/L** | **Haddock**  **Control 2** | **Haddock**  **0.10 µg/L** | **Haddock**  **0.17 µg/L** | **Haddock**  **0.76 µg/L** | **Haddock**  **2.7 µg/L** | **Haddock**  **3.5 µg/L** | **Haddock**  **WSF** | **Heidrun crude**  **oil (µg/g)** |
| --- | --- | --- | --- | --- | --- | --- | --- | --- | --- |
| BIP | 149 ± 17 | 1.1 ± 0.8 | 0.9 ± 0.2 | 1.6 ± 0.2 | 13 ± 1 | 42 ± 2 | 50 ± 1 | 39.7 ± 0.6 | 350 ± 4 |
| BT | <LOQ | <LOQ | <LOQ | <LOQ | <LOQ | <LOQ | <LOQ | <LOQ | <LOQ |
| BT-2,5 | 6.6 ± 0.7 | 0.08 ± 0.03 | 0.09 ± 0.01 | 0.11 ± 0.02 | 0.63 ± 0.05 | 1.8 ± 0.1 | 2.1 ± 0.1 | 1.6 ± 0.2 | 16 ± 1 |
| BT-2,5,7 | 11.5 ± 0.4 | <LOQ | 0.2 ± 0.2 | 0.34 ± 0.02 | 0.98 ± 0.04 | 2.5 ± 0.1 | 2.7 ± 0.2 | 2 ± 0.2 | 10.1 ± 0.3 |
| NAP | 396 ± 23 | 3 ± 3 | 2.4 ± 0.4 | 3 ± 1 | 31 ± 6 | 100 ± 10 | 125 ± 12 | 106 ± 7 | 841 ± 44 |
| NAP-2 | 850 ± 99 | 4 ± 4 | 5 ± 1 | 7 ± 2 | 66 ± 10 | 220 ± 8 | 257 ± 8 | 206 ± 12 | 1994 ± 36 |
| NAP-1 | 353 ± 40 | 2 ± 2 | 1.3 ± 0.5 | 2.7 ± 0.8 | 28 ± 6 | 104 ± 5 | 119 ± 11 | 94 ± 5 | 1002 ± 147 |
| NAP-2,6&2,7 | 479 ± 56 | 2 ± 2 | 3 ± 1 | 6 ± 2 | 50 ± 6 | 155 ± 8 | 184 ± 10 | 133 ± 18 | 1436 ± 38 |
| NAP-1,4 | 397 ± 49 | 2 ± 2 | 2.4 ± 0.8 | 4 ± 1 | 39 ± 5 | 127 ± 7 | 152 ± 8 | 124 ± 15 | 1176 ± 59 |
| NAP-1,3&2,3 | 209 ± 26 | 0.7 ± 0.8 | 1.1 ± 0.5 | 2 ± 0.7 | 21 ± 3 | 73 ± 4 | 87 ± 5 | 60 ± 6 | 530 ± 15 |
| NAP-1,3,7 | 120 ± 17 | <LOQ | 0.1 ± 0.2 | 0.6 ± 0.4 | 10 ± 1 | 32 ± 2 | 38 ± 4 | 28 ± 9 | 338 ± 3 |
| NAP-2,3,5 | 365 ± 37 | 0.2 ± 0.3 | 0.7 ± 0.9 | 2 ± 1 | 32 ± 4 | 106 ± 7 | 126 ± 15 | 84 ± 20 | 495 ± 9 |
| NAP-1,2,3 | 12 ± 2 | <LOQ | <LOQ | <LOQ | 0.1 ± 0.2 | 1.6 ± 0.3 | 2.5 ± 0.8 | 1 ± 0.3 | 37 ± 1 |
| NAP-1,2,5,6 | 28 ± 5 | <LOQ | <LOQ | <LOQ | 1.7 ± 0.3 | 8 ± 3 | 10 ± 4 | 4 ± 1 | 83 ± 6 |
| NAP-1,4,6,7 | 38 ± 9 | <LOQ | <LOQ | 0.04 ± 0.08 | 2.3 ± 0.5 | 11 ± 3 | 14 ± 5 | 5 ± 1 | 99 ± 5 |
| ACY | 0.6 ± 0.1 | <LOQ | <LOQ | <LOQ | 0.02 ± 0.02 | 0.05 ± 0.05 | 0.04 ± 0.04 | 0.06 ± 0.08 | 41 ± 4 |
| ACE | 16 ± 2 | 0.22 ± 0.06 | 0.26 ± 0.04 | 0.36 ± 0.05 | 1.6 ± 0.2 | 4.9 ± 0.4 | 6 ± 0.7 | 3.9 ± 0.4 | <LOQ |
| DBF | 47 ± 6 | 0.4 ± 0.2 | 0.44 ± 0.09 | 0.6 ± 0.1 | 4.8 ± 0.2 | 14 ± 1 | 17 ± 2 | 12.6 ± 0.9 | 107 ± 15 |
| FLU | 108 ± 15 | 0.6 ± 0.3 | 0.9 ± 0.2 | 1.2 ± 0.3 | 9.6 ± 0.9 | 32 ± 2 | 40 ± 4 | 26 ± 1 | 268 ± 16 |
| FLU-9et | 2.9 ± 0.4 | <LOQ | <LOQ | <LOQ | <LOQ | <LOQ | <LOQ | <LOQ | 8 ± 1 |
| FLU-1 | 113 ± 14 | 0.02 ± 0.04 | 0.3 ± 0.2 | 0.7 ± 0.3 | 8.2 ± 0.5 | 26 ± 2 | 33 ± 4 | 20 ± 3 | 245 ± 27 |
| FLU-9pro | <LOQ | <LOQ | <LOQ | <LOQ | <LOQ | <LOQ | <LOQ | <LOQ | 2.78 ± 0.02 |
| DBT | 57 ± 13 | 0.47 ± 0.06 | 0.61 ± 0.07 | 0.7 ± 0.2 | 5 ± 0.4 | 16.6 ± 0.9 | 21 ± 3 | 11.95 ± 0.75 | 128 ± 30 |
| DBT-4 | 40 ± 6 | <LOQ | <LOQ | 0.1 ± 0.1 | 3.4 ± 0.5 | 13 ± 2 | 16 ± 5 | 9 ± 2 | 110 ± 7 |
| DBT-4et | 13 ± 4 | <LOQ | <LOQ | <LOQ | 0.2 ± 0.2 | 3 ± 2 | 5 ± 4 | 0.1 ± 0.1 | 39 ± 3 |
| DBT-4pro | 10 ± 6 | <LOQ | <LOQ | <LOQ | 0.6 ± 0.2 | 4 ± 3 | 6 ± 4 | 0.02 ± 0.03 | 32 ± 1 |
| DBT-4but | 11 ± 4 | <LOQ | 0.2 ± 0.03 | 0.22 ± 0.06 | 0.9 ± 0.1 | 4 ± 2 | 5 ± 3 | 0.8 ± 0.1 | 20 ± 1 |
| PHE | 113 ± 20 | 0.01 ± 0.01 | 0.3 ± 0.2 | 0.7 ± 0.4 | 11 ± 0.8 | 32 ± 2 | 42 ± 6 | 29 ± 1 | 284 ± 57 |
| ANT | <LOQ | <LOQ | <LOQ | <LOQ | <LOQ | <LOQ | <LOQ | <LOQ | <LOQ |
| PHE-3 | 80 ± 15 | 0.21 ± 0.02 | 0.5 ± 0.2 | 0.8 ± 0.2 | 6.2 ± 0.5 | 21 ± 2 | 27 ± 6 | 13 ± 2 | 169 ± 2 |
| PHE-2 | 65 ± 12 | <LOQ | 0.04 ± 0.07 | 0.2 ± 0.2 | 4.5 ± 0.1 | 18 ± 3 | 24 ± 7 | 10 ± 2 | 146 ± 15 |
| PHE-9 | 88 ± 35 | 0.09 ± 0.04 | 0.6 ± 0.2 | 0.9 ± 0.4 | 8.3 ± 0.7 | 29 ± 4 | 38 ± 11 | 19 ± 3 | 232 ± 17 |
| PHE-1 | 64 ± 14 | <LOQ | 0.3 ± 0.2 | 0.5 ± 0.2 | 5.2 ± 0.2 | 19 ± 3 | 25 ± 8 | 12 ± 2 | 154 ± 13 |
| PHE-3,6 | 33 ± 10 | 0.024 ± 0.008 | 0.2 ± 0.1 | 0.3 ± 0.2 | 2.7 ± 0.3 | 11 ± 5 | 14 ± 8 | 4.1 ± 0.7 | 76 ± 6 |
| PHE-1,7 | 41 ± 15 | 0.08 ± 0.07 | 0.4 ± 0.1 | 0.5 ± 0.2 | 3.4 ± 0.1 | 15 ± 5 | 20 ± 13 | 4.6 ± 0.7 | 109 ± 7 |
| PHE-1,2 | 7 ± 2 | <LOQ | 0.13 ± 0.01 | 0.15 ± 0.03 | 0.68 ± 0.05 | 3 ± 1 | 3 ± 2 | 1.19 ± 0.09 | 15 ± 2 |
| PHE-2,6,9 | 58 ± 23 | <LOQ | <LOQ | <LOQ | 3 ± 2 | 17 ± 10 | 23 ± 16 | 1 ± 2 | 80 ± 17 |
| PHE-1,2,6 | 10 ± 4 | <LOQ | <LOQ | <LOQ | <LOQ | 0.5 ± 0.9 | 1 ± 2 | <LOQ | 15 ± 2 |
| PHE-1,2,7 | 3 ± 1 | <LOQ | <LOQ | <LOQ | <LOQ | <LOQ | 0.2 ± 0.4 | <LOQ | 5 ± 1 |
| PHE-1,2,6,9 | 3 ± 2 | <LOQ | <LOQ | <LOQ | <LOQ | <LOQ | <LOQ | <LOQ | 6 ± 1 |
| FLA | 5 ± 3 | 0.12 ± 0.02 | 0.14 ± 0.11 | 0.11 ± 0.05 | 0.75 ± 0.05 | 2.7 ± 0.7 | 4 ± 1 | 1.5 ± 0.2 | 20 ± 3 |
| PYR | 6 ± 2 | <LOQ | 0.3 ± 0.5 | 0.01 ± 0.02 | 0.7 ± 0.2 | 4 ± 2 | 5 ± 2 | 3 ± 2 | 25 ± 1 |
| FLA-2 | 3 ± 1 | <LOQ | <LOQ | <LOQ | <LOQ | <LOQ | <LOQ | <LOQ | 8.3 ± 0.4 |
| PYR-1 | 2 ± 3 | <LOQ | <LOQ | <LOQ | <LOQ | <LOQ | 0.3 ± 0.5 | <LOQ | 12 ± 1 |
| PYR-4,5 | <LOQ | <LOQ | <LOQ | <LOQ | <LOQ | <LOQ | <LOQ | <LOQ | <LOQ |
| PYR-1pro | 0.1 ± 0.2 | <LOQ | <LOQ | <LOQ | <LOQ | <LOQ | <LOQ | <LOQ | <LOQ |
| PYR-1et | <LOQ | <LOQ | <LOQ | <LOQ | <LOQ | <LOQ | <LOQ | <LOQ | 3 ± 0.2 |
| PYR-1but | <LOQ | <LOQ | <LOQ | <LOQ | <LOQ | <LOQ | <LOQ | <LOQ | 0.9 ± 0.1 |
| BAA | <LOQ | <LOQ | <LOQ | <LOQ | <LOQ | 0.1 ± 0.2 | 0.3 ± 0.5 | <LOQ | 3.8 ± 0.4 |
| CHR | 14 ± 4 | <LOQ | <LOQ | <LOQ | 0.9 ± 0.3 | 4 ± 2 | 5 ± 2 | 1.2 ± 0.4 | 23 ± 1 |
| CHR-1 | 13.4 ± 0.5 | <LOQ | <LOQ | <LOQ | <LOQ | 0.4 ± 0.6 | 1 ± 1 | <LOQ | 6.2 ± 0.3 |
| CHR-6et | <LOQ | <LOQ | <LOQ | <LOQ | <LOQ | <LOQ | <LOQ | <LOQ | <LOQ |
| CHR-6pro | <LOQ | <LOQ | <LOQ | <LOQ | <LOQ | <LOQ | <LOQ | <LOQ | <LOQ |
| CHR-6but | <LOQ | <LOQ | <LOQ | <LOQ | <LOQ | <LOQ | <LOQ | <LOQ | <LOQ |
| BBF | 8 ± 2 | 0.05 ± 0.08 | 0.14 ± 0.02 | 0.13 ± 0.04 | 0.7 ± 0.2 | 2 ± 1 | 3 ± 2 | 0.46 ± 0.01 | 10 ± 1 |
| BKF | <LOQ | <LOQ | <LOQ | <LOQ | <LOQ | 1.6 ± 0.4 | 1.8 ± 0.6 | 0.99 ± 0.03 | 2.6 ± 0.3 |
| BEP | 5 ± 2 | <LOQ | 0.03 ± 0.02 | 0.02 ± 0.03 | 0.5 ± 0.1 | 2 ± 1 | 3 ± 2 | 0.06 ± 0.05 | 13 ± 1 |
| BAP | 3 ± 4 | <LOQ | <LOQ | <LOQ | <LOQ | <LOQ | <LOQ | <LOQ | 3.3 ± 0.6 |
| PER | 4.4 ± 0.2 | <LOQ | <LOQ | <LOQ | <LOQ | <LOQ | <LOQ | <LOQ | 2.5 ± 0.1 |
| IND | 0.3 ± 0.4 | <LOQ | <LOQ | <LOQ | <LOQ | <LOQ | <LOQ | <LOQ | 1.24 ± 0.04 |
| DBA | 24.6 ± 0.9 | 0.46 ± 0.09 | 0.39 ± 0.02 | 0.43 ± 0.07 | 0.82 ± 0.03 | 1.9 ± 0.3 | 1.8 ± 0.3 | <LOQ | 2.3 ± 0.1 |
| BGP | 3.7 ± 0.6 | <LOQ | <LOQ | <LOQ | <LOQ | 0.9 ± 0.5 | 1.2 ± 0.8 | <LOQ | 5 ± 0.1 |
| C1-NAP | 1255 ± 138 | 8 ± 6 | 16 ± 4 | 24 ± 4 | 106 ± 12 | 334 ± 19 | 416 ± 13 | 305 ± 5 | 2418 ± 471 |
| C2-NAP | 1694 ± 189 | 8 ± 6 | 20 ± 6 | 32 ± 9 | 151 ± 16 | 508 ± 36 | 626 ± 32 | 411 ± 22 | 3405 ± 252 |
| C3-NAP | 1535 ± 191 | 3 ± 6 | 20 ± 8 | 32 ± 10 | 141 ± 17 | 482 ± 53 | 600 ± 82 | 329 ± 57 | 3491 ± 299 |
| C4-NAP | 884 ± 185 | <LOQ | 14 ± 5 | 20 ± 7 | 87 ± 11 | 296 ± 35 | 398 ± 67 | 157 ± 35 | 2143 ± 96 |
| C1-PHE | 324 ± 66 | <LOQ | 4 ± 1 | 6 ± 2 | 26 ± 3 | 92 ± 14 | 126 ± 30 | 59 ± 8 | 678 ± 35 |
| C2-PHE | 469 ± 123 | <LOQ | 6 ± 3 | 10 ± 4 | 39 ± 4 | 159 ± 55 | 219 ± 88 | 65 ± 6 | 1173 ± 31 |
| C3-PHE | 308 ± 98 | <LOQ | 3 ± 3 | 7 ± 4 | 29 ± 5 | 122 ± 60 | 169 ± 98 | 29 ± 1 | 690 ± 43 |
| C4-PHE | 210 ± 89 | <LOQ | <LOQ | 3 ± 5 | 20 ± 4 | 96 ± 56 | 129 ± 81 | <LOQ | 598 ± 66 |
| C1-DBT | 161 ± 32 | <LOQ | 2 ± 2 | 4 ± 1 | 14 ± 2 | 46 ± 5 | 70 ± 20 | 31 ± 3 | 168 ± 3 |
| C2-DBT | 378 ± 110 | <LOQ | 4 ± 4 | 8 ± 3 | 33 ± 5 | 123 ± 39 | 168 ± 70 | 53 ± 2 | 542 ± 21 |
| C3-DBT | 403 ± 149 | <LOQ | 4 ± 5 | 9 ± 5 | 37 ± 7 | 156 ± 81 | 213 ± 122 | 37 ± 7 | 572 ± 39 |
| C1-CHR | 35 ± 15 | <LOQ | <LOQ | <LOQ | 3 ± 0.5 | 15 ± 9 | 19 ± 13 | 1.73 ± 0.08 | 100 ± 8 |
| C2-CHR | 92 ± 39 | <LOQ | <LOQ | <LOQ | 8 ± 1 | 42 ± 25 | 55 ± 36 | <LOQ | 82 ± 5 |
| C3-CHR | 85 ± 38 | <LOQ | <LOQ | <LOQ | 9 ± 1 | 39 ± 23 | 51 ± 34 | <LOQ | 72 ± 10 |
| Total PAH (ng/L) | 8557 ± 1398 | 22 ± 19 | 101 ± 41 | 167 ± 56 | 760 ± 93 | 2694 ± 503 | 3497 ± 772 | 1643 ± 121 | 21394 ± 1970 |
| THC (µg/L) | 336 ± 121 | 11 ± 2 | 14 ± 1 | 17 ± 4 | 42 ± 6 | 139 ± 70 | 180 ± 101 | 28 ± 15 |  |
