# Supplementary material for: Oil droplet fouling and differential toxicokinetics of polycyclic aromatic hydrocarbons in embryos of Atlantic haddock and cod
Source: PLoS One. 2017 Jul 5;12(7):e0180048. doi: 10.1371/journal.pone.0180048 (PMC5497984; doi:10.1371/journal.pone.0180048)
Supplement: S4 Table — Relative response of alkanes (Σ(nC19-nC32)) normalized to the response of internal standard pyrene-d12. Given with one standard deviation (n = 3). (DOC) [file pone.0180048.s014.doc]

**Table S4. Alkane uptake.** Relative response of alkanes (Σ(nC19-nC32)) normalized to the response of internal standard pyrene-d12. Given with one standard deviation (n=3).

| **Exposure group** | **Day 3** | | | **Day 9** | | |
| --- | --- | --- | --- | --- | --- | --- |
| Haddock control | 0.77 | ± | 0.09 | 1.1 | ± | 0.3 |
| Haddock WSF | 0.9 | ± | 0.3 | 0.8 | ± | 0.2 |
| Haddock 0.10 µg/L | 1.0 | ± | 0.2 | 0.8 | ± | 0.2 |
| Haddock 0.21 µg/L | 0.8 | ± | 0.2 | 0.6 | ± | 0.3 |
| Haddock 0.76 µg/L | 1.0 | ± | 0.1 | 1.0 | ± | 0.2 |
| Haddock 2.7 µg/L | 1.0 | ± | 0.1 | 1.6 | ± | 0.2 |
| Haddock 8.6 µg/L | 1.5 | ± | 0.2 | 2.9 | ± | 0.3 |
| Cod control | 1.4 | ± | 0.8 | 1.0 | ± | 0.1 |
| Cod 0.15 µg/L | 1.2 | ± | 0.2 | 0.6 | ± | 0.1 |
| Cod 0.29 µg/L | 1.1 | ± | 0.1 | 0.84 | ± | 0.07 |
| Cod 2.9 µg/L | 0.90 | ± | 0.05 | 0.65 | ± | 0.03 |
| Cod 9.1 µg/L | 1.0 | ± | 0.1 | 0.88 | ± | 0.07 |
