# Supplementary material for: Oil droplet fouling and differential toxicokinetics of polycyclic aromatic hydrocarbons in embryos of Atlantic haddock and cod
Source: PLoS One. 2017 Jul 5;12(7):e0180048. doi: 10.1371/journal.pone.0180048 (PMC5497984; doi:10.1371/journal.pone.0180048)
Supplement: S5 Table — 1VFS, ventriclular fractional shortening; AFS, atrial fractional shortening; SV, silent ventricle. a–dLetters indicate significant differences between groups within the same experiment (p = <0.05) (groups with same letters are not significantly different from each other). *very underdeveloped ventricle treated as silent ventricle. (DOC) [file pone.0180048.s015.doc]

**Table S5. Characterization of cardiac function and morphology at 2 dph (cod) and 3 dph (haddock).** ^1^VFS, ventriclular fractional shortening; AFS, atrial fractional shortening; SV, silent ventricle. ^a–d^Letters indicate significant differences between groups within the same experiment (p=<0.05) (groups with same letters are not significantly different from each other). *very underdeveloped ventricle treated as silent ventricle.

|  | **Dose (µg/L tPAH)** | **AFS**^1^ | **VFS^1^** | **SV^1^ %** | **Oedema mm**^2^ | **Length (µm)** |
| --- | --- | --- | --- | --- | --- | --- |
| **Cod** | **Control** | 18 ± 4^a,b^ | 16 ± 6^a^ | 0 | 5 ± 5^a,b^ | 4652 ± 193^a^ |
|  | **0.15** | 17 ± 5^a^ | 17 ± 7^a,b^ | 0 | 7 ± 7^a,b^ | 4649 ± 192^a^ |
|  | **0.29** | 20 ± 4^a,b^ | 18 ± 5^a,b^ | 0 | 4 ± 2^a^ | 4480 ± 143^b^ |
|  | **2.8** | 19 ± 6^a,b^ | 20 ± 6^a,b^ | 0 | 8 ± 4^b,c^ | 4385 ± 191^b,c^ |
|  | **3.6** | 20 ± 5^a,b^ | 22 ± 5^b^ | 0 | 7 ± 3^a,b^ | 4265 ± 205^c^ |
|  | **9.1** | 21 ± 6^b^ | 18 ± 7^a,b^ | 3 | 12 ± 10^c,d^ | 4110 ± 201^d^ |
| **Haddock 1** | **Control** | 19 ± 5^a^ | 19 ± 5^a^ | 0 | 4 ± 3^a^ | 4486 ± 99 ^a^ |
|  | **0.09** | 22 ± 5^b^ | 22 ± 5^b^ | 2 | 5 ± 3^a,b^ | 4363 ± 567^a^ |
|  | **0.21** | 22 ± 4^b^ | 22 ± 4^b^ | 2 | 8 ± 8^b^ | 4489 ± 222^a^ |
|  | **8.6** | 11 ± 6^c^ | 11 ± 6^c^ | 97* | 27 ± 11^c^ | 3211 ± 233^b^ |
| **Haddock 2** | **Control** | 23 ± 7^a^ | 22 ± 3^a^ | 0 | 4 ± 2^a^ | 4564 ± 117^a^ |
|  | **0.10** | 17 ± 8^a^ | 14 ± 6^a^ | 0 | 8 ± 4^a^ | 4617 ± 151^a^ |
|  | **0.76** | 12 ± 7^a^ | 9 ± 10^a^ | 38 | 20 ± 8^b^ | 4037 ± 504^a^ |
|  | **2.7** | 19 ± 6^a^ | 8 ± 9^b^ | 35 | 35 ± 13^c^ | 3520 ± 410^a^ |
|  | **3.5** | 17 ± 7^a^ | 12 ± 11^a^ | 33 | 18 ± 7^b^ | 3740 ± 809^a^ |
|  | **WSF (1.6 µg/L)** | 24 ± 21^a^ | 16 ± 6^a^ | 0 | 18 ± 6^a^ | 4266 ± 180^b^ |
