# Supplementary material for: Oil droplet fouling and differential toxicokinetics of polycyclic aromatic hydrocarbons in embryos of Atlantic haddock and cod
Source: PLoS One. 2017 Jul 5;12(7):e0180048. doi: 10.1371/journal.pone.0180048 (PMC5497984; doi:10.1371/journal.pone.0180048)
Supplement: S6 Table — Linear regression performed on data sets of body burden (pg) embryo and Cyp1a fold change normalized to control in for both cod and haddock at day 3 and day 9. Correlation expressed in terms of the coefficient of determination (R2) for the regression. ND = not determined (too many missing body burden values). (DOC) [file pone.0180048.s016.doc]

**Table S6. Correlation between Cyp1a and PAH body burden.** Linear regression performed on data sets of body burden (pg) embryo and Cyp1a fold change normalized to control in for both cod and haddock at day 3 and day 9. Correlation expressed in terms of the coefficient of determination (R^2^) for the regression. ND=not determined (too many missing body burden values).

| **Analyte** | **Day 3** | **Day 9** | **Analyte** | **Day 3** | **Day 9** | **Analyte** | **Day 3** | **Day 9** | **Analyte** | **Day 3** | **Day 9** |
| --- | --- | --- | --- | --- | --- | --- | --- | --- | --- | --- | --- |
| Biphenyl | 0.011 | 0.039 | Methyldibenzothiophene, 4- | 0.597 | 0.880 | Ethylpyrene, 1- | ND | ND | C4-NAP | 0.731 | 0.907 |
| Benzothiophene | ND | ND | Ethyldibenzothiophene, 4- | 0.846 | 0.990 | Butylpyrene, 1-n- | ND | ND | C1-FLU | 0.319 | 0.787 |
| Dimethylbenzothiophene, 2,5- | 0.009 | 0.110 | Propyldibenzothiophene, 4-n-, | 0.966 | 0.994 | Benz[a]anthracene | ND | ND | C2-FLU | 0.712 | 0.968 |
| Trimethylbenzothiophene, 2,5,7- | 0.170 | 0.343 | Butyldibenzothiophene, 4-n- | 0.967 | 0.989 | Chrysene | 0.832 | 0.977 | C3-FLU | 0.898 | 0.994 |
| Naphthalene | 0.001 | 0.043 | Phenanthrene | 0.305 | 0.433 | Methylchrysene, 1- | 0.955 | 0.993 | C1-DBT | 0.451 | 0.871 |
| Methylnaphthalene, 2- | 0.002 | 0.017 | Anthracene | ND | ND | Ethylchrysene, 6- | ND | ND | C2-DBT | 0.797 | 0.993 |
| Methylnaphthalene, 1- | 0.003 | 0.014 | Methylphenanthrene, 3- | 0.409 | 0.903 | Propylchrysene, 6-n- | ND | ND | C3-DBT | 0.953 | 0.995 |
| Dimethylnaphthalene, 2,6&2,7- | 0.101 | 0.222 | Methylphenanthrene, 2- | 0.467 | 0.930 | Butylchrysene, 6-n- | ND | ND | C4-DBT | 0.967 | 0.991 |
| Dimethylnaphthalene, 1,4- | 0.119 | 0.194 | Methylphenanthrene, 9- | 0.523 | 0.848 | Benzo[b]fluoranthene | 0.981 | 0.991 | C1-PHE | 0.466 | 0.893 |
| Dimethylnaphthalene, 1,3&2,3- | 0.096 | 0.207 | Methylphenanthrene, 1- | 0.422 | 0.911 | Benzo[k]fluoranthene | 0.890 | ND | C2-PHE | 0.732 | 0.954 |
| Trimethylnaphthalene, 1,3,7- | 0.567 | 0.681 | Dimethylphenanthrene, 3,6- | 0.767 | 0.989 | Benzo[e]pyrene | 0.977 | 0.991 | C3-PHE | 0.934 | 0.993 |
| Trimethylnaphthalene, 2,3,5- | 0.529 | 0.608 | Dimethylphenanthrene, 1,7- | 0.739 | 0.993 | Benzo[a]pyrene | 0.997 | ND | C4-PHE | 0.963 | 0.993 |
| Trimethylnaphthalene, 1,2,3- | 0.432 | 0.527 | Dimethylphenanthrene, 1,2- | 0.692 | 0.993 | Perylene | 0.921 | ND | C1-PYR | 0.816 | 0.999 |
| Tetramethylnaphthalene, 1,2,5,6- | 0.786 | 0.936 | Trimethylphenanthrene, 2,6,9- | 0.883 | 0.956 | Indeno[1,2,3-cd]pyrene | ND | ND | C2-PYR | 0.935 | 0.995 |
| Tetramethylnaphthalene, 1,4,6,7- | 0.675 | 0.957 | Trimethylphenanthrene, 1,2,6- | 0.809 | 0.990 | Dibenz[a,h]anthracene | 0.735 | ND | C3-PYR | 0.952 | 0.992 |
| Acenaphthylene | 0.006 | 0.014 | Trimethylphenanthrene, 1,2,7- | 0.710 | 0.989 | Benzo[g,h,i]perylene | 0.943 | 0.987 | C1-CHR | 0.969 | 0.991 |
| Acenaphthene | 0.097 | 0.382 | Tetramethylphenanthrene, 1,2,6,9- | 0.961 | 0.992 | C1-BT | 0.057 | 0.096 | C2-CHR | 0.951 | 0.988 |
| Dibenzofuran | 0.008 | 0.109 | Fluoranthene | 0.515 | 0.938 | C2-BT | 0.001 | 0.020 | C3-CHR | 0.852 | 0.987 |
| Fluorene | 0.096 | 0.348 | Pyrene | 0.626 | 0.989 | C3-BT | 0.255 | 0.426 | C4-CHR | 0.933 | 0.985 |
| Ethylfluorene, 9- | 0.748 | 0.661 | Methylfluoranthene, 2- | 0.845 | ND | C4-BT | 0.668 | 0.852 | tPAH | 0.567 | 0.979 |
| Methylfluorene, 1- | 0.439 | 0.756 | Methylpyrene, 1- | 0.774 | 0.985 | C1-NAP | 0.002 | 0.016 |  |  |  |
| Propylfluorene, 9-n- | 0.962 | 0.913 | Dimethylpyrene, 4,5- | ND | ND | C2-NAP | 0.067 | 0.173 |  |  |  |
| Dibenzothiophene | 0.137 | 0.318 | Propylpyrene, 1-n- | ND | ND | C3-NAP | 0.369 | 0.646 |  |  |  |
