# Supplementary material for: Oil droplet fouling and differential toxicokinetics of polycyclic aromatic hydrocarbons in embryos of Atlantic haddock and cod
Source: PLoS One. 2017 Jul 5;12(7):e0180048. doi: 10.1371/journal.pone.0180048 (PMC5497984; doi:10.1371/journal.pone.0180048)
Supplement: S7 Table — Uptake of PAHs and alkylated PAHs in cod and haddock embryos. Given as pg/embryo with one SD (n = 3). LOQ = limit of quantification. NA = not analysed. (DOC) [file pone.0180048.s017.doc]

**Table S7. Body burden data.** Uptake of PAHs and alkylated PAHs in cod and haddock embryos. Given as pg/embryo with one SD (n=3). LOQ=limit of quantification. NA=not analysed.

| **Body burden**  **(pg/embryo)** | **Cod Ctr**  **Day 0** | **Cod Ctr**  **Day 3** | **Cod Ctr**  **Day 9** | **Cod Ctr**  **Day 11** | **Cod Ctr**  **Day 13** | **Haddock**  **Ctr Day 0** | **Haddock**  **Ctr Day 3** | **Haddock**  **Ctr Day 9** | **Haddock**  **Ctr Day 10** | **Haddock**  **Ctr Day 12** |
| --- | --- | --- | --- | --- | --- | --- | --- | --- | --- | --- |
| BIP | <LOQ | 1 ± 0.2 | 1.09 ± 0.03 | 0.2 ± 0.2 | 0.53 ± 0.06 | <LOQ | 0.8 ± 0.8 | 0.3 ± 0.1 | 0.06 ± 0.05 | <LOQ |
| BT | <LOQ | <LOQ | 0.01 ± 0.01 | <LOQ | <LOQ | <LOQ | <LOQ | 0.01 ± 0.01 | <LOQ | <LOQ |
| BT-2,5 | <LOQ | <LOQ | 0.04 ± 0.01 | <LOQ | <LOQ | <LOQ | <LOQ | 0.03 ± 0.01 | <LOQ | <LOQ |
| BT-2,5,7 | <LOQ | <LOQ | 0.06 ± 0.01 | <LOQ | <LOQ | <LOQ | <LOQ | 0.04 ± 0.01 | <LOQ | <LOQ |
| NAP | <LOQ | 0.1 ± 0.1 | 0.45 ± 0.04 | <LOQ | 0.05 ± 0.09 | <LOQ | 0.3 ± 0.5 | 0.2 ± 0.3 | 0.01 ± 0.02 | <LOQ |
| NAP-2 | <LOQ | 0.7 ± 0.04 | 1.6 ± 0.2 | 0.05 ± 0.06 | 0.4 ± 0.3 | 0.01 ± 0.02 | 1.4 ± 1.2 | 0.6 ± 0.4 | 0.1 ± 0.1 | <LOQ |
| NAP-1 | <LOQ | 0.4 ± 0.1 | 0.8 ± 0.4 | <LOQ | 0.1 ± 0.1 | <LOQ | 0.6 ± 0.5 | 0.1 ± 0.2 | <LOQ | <LOQ |
| NAP-2,6&2,7 | <LOQ | 1.26 ± 0.05 | 1.69 ± 0.09 | 0.4 ± 0.3 | 0.5 ± 0.04 | 0.07 ± 0.03 | 2 ± 1 | 0.6 ± 0.2 | 0.11 ± 0.09 | <LOQ |
| NAP-1,4 | <LOQ | 0.93 ± 0.06 | 1.54 ± 0.08 | 0.4 ± 0.2 | 0.47 ± 0.03 | 0.02 ± 0.03 | 1.2 ± 0.8 | 0.6 ± 0.2 | 0.08 ± 0.07 | <LOQ |
| NAP-1,3&2,3 | 0.04 ± 0.03 | 0.57 ± 0.02 | 1.2 ± 0.05 | 0.4 ± 0.2 | 0.4 ± 0.02 | 0.12 ± 0.02 | 0.7 ± 0.4 | 0.5 ± 0.2 | 0.13 ± 0.04 | 0.06 ± 0.02 |
| NAP-1,3,7 | <LOQ | 0.59 ± 0.09 | 0.31 ± 0.03 | 0.11 ± 0.03 | 0.08 ± 0.02 | 0.01 ± 0.02 | 0.7 ± 0.2 | 0.2 ± 0.2 | 0.07 ± 0.06 | 0.01 ± 0.02 |
| NAP-2,3,5 | 0.3 ± 0.1 | 2.01 ± 0.08 | 1.8 ± 0.2 | 0.9 ± 0.4 | 0.77 ± 0.04 | 0.14 ± 0.01 | 2.2 ± 0.6 | 1.5 ± 0.7 | 0.64 ± 0.12 | 0.14 ± 0.04 |
| NAP-1,2,3 | <LOQ | 0.04 ± 0.01 | 0.06 ± 0.02 | 0.01 ± 0.01 | 0.01 ± 0.01 | <LOQ | 0.04 ± 0.01 | 0.03 ± 0.04 | <LOQ | <LOQ |
| NAP-1,2,5,6 | <LOQ | 0.13 ± 0.03 | 0.06 ± 0.03 | 0.01 ± 0.01 | 0.03 ± 0.01 | <LOQ | 0.15 ± 0.03 | 0.07 ± 0.08 | <LOQ | <LOQ |
| NAP-1,4,6,7 | <LOQ | 0.15 ± 0.04 | 0.03 ± 0.02 | 0.01 ± 0.01 | 0.01 ± 0.01 | <LOQ | 0.12 ± 0.03 | 0.01 ± 0.02 | <LOQ | <LOQ |
| ACY | 0.05 ± 0.01 | <LOQ | 0.01 ± 0.01 | <LOQ | <LOQ | <LOQ | <LOQ | <LOQ | <LOQ | <LOQ |
| ACE | <LOQ | <LOQ | 0.03 ± 0.03 | <LOQ | 0.01 ± 0.02 | <LOQ | 0.04 ± 0.05 | <LOQ | <LOQ | <LOQ |
| DBF | <LOQ | 0.21 ± 0.09 | 0.19 ± 0.06 | <LOQ | 0.2 ± 0.2 | <LOQ | 0.1 ± 0.2 | <LOQ | <LOQ | <LOQ |
| FLU | <LOQ | 0.39 ± 0.02 | 0.5 ± 0.1 | <LOQ | 0.1 ± 0.1 | <LOQ | 0.3 ± 0.2 | 0.1 ± 0.1 | <LOQ | <LOQ |
| FLU-9et | <LOQ | <LOQ | 0.02 ± 0.02 | <LOQ | <LOQ | <LOQ | <LOQ | 0.01 ± 0.01 | <LOQ | <LOQ |
| FLU-1 | 0.01 ± 0 | 0.45 ± 0.05 | 0.21 ± 0.04 | 0.09 ± 0.04 | 0.08 ± 0.04 | 0.04 ± 0.01 | 0.27 ± 0.03 | 0.13 ± 0.07 | 0.04 ± 0.01 | 0.02 ± 0.01 |
| FLU-9pro | <LOQ | <LOQ | 0.01 ± 0.02 | <LOQ | <LOQ | <LOQ | <LOQ | <LOQ | <LOQ | <LOQ |
| DBT | <LOQ | 0.3 ± 0.1 | 0.2 ± 0.1 | <LOQ | 0.07 ± 0.08 | <LOQ | 0.07 ± 0.05 | 0.01 ± 0.01 | <LOQ | <LOQ |
| DBT-4 | <LOQ | 0.4 ± 0.2 | 0.09 ± 0.03 | 0.02 ± 0.02 | 0.01 ± 0.01 | <LOQ | 0.11 ± 0.02 | 0.08 ± 0.08 | <LOQ | 0.01 ± 0.02 |
| DBT-4et | 0.02 ± 0 | 0.19 ± 0.09 | 0.11 ± 0.05 | 0.02 ± 0.01 | 0.01 ± 0.01 | <LOQ | 0.04 ± 0.01 | 0.1 ± 0.2 | 0.01 ± 0.01 | 0.02 ± 0.02 |
| DBT-4pro | <LOQ | 0.16 ± 0.07 | 0.19 ± 0.08 | 0.01 ± 0.01 | <LOQ | <LOQ | 0.03 ± 0.02 | 0.3 ± 0.4 | <LOQ | 0.01 ± 0.01 |
| DBT-4but | 0.01 ± 0 | 0.2 ± 0.1 | 0.13 ± 0.03 | 0.02 ± 0.02 | 0.02 ± 0.01 | <LOQ | 0.02 ± 0.01 | 0.3 ± 0.4 | 0.01 ± 0.01 | 0.02 ± 0.02 |
| PHE | <LOQ | 0.9 ± 0.3 | 0.37 ± 0.09 | <LOQ | 0.4 ± 0.4 | <LOQ | 0.1 ± 0.2 | 0.03 ± 0.04 | <LOQ | 0.05 ± 0.08 |
| ANT | <LOQ | <LOQ | <LOQ | <LOQ | <LOQ | <LOQ | <LOQ | <LOQ | <LOQ | <LOQ |
| PHE-3 | <LOQ | 0.6 ± 0.4 | 0.2 ± 0.1 | 0.01 ± 0.02 | 0.03 ± 0.03 | <LOQ | 0.09 ± 0.02 | 0.2 ± 0.3 | <LOQ | 0.04 ± 0.04 |
| PHE-2 | <LOQ | 0.7 ± 0.5 | 0.2 ± 0.05 | 0 ± 0.01 | 0.03 ± 0.04 | <LOQ | 0.1 ± 0.05 | 0.2 ± 0.3 | <LOQ | 0.02 ± 0.02 |
| PHE-9 | 0.03 ± 0.03 | 1.0 ± 0.7 | 0.5 ± 0.3 | 0.08 ± 0.05 | 0.06 ± 0.03 | 0.03 ± 0.01 | 0.23 ± 0.04 | 0.5 ± 0.6 | 0.04 ± 0.02 | 0.09 ± 0.09 |
| PHE-1 | 0.01 ± 0.01 | 0.7 ± 0.5 | 0.4 ± 0.2 | 0.02 ± 0.02 | 0.03 ± 0.02 | <LOQ | 0.08 ± 0.01 | 0.3 ± 0.4 | <LOQ | 0.05 ± 0.05 |
| PHE-3,6 | 0.03 ± 0.01 | 0.4 ± 0.3 | 0.4 ± 0.1 | 0.05 ± 0.05 | 0.02 ± 0.01 | 0.04 ± 0.01 | 0.06 ± 0.02 | 0.6 ± 0.7 | 0.03 ± 0.01 | 0.05 ± 0.05 |
| PHE-1,7 | 0.08 ± 0.01 | 0.6 ± 0.3 | 0.7 ± 0.3 | 0.12 ± 0.07 | 0.09 ± 0.03 | 0.08 ± 0.01 | 0.02 ± 0.02 | 1 ± 1 | 0.08 ± 0.02 | 0.14 ± 0.07 |
| PHE-1,2 | <LOQ | 0.14 ± 0.07 | 0.11 ± 0.04 | <LOQ | <LOQ | <LOQ | <LOQ | 0.1 ± 0.2 | <LOQ | <LOQ |
| PHE-2,6,9 | 0.04 ± 0.01 | 0.6 ± 0.2 | 0.8 ± 0.2 | 0.08 ± 0.09 | 0.01 ± 0.01 | <LOQ | 0.02 ± 0.02 | 1 ± 2 | 0.01 ± 0.01 | 0.06 ± 0.07 |
| PHE-1,2,6 | <LOQ | <LOQ | 0.07 ± 0.06 | 0 ± 0.01 | 0 ± 0.01 | <LOQ | <LOQ | 0.2 ± 0.3 | <LOQ | 0 ± 0.01 |
| PHE-1,2,7 | <LOQ | <LOQ | 0.01 ± 0.02 | 0.02 ± 0.02 | <LOQ | <LOQ | <LOQ | 0.06 ± 0.09 | <LOQ | 0.02 ± 0.01 |
| PHE-1,2,6,9 | <LOQ | <LOQ | 0.03 ± 0.02 | <LOQ | <LOQ | <LOQ | <LOQ | 0.1 ± 0.1 | <LOQ | 0.01 ± 0.01 |
| FLA | 0.24 ± 0.03 | 0.5 ± 0.1 | 0.16 ± 0.04 | 0.01 ± 0.01 | 0.06 ± 0.04 | 0.53 ± 0.05 | 0.46 ± 0.05 | 0.2 ± 0.2 | 0.05 ± 0.02 | 0.02 ± 0.02 |
| PYR | 0.06 ± 0 | 0.4 ± 0.3 | 0.13 ± 0.04 | <LOQ | 0 ± 0.01 | 0.15 ± 0.01 | 0.11 ± 0.03 | 0.2 ± 0.2 | <LOQ | <LOQ |
| FLA-2 | 0.01 ± 0.02 | <LOQ | <LOQ | 0.01 ± 0.02 | <LOQ | 0.04 ± 0.01 | <LOQ | <LOQ | 0.01 ± 0.02 | 0.03 ± 0.01 |
| PYR-1 | 0.01 ± 0.01 | 0.2 ± 0.3 | 0.08 ± 0.02 | 0.01 ± 0.02 | 0.02 ± 0.01 | <LOQ | <LOQ | 0.1 ± 0.2 | 0.02 ± 0.01 | 0.01 ± 0.01 |
| PYR-4,5 | <LOQ | 0.14 ± 0.07 | <LOQ | <LOQ | <LOQ | <LOQ | <LOQ | <LOQ | <LOQ | <LOQ |
| PYR-1pro | <LOQ | <LOQ | <LOQ | <LOQ | <LOQ | <LOQ | <LOQ | <LOQ | <LOQ | <LOQ |
| PYR-1et | <LOQ | <LOQ | <LOQ | <LOQ | <LOQ | <LOQ | <LOQ | <LOQ | <LOQ | <LOQ |
| PYR-1but | <LOQ | 0.1 ± 0.1 | <LOQ | <LOQ | 0.01 ± 0.02 | <LOQ | <LOQ | <LOQ | <LOQ | <LOQ |
| BAA | <LOQ | <LOQ | 0.09 ± 0.01 | <LOQ | <LOQ | <LOQ | <LOQ | 0.07 0.05 | <LOQ | <LOQ |
| CHR | 0.03 ± 0.02 | 0.1 ± 0.1 | 0.29 ± 0.09 | 0.01 ± 0.02 | <LOQ | 0.11 ± 0.02 | 0.01 ± 0.01 | 0.5 ± 0.6 | 0.01 ± 0.01 | 0.01 ± 0.02 |
| CHR-1 | <LOQ | 0.1 ± 0.2 | 0.09 ± 0.02 | <LOQ | <LOQ | <LOQ | <LOQ | 0.1 ± 0.2 | <LOQ | <LOQ |
| CHR-6et | <LOQ | <LOQ | <LOQ | <LOQ | <LOQ | <LOQ | <LOQ | <LOQ | <LOQ | <LOQ |
| CHR-6pro | <LOQ | <LOQ | <LOQ | <LOQ | <LOQ | <LOQ | <LOQ | <LOQ | <LOQ | <LOQ |
| CHR-6but | <LOQ | <LOQ | <LOQ | <LOQ | <LOQ | <LOQ | <LOQ | <LOQ | <LOQ | <LOQ |
| BBF | <LOQ | 0.2 ± 0.3 | 0.11 ± 0.02 | <LOQ | <LOQ | <LOQ | <LOQ | 0.2 ± 0.3 | <LOQ | <LOQ |
| BKF | <LOQ | <LOQ | <LOQ | <LOQ | <LOQ | <LOQ | <LOQ | <LOQ | <LOQ | <LOQ |
| BEP | <LOQ | 0.4 ± 0.6 | 0.13 ± 0.03 | <LOQ | <LOQ | <LOQ | <LOQ | 0.3 ± 0.3 | <LOQ | <LOQ |
| BAP | <LOQ | 0.2 ± 0.3 | <LOQ | <LOQ | <LOQ | <LOQ | <LOQ | <LOQ | <LOQ | <LOQ |
| PER | <LOQ | <LOQ | <LOQ | <LOQ | <LOQ | <LOQ | <LOQ | <LOQ | <LOQ | <LOQ |
| IND | <LOQ | 0.1 ± 0.2 | <LOQ | <LOQ | <LOQ | <LOQ | <LOQ | <LOQ | <LOQ | <LOQ |
| DBA | <LOQ | <LOQ | <LOQ | <LOQ | <LOQ | <LOQ | <LOQ | <LOQ | <LOQ | <LOQ |
| BGP | <LOQ | 1 ± 2 | <LOQ | <LOQ | <LOQ | <LOQ | 0.06 ± 0.05 | 0.1 ± 0.1 | <LOQ | <LOQ |
| C1-BT | NA | 0 ± 0.01 | 0.01 ± 0.01 | NA | NA | NA | 0.02 ± 0.03 | 0.01 ± 0.01 | NA | NA |
| C2-BT | NA | 0.01 ± 0.02 | <LOQ | NA | NA | NA | 0.06 ± 0.05 | <LOQ | NA | NA |
| C3-BT | NA | 0.3 ± 0.3 | 1.1 ± 0.3 | NA | NA | NA | 0.2 ± 0.2 | 0.5 ± 0.6 | NA | NA |
| C4-BT | NA | 0.14 ± 0.03 | 0.03 ± 0.05 | NA | NA | NA | 0.14 ± 0.06 | 0.14 ± 0.07 | NA | NA |
| C1-NAP | NA | 1.1 ± 0.2 | 2.4 ± 0.5 | NA | NA | NA | 2 ± 2 | 0.7 ± 0.5 | NA | NA |
| C2-NAP | NA | 3.4 ± 0.3 | 5.4 ± 0.4 | NA | NA | NA | 4 ± 3 | 2.2 ± 0.7 | NA | NA |
| C3-NAP | NA | 5.68 ± 0.09 | 6.9 ± 0.2 | NA | NA | NA | 7 ± 2 | 5 ± 2 | NA | NA |
| C4-NAP | NA | 2.4 ± 0.3 | 2.4 ± 0.1 | NA | NA | NA | 3 ± 0.5 | 3 ± 1 | NA | NA |
| C1-FLU | NA | 0.7 ± 0.2 | 0.2 ± 0.4 | NA | NA | NA | 0.4 ± 0.3 | <LOQ | NA | NA |
| C2-FLU | NA | 0.7 ± 0.6 | 0.4 ± 0.3 | NA | NA | NA | 0.2 ± 0.2 | 0.6 ± 0.5 | NA | NA |
| C3-FLU | NA | 1.4 ± 0.8 | 1.1 ± 0.4 | NA | NA | NA | 0.23 ± 0.08 | 2 ± 2 | NA | NA |
| C1-DBT | NA | 0.8 ± 0.4 | 0.22 ± 0.09 | NA | NA | NA | 0.18 ± 0.02 | 0.2 ± 0.2 | NA | NA |
| C2-DBT | NA | 3 ± 2 | 2 ± 1 | NA | NA | NA | 0.4 ± 0.1 | 3 ± 4 | NA | NA |
| C3-DBT | NA | 2.6 ± 1 | 2.9 ± 0.9 | NA | NA | NA | 0.4 ± 0.1 | 5 ± 7 | NA | NA |
| C4-DBT | NA | 5 ± 5 | 4 ± 1 | NA | NA | NA | 0.3 ± 0.1 | 8 ± 11 | NA | NA |
| C1-PHE | NA | 3 ± 2 | 1.4 ± 0.6 | NA | NA | NA | 0.5 ± 0.06 | 1 ± 1 | NA | NA |
| C2-PHE | NA | 6 ± 3 | 7 ± 2 | NA | NA | NA | 0.5 ± 0.3 | 8 ± 10 | NA | NA |
| C3-PHE | NA | 5 ± 2 | 6 ± 2 | NA | NA | NA | 1 ± 0.2 | 10 ± 12 | NA | NA |
| C4-PHE | NA | 7 ± 4 | 7 ± 1 | NA | NA | NA | 0.05 ± 0.08 | 12 ± 15 | NA | NA |
| C1-PYR | NA | 3 ± 2 | 1.9 ± 0.5 | NA | NA | NA | 0.16 ± 0.04 | 3 ± 3 | NA | NA |
| C2-PYR | NA | 4 ± 4 | 3.3 ± 0.6 | NA | NA | NA | 0.16 ± 0.04 | 5 ± 6 | NA | NA |
| C3-PYR | NA | 9 ± 11 | 3.7 ± 0.6 | NA | NA | NA | 0.52 ± 0.07 | 6 ± 7 | NA | NA |
| C1-CHR | NA | 0.6 ± 0.5 | 0.99 ± 0.07 | NA | NA | NA | 0.17 ± 0.04 | 2 ± 3 | NA | NA |
| C2-CHR | NA | 0.6 ± 0.6 | 1 ± 0.7 | NA | NA | NA | 0.05 ± 0.04 | 2 ± 2 | NA | NA |
| C3-CHR | NA | 7 ± 10 | 2 ± 1 | NA | NA | NA | 0.5 ± 0.1 | 3 ± 3 | NA | NA |
| C4-CHR | NA | 21 ± 32 | 3 ± 1 | NA | NA | NA | 0.7 ± 0.2 | 5 ± 6 | NA | NA |
| ΣPAH | 1 ± 0 | 20 ± 6 | 18 ± 2 | 3 ± 2 | 4 ± 2 | 1 ± 0 | 12 ± 8 | 12 ± 10 | 1.3 ± 0.6 | 1 ± 1 |
| tPAH |  | 99 ± 74 | 69 ± 11 |  |  |  | 25 ± 10 | 90 ± 97 |  |  |

Table S7 continued.

| **Body burden**  **(pg/embryo)** | **Cod 0.15 µg/L**  **Day 3** | **Cod 0.15 µg/L**  **Day 9** | **Cod 0.29 µg/L**  **Day 1** | **Cod 0.29 µg/L**  **Day 2** | **Cod 0.29 µg/L**  **Day 3** | **Cod 0.29 µg/L**  **Day 5** | **Cod 0.29 µg/L**  **Day 7** | **Cod 0.29 µg/L**  **Day 9** |
| --- | --- | --- | --- | --- | --- | --- | --- | --- |
| BIP | 0.6 ± 0.4 | 1.1 ± 0.1 | 1.4 ± 0.5 | 2 ± 2 | 1.5 ± 0.2 | 1.9 ± 0.2 | 3.2 ± 0.1 | 2.9 ± 0.4 |
| BT | <LOQ | 0.003 ± 0.01 | <LOQ | <LOQ | <LOQ | <LOQ | <LOQ | 0.01 ± 0.01 |
| BT-2,5 | <LOQ | 0.03 ± 0.01 | <LOQ | <LOQ | <LOQ | <LOQ | <LOQ | 0.07 ± 0 |
| BT-2,5,7 | 0.03 ± 0.06 | 0.06 ± 0 | 0.08 ± 0.02 | 0.09 ± 0.09 | 0.13 ± 0.01 | 0.11 ± 0.01 | 0.13 ± 0.01 | 0.1 ± 0.01 |
| NAP | 0.2 ± 0.2 | <LOQ | 0.05 ± 0.09 | 0.1 ± 0.1 | 0.3 ± 0.3 | 0.5 ± 0.8 | 0.8 ± 0.2 | 0.4 ± 0.4 |
| NAP-2 | 1.2 ± 0.5 | 1.6 ± 0.2 | 3.1 ± 0.8 | 3 ± 3 | 3 ± 0.5 | 2.3 ± 0.2 | 5.56 ± 0.08 | 4.9 ± 0.5 |
| NAP-1 | 0.1 ± 0.2 | 0.4 ± 0.1 | 0.7 ± 0.3 | 1 ± 0.9 | 0.8 ± 0.3 | 0.72 ± 0.07 | 1.9 ± 0.2 | 1.7 ± 0.4 |
| NAP-2,6&2,7 | 4 ± 2 | 1.8 ± 0.1 | 5 ± 2 | 7 ± 7 | 6.7 ± 0.5 | 5.6 ± 0.5 | 7.9 ± 0.3 | 4.4 ± 0.2 |
| NAP-1,4 | 3 ± 1 | 1.7 ± 0.2 | 2.7 ± 1 | 5 ± 5 | 4.7 ± 0.4 | 4.4 ± 0.2 | 6.3 ± 0.3 | 4.5 ± 0.2 |
| NAP-1,3&2,3 | 1.9 ± 0.6 | 1.3 ± 0.1 | 2 ± 0.6 | 3 ± 3 | 3.1 ± 0.2 | 2.8 ± 0.1 | 4.3 ± 0.1 | 3.3 ± 0.2 |
| NAP-1,3,7 | 1.7 ± 0.7 | 0.16 ± 0.08 | 1.2 ± 0.6 | 3 ± 3 | 2.8 ± 0.3 | 1.88 ± 0.04 | 1.8 ± 0.2 | 0.5 ± 0.1 |
| NAP-2,3,5 | 7 ± 3 | 2.2 ± 0.1 | 4 ± 2 | 11 ± 12 | 10.3 ± 0.3 | 8.8 ± 0.3 | 10 ± 1 | 4.7 ± 0.3 |
| NAP-1,2,3 | 0.2 ± 0.1 | 0.09 ± 0.01 | 0.14 ± 0.07 | 0.5 ± 0.4 | 0.43 ± 0.03 | 0.42 ± 0.03 | 0.49 ± 0.06 | 0.25 ± 0.01 |
| NAP-1,2,5,6 | 0.6 ± 0.3 | 0.05 ± 0.03 | 0.2 ± 0.1 | 1 ± 0.8 | 0.69 ± 0.02 | 0.5 ± 0.1 | 0.49 ± 0.05 | 0.25 ± 0.05 |
| NAP-1,4,6,7 | 0.5 ± 0.4 | 0.003 ± 0.006 | 0.5 ± 0.3 | 1.7 ± 1 | 0.65 ± 0.06 | 0.28 ± 0.02 | 0.24 ± 0.03 | 0.14 ± 0.04 |
| ACY | <LOQ | <LOQ | <LOQ | <LOQ | <LOQ | <LOQ | <LOQ | <LOQ |
| ACE | <LOQ | <LOQ | <LOQ | 0.05 ± 0.09 | <LOQ | 0 ± 0.01 | 0.1 ± 0.1 | 0.12 ± 0.09 |
| DBF | <LOQ | 0.01 ± 0.01 | <LOQ | 0.4 ± 0.6 | <LOQ | 0.48 ± 0.05 | 0.9 ± 0.2 | 0.7 ± 0.3 |
| FLU | 0.3 ± 0.4 | 0.3 ± 0.2 | 0.9 ± 0.5 | 2 ± 2 | 0.8 ± 0.3 | 1.05 ± 0.05 | 1.9 ± 0.1 | 1.6 ± 0.3 |
| FLU-9et | 0.11 ± 0.08 | 0.02 ± 0.02 | <LOQ | 0.05 ± 0.08 | 0.09 ± 0.03 | 0.12 ± 0.09 | 0.17 ± 0.03 | 0.11 ± 0.01 |
| FLU-1 | 1.6 ± 0.8 | 0.33 ± 0.01 | 1.8 ± 0.7 | 5 ± 4 | 3.3 ± 0.2 | 1.7 ± 0.3 | 2 ± 0.4 | 0.88 ± 0.07 |
| FLU-9pro | <LOQ | <LOQ | <LOQ | <LOQ | <LOQ | <LOQ | 0.01 ± 0.02 | 0.02 ± 0.02 |
| DBT | 0.8 ± 0.4 | 0.25 ± 0.07 | 0.7 ± 0.4 | 2 ± 2 | 1.67 ± 0.09 | 1.3 ± 0.1 | 1.6 ± 0.1 | 1.2 ± 0.2 |
| DBT-4 | 1 ± 0.4 | 0.06 ± 0.03 | 0.8 ± 0.4 | 2 ± 2 | 1.8 ± 0.1 | 0.68 ± 0.03 | 0.52 ± 0.03 | 0.29 ± 0.05 |
| DBT-4et | 0.4 ± 0.2 | 0.02 ± 0.02 | 0.3 ± 0.1 | 0.8 ± 0.4 | 0.6 ± 0.1 | 0.14 ± 0.01 | 0.1 ± 0.01 | 0.09 ± 0.03 |
| DBT-4pro | 0.29 ± 0.08 | 0.05 ± 0.03 | 0.2 ± 0.1 | 0.6 ± 0.1 | 0.49 ± 0.08 | 0.15 ± 0.02 | 0.14 ± 0.02 | 0.14 ± 0.04 |
| DBT-4but | 0.14 ± 0.02 | 0.04 ± 0.02 | 0.13 ± 0.06 | 0.3 ± 0.03 | 0.21 ± 0.06 | 0.06 ± 0.03 | 0.08 ± 0.02 | 0.12 ± 0.03 |
| PHE | 2 ± 2 | 0.04 ± 0.08 | 3 ± 1 | 8 ± 4 | 4.5 ± 0.4 | 3.3 ± 0.2 | 3.16 ± 0.3 | 1.1 ± 0.3 |
| ANT | <LOQ | <LOQ | <LOQ | <LOQ | <LOQ | <LOQ | <LOQ | <LOQ |
| PHE-3 | 1.2 ± 0.6 | 0.04 ± 0.08 | 1.4 ± 0.7 | 4 ± 2 | 1.6 ± 0.3 | 0.34 ± 0.02 | 0.26 ± 0.04 | 0.2 ± 0.1 |
| PHE-2 | 1 ± 0.6 | 0.03 ± 0.06 | 1.4 ± 0.8 | 4 ± 2 | 1.3 ± 0.3 | 0.23 ± 0.02 | 0.17 ± 0.02 | 0.22 ± 0.09 |
| PHE-9 | 2.5 ± 0.8 | 0.2 ± 0.2 | 2 ± 1 | 6 ± 3 | 4.5 ± 0.6 | 1.6 ± 0.1 | 1.19 ± 0.08 | 0.8 ± 0.2 |
| PHE-1 | 1.2 ± 0.6 | 0.1 ± 0.1 | 1.6 ± 0.8 | 4 ± 2 | 1.5 ± 0.5 | 0.32 ± 0.03 | 0.26 ± 0.04 | 0.3 ± 0.1 |
| PHE-3,6 | 0.8 ± 0.3 | 0.12 ± 0.08 | 0.8 ± 0.4 | 2 ± 0.7 | 1.1 ± 0.3 | 0.31 ± 0.04 | 0.28 ± 0.04 | 0.3 ± 0.1 |
| PHE-1,7 | 0.7 ± 0.1 | 0.2 ± 0.1 | 1.1 ± 0.5 | 2.2 ± 0.8 | 0.6 ± 0.3 | 0.24 ± 0.06 | 0.24 ± 0.05 | 0.36 ± 0.09 |
| PHE-1,2 | 0.16 ± 0.05 | 0.04 ± 0.03 | 0.2 ± 0.09 | 0.5 ± 0.1 | 0.14 ± 0.07 | 0.05 ± 0.02 | 0.05 ± 0.01 | 0.05 ± 0.01 |
| PHE-2,6,9 | 0.7 ± 0.2 | 0.2 ± 0.1 | 1.1 ± 0.7 | 1.8 ± 0.7 | 0.8 ± 0.5 | 0.1 ± 0.06 | 0.2 ± 0.07 | 0.5 ± 0.2 |
| PHE-1,2,6 | 0.08 ± 0.03 | 0.02 ± 0.02 | 0.2 ± 0.1 | 0.3 ± 0.1 | 0.1 ± 0.1 | 0 ± 0.01 | 0.03 ± 0.02 | 0.06 ± 0.02 |
| PHE-1,2,7 | <LOQ | <LOQ | <LOQ | 0.03 ± 0.05 | <LOQ | <LOQ | <LOQ | 0 ± 0.01 |
| PHE-1,2,6,9 | <LOQ | <LOQ | 0.01 ± 0.01 | 0.05 ± 0.03 | 0.01 ± 0.02 | <LOQ | <LOQ | 0.01 ± 0.02 |
| FLA | 0.4 ± 0.1 | 0.02 ± 0.03 | 0.5 ± 0.3 | 1.2 ± 0.3 | 0.51 ± 0.02 | 0.1 ± 0.04 | 0.05 ± 0.01 | 0.06 ± 0.05 |
| PYR | 0.2 ± 0.1 | 0.01 ± 0.02 | 0.4 ± 0.2 | 1 ± 0.2 | 0.16 ± 0.05 | 0 ± 0.01 | 0.01 ± 0.02 | 0.06 ± 0.04 |
| FLA-2 | 0.02 ± 0.04 | <LOQ | 0.07 ± 0.06 | 0.2 ± 0.1 | <LOQ | <LOQ | <LOQ | <LOQ |
| PYR-1 | 0.06 ± 0.06 | 0.02 ± 0.02 | 0.2 ± 0.1 | 0.4 ± 0.2 | 0.03 ± 0.05 | <LOQ | 0.01 ± 0.02 | 0.06 ± 0.05 |
| PYR-4,5 | 0.1 ± 0.1 | <LOQ | 0.1 ± 0.2 | 0.3 ± 0.2 | 0.1 ± 0.1 | 0.01 ± 0.02 | <LOQ | <LOQ |
| PYR-1pro | <LOQ | <LOQ | <LOQ | <LOQ | <LOQ | <LOQ | <LOQ | <LOQ |
| PYR-1et | <LOQ | <LOQ | <LOQ | <LOQ | <LOQ | <LOQ | <LOQ | 0.01 ± 0 |
| PYR-1but | <LOQ | <LOQ | <LOQ | <LOQ | <LOQ | <LOQ | <LOQ | <LOQ |
| BAA | <LOQ | <LOQ | <LOQ | <LOQ | <LOQ | <LOQ | <LOQ | 0.02 ± 0.04 |
| CHR | 0.3 ± 0.2 | 0.07 ± 0.05 | 0.4 ± 0.3 | 0.9 ± 0.3 | 0.6 ± 0.2 | 0.01 ± 0.01 | 0.06 ± 0.04 | 0.2 ± 0.1 |
| CHR-1 | 0.06 ± 0 | <LOQ | 0.07 ± 0.03 | 0.15 ± 0.06 | 0.07 ± 0.02 | <LOQ | <LOQ | 0.03 ± 0.05 |
| CHR-6et | <LOQ | <LOQ | <LOQ | <LOQ | <LOQ | <LOQ | <LOQ | <LOQ |
| CHR-6pro | <LOQ | <LOQ | <LOQ | <LOQ | <LOQ | <LOQ | <LOQ | <LOQ |
| CHR-6but | <LOQ | <LOQ | <LOQ | <LOQ | <LOQ | <LOQ | <LOQ | <LOQ |
| BBF | 0.06 ± 0.03 | 0.07 ± 0.01 | 0.11 ± 0.07 | 0.3 ± 0.2 | <LOQ | <LOQ | <LOQ | 0.13 ± 0.03 |
| BKF | <LOQ | <LOQ | 0.02 ± 0.04 | 0.11 ± 0.06 | <LOQ | <LOQ | <LOQ | <LOQ |
| BEP | 0.15 ± 0.02 | 0.06 ± 0.02 | 0.2 ± 0.1 | 0.4 ± 0.3 | 0.22 ± 0.06 | <LOQ | <LOQ | 0.14 ± 0.03 |
| BAP | <LOQ | <LOQ | <LOQ | <LOQ | <LOQ | <LOQ | <LOQ | <LOQ |
| PER | <LOQ | <LOQ | <LOQ | <LOQ | <LOQ | <LOQ | <LOQ | <LOQ |
| IND | <LOQ | <LOQ | <LOQ | 0.02 ± 0.04 | <LOQ | <LOQ | <LOQ | <LOQ |
| DBA | <LOQ | <LOQ | <LOQ | <LOQ | <LOQ | <LOQ | <LOQ | <LOQ |
| BGP | <LOQ | <LOQ | <LOQ | 0.4 ± 0.7 | 0.1 ± 0.1 | <LOQ | <LOQ | 0.01 ± 0.02 |
| C1-BT | <LOQ | 0.01 ± 0.01 | NA | NA | <LOQ | NA | NA | 0.01 ± 0.01 |
| C2-BT | <LOQ | <LOQ | NA | NA | <LOQ | NA | NA | <LOQ |
| C3-BT | 1 ± 0.2 | 0.3 ± 0.1 | NA | NA | 1.1 ± 0.07 | NA | NA | 0.94 ± 0.08 |
| C4-BT | 0.2 ± 0.1 | 0.09 ± 0.02 | NA | NA | 0.32 ± 0.02 | NA | NA | 0.26 ± 0.06 |
| C1-NAP | 1.4 ± 0.7 | 2 ± 0.4 | NA | NA | 3.8 ± 0.8 | NA | NA | 6.6 ± 0.9 |
| C2-NAP | 10 ± 3 | 6.1 ± 0.7 | NA | NA | 17 ± 1 | NA | NA | 15.9 ± 0.4 |
| C3-NAP | 20 ± 7 | 6.9 ± 0.5 | NA | NA | 29 ± 1 | NA | NA | 14.9 ± 0.8 |
| C4-NAP | 10 ± 4 | 2.5 ± 0.3 | NA | NA | 13 ± 0.5 | NA | NA | 6 ± 0.5 |
| C1-FLU | 4 ± 2 | <LOQ | NA | NA | 5.5 ± 0.5 | NA | NA | 0.7 ± 0.4 |
| C2-FLU | 2 ± 1 | 0.1 ± 0.2 | NA | NA | 3.4 ± 0.7 | NA | NA | 0.8 ± 0.2 |
| C3-FLU | 1.8 ± 0.8 | 0.2 ± 0.2 | NA | NA | 2.4 ± 0.8 | NA | NA | 1 ± 0.2 |
| C1-DBT | 2 ± 0.8 | 0.14 ± 0.07 | NA | NA | 3.3 ± 0.3 | NA | NA | 0.6 ± 0.1 |
| C2-DBT | 5 ± 2 | 0.4 ± 0.4 | NA | NA | 6 ± 2 | NA | NA | 1.4 ± 0.6 |
| C3-DBT | 3 ± 1 | 0.6 ± 0.4 | NA | NA | 4 ± 1 | NA | NA | 1.9 ± 0.7 |
| C4-DBT | 2.9 ± 0.5 | 1.2 ± 0.5 | NA | NA | 5 ± 1 | NA | NA | 3.8 ± 0.9 |
| C1-PHE | 6 ± 3 | 0.4 ± 0.4 | NA | NA | 9 ± 2 | NA | NA | 1.6 ± 0.5 |
| C2-PHE | 8 ± 3 | 1 ± 1 | NA | NA | 8 ± 3 | NA | NA | 3 ± 1 |
| C3-PHE | 5 ± 1 | 1.5 ± 0.8 | NA | NA | 6 ± 2 | NA | NA | 4 ± 1 |
| C4-PHE | 4.6 ± 0.8 | 1.3 ± 0.7 | NA | NA | 6 ± 2 | NA | NA | 5 ± 1 |
| C1-PYR | 1.9 ± 0.5 | 0.7 ± 0.2 | NA | NA | 2.1 ± 0.9 | NA | NA | 1.8 ± 0.5 |
| C2-PYR | 2.1 ± 0.6 | 0.7 ± 0.4 | NA | NA | 3 ± 1 | NA | NA | 2.1 ± 0.5 |
| C3-PYR | 2 ± 0.3 | 0.9 ± 0.4 | NA | NA | 4 ± 1 | NA | NA | 2.9 ± 0.5 |
| C1-CHR | 0.6 ± 0.1 | 0.3 ± 0.1 | NA | NA | 0.8 ± 0.4 | NA | NA | 0.8 ± 0.1 |
| C2-CHR | 0.4 ± 0.1 | 0.5 ± 0.2 | NA | NA | 0.8 ± 0.3 | NA | NA | 1 ± 1 |
| C3-CHR | 0.9 ± 0.3 | 0.6 ± 0.2 | NA | NA | 1.4 ± 0.4 | NA | NA | 2 ± 0.3 |
| C4-CHR | 0.8 ± 0.3 | 1.7 ± 0.5 | NA | NA | 3 ± 1 | NA | NA | 4 ± 0.6 |
| ΣPAH | 37 ± 16 | 13 ± 2 | 41 ± 17 | 88 ± 66 | 62 ± 2 | 42 ± 3 | 56 ± 2 | 38 ± 4 |
| tPAH | 101 ± 33 | 32 ± 8 |  |  | 147 ± 15 |  |  | 91 ± 11 |

Table S7 continued.

| **Body burden (pg/embryo)** | **Cod 2.8 µg/L Day 1** | **Cod 2.8 µg/L Day 2** | **Cod 2.8 µg/L Day 3** | **Cod 2.8 µg/L Day 5** | **Cod 2.8 µg/L Day 7** | **Cod 2.8 µg/L Day 9** | **Cod 2.8 µg/L Day 11** | **Cod 2.8 µg/L Day 12** | **Cod 2.8 µg/L Day 13** |
| --- | --- | --- | --- | --- | --- | --- | --- | --- | --- |
| BIP | 15.8 ± 0.6 | 25 ± 2 | 19.2 ± 0.5 | 16.9 ± 0.5 | 18.2 ± 0.5 | 15.4 ± 0.3 | 9.9 ± 0.8 | 0.66 ± 0.08 | 0.5 ± 0.3 |
| BT | 0.03 ± 0.04 | 0.01 ± 0.01 | <LOQ | 0.04 ± 0.02 | 0.03 ± 0.01 | 0.01 ± 0 | 0.02 ± 0.01 | 0.01 ± 0 | 0.01 ± 0.01 |
| BT-2,5 | 0.39 ± 0.03 | 0.73 ± 0.03 | 0.61 ± 0.05 | 0.47 ± 0.02 | 0.49 ± 0.02 | 0.38 ± 0.01 | 0.26 ± 0.01 | 0.02 ± 0.01 | 0.03 ± 0.01 |
| BT-2,5,7 | 0.4 ± 0.03 | 1.1 ± 0.02 | 1.1 ± 0.2 | 1.18 ± 0.08 | 0.92 ± 0.02 | 0.64 ± 0.04 | 0.41 ± 0.04 | <LOQ | <LOQ |
| NAP | 5 ± 2 | 6 ± 1 | 4.9 ± 0.3 | 4.8 ± 0.2 | 6 ± 2 | 3.6 ± 0.3 | 1.8 ± 0.5 | 0.2 ± 0.2 | 0.2 ± 0.4 |
| NAP-2 | 39 ± 1 | 54 ± 7 | 37 ± 1 | 32 ± 1 | 42 ± 1 | 32.3 ± 0.2 | 22 ± 1 | 1 ± 0.2 | 0.8 ± 0.5 |
| NAP-1 | 15 ± 2 | 22 ± 3 | 14.9 ± 0.7 | 13.1 ± 0.5 | 16.9 ± 0.4 | 13.3 ± 0.2 | 9.9 ± 0.9 | 0.8 ± 0.2 | 1 ± 0.6 |
| NAP-2,6&2,7 | 38 ± 5 | 83 ± 3 | 76 ± 5 | 53 ± 2 | 50 ± 1 | 35 ± 0.5 | 22.7 ± 0.9 | 0.7 ± 0.2 | 0.6 ± 0.3 |
| NAP-1,4 | 45 ± 8 | 121 ± 8 | 56 ± 4 | 85 ± 4 | 82 ± 3 | 32.9 ± 0.3 | 42 ± 2 | 1.3 ± 0.2 | 1.4 ± 0.4 |
| NAP-1,3&2,3 | 8.2 ± 0.9 | 19 ± 1 | 35 ± 2 | 12.8 ± 0.8 | 12.5 ± 0.5 | 22.1 ± 0.3 | 6.5 ± 0.4 | 0.21 ± 0.01 | 0.22 ± 0.08 |
| NAP-1,3,7 | 6 ± 1 | 23 ± 2 | 41 ± 4 | 10.5 ± 0.8 | 6.4 ± 0.5 | 5 ± 0.5 | 5.5 ± 0.7 | 0.13 ± 0.02 | 0.05 ± 0.04 |
| NAP-2,3,5 | 14 ± 2 | 59 ± 5 | 140 ± 10 | 69 ± 4 | 50 ± 3 | 32.8 ± 0.7 | 22 ± 1 | 1.2 ± 0.1 | 0.6 ± 0.1 |
| NAP-1,2,3 | 0.9 ± 0.1 | 3.5 ± 0.2 | 6.7 ± 0.7 | 3.2 ± 0.1 | 2.3 ± 0.1 | 1.9 ± 0.2 | 1.12 ± 0.04 | 0.05 ± 0.01 | 0 ± 0.01 |
| NAP-1,2,5,6 | 1.3 ± 0.2 | 6.5 ± 0.4 | 12.6 ± 0.9 | 5.2 ± 0.3 | 3.2 ± 0.3 | 2.4 ± 0.3 | 1.6 ± 0.1 | 0.05 ± 0.01 | <LOQ |
| NAP-1,4,6,7 | 2.9 ± 0.5 | 13.6 ± 0.8 | 15.7 ± 0.9 | 3.9 ± 0.3 | 2.3 ± 0.1 | 1.9 ± 0.4 | 1.8 ± 0.2 | 0.05 ± 0.01 | <LOQ |
| ACY | 0.07 ± 0.05 | 0.04 ± 0.06 | <LOQ | 0.02 ± 0.03 | 0.03 ± 0.03 | 0 ± 0.01 | 0.02 ± 0.01 | 0.01 ± 0.01 | 0.03 ± 0.02 |
| ACE | 0.6 ± 0.3 | 1.3 ± 0.2 | 2.2 ± 0.4 | 1.16 ± 0.09 | 1.2 ± 0.1 | 1.08 ± 0.02 | 0.88 ± 0.08 | 0.04 ± 0.06 | 0.04 ± 0.06 |
| DBF | 7.6 ± 1 | 13 ± 0.8 | 9.5 ± 0.4 | 6.8 ± 0.5 | 7 ± 0.3 | 5.69 ± 0.07 | 4 ± 0.3 | 0.3 ± 0.1 | 0.1 ± 0.2 |
| FLU | 18 ± 2 | 32 ± 2 | 20.4 ± 0.8 | 13.7 ± 0.8 | 12.5 ± 0.5 | 9 ± 1 | 7.9 ± 0.3 | 0.27 ± 0.1 | 0.1 ± 0.2 |
| FLU-9et | 0.14 ± 0.02 | 0.59 ± 0.04 | 1.44 ± 0.09 | 1.16 ± 0.03 | 1.02 ± 0.05 | 0.96 ± 0.06 | 0.5 ± 0.01 | 0.1 ± 0.01 | 0.01 ± 0.01 |
| FLU-1 | 11 ± 1 | 45 ± 2 | 56 ± 3 | 16 ± 2 | 10 ± 0.6 | 6.8 ± 0.3 | 6.5 ± 0.4 | 0.5 ± 0.4 | 0.5 ± 0.1 |
| FLU-9pro | <LOQ | <LOQ | 0.19 ± 0.03 | <LOQ | <LOQ | 0.35 ± 0.05 | 0.19 ± 0.09 | 0.05 ± 0.03 | 0 ± 0.01 |
| DBT | 13 ± 2 | 36 ± 2 | 27.3 ± 0.3 | 17 ± 1 | 12.3 ± 0.5 | 7.7 ± 0.2 | 6.5 ± 0.5 | 0.13 ± 0.04 | 0.1 ± 0.1 |
| DBT-4 | 6 ± 1 | 29 ± 1 | 30 ± 2 | 11.2 ± 0.6 | 4.9 ± 0.3 | 2.8 ± 0.4 | 3.7 ± 0.2 | 0.08 ± 0 | 0 ± 0.01 |
| DBT-4et | 1.3 ± 0.3 | 7.1 ± 0.3 | 8 ± 0.5 | 2.4 ± 0.2 | 1.27 ± 0.03 | 0.9 ± 0.3 | 1.2 ± 0.1 | 0.04 ± 0.01 | <LOQ |
| DBT-4pro | 0.7 ± 0.2 | 3.3 ± 0.1 | 3.6 ± 0.3 | 1.8 ± 0.2 | 1.17 ± 0.06 | 0.9 ± 0.3 | 0.9 ± 0.1 | 0.08 ± 0.02 | 0 ± 0.01 |
| DBT-4but | 0.3 ± 0.1 | 0.95 ± 0.05 | 1.1 ± 0.1 | 0.44 ± 0.05 | 0.55 ± 0.05 | 0.6 ± 0.3 | 0.4 ± 0.1 | 0.06 ± 0.03 | 0.03 ± 0.01 |
| PHE | 19 ± 2 | 56 ± 2 | 78 ± 5 | 25 ± 1 | 14.5 ± 0.7 | 10.5 ± 0.6 | 7.7 ± 0.4 | 0.19 ± 0.14 | <LOQ |
| ANT | <LOQ | <LOQ | <LOQ | <LOQ | <LOQ | <LOQ | <LOQ | <LOQ | <LOQ |
| PHE-3 | 12 ± 2 | 50 ± 3 | 37 ± 3 | 8.3 ± 0.9 | 4.5 ± 0.2 | 3 ± 0.8 | 4.4 ± 0.3 | 0.08 ± 0.03 | <LOQ |
| PHE-2 | 12 ± 2 | 47 ± 3 | 34 ± 3 | 5.4 ± 0.6 | 3.4 ± 0.2 | 2.6 ± 0.7 | 3.5 ± 0.2 | 0.12 ± 0.01 | <LOQ |
| PHE-9 | 16 ± 3 | 66 ± 3 | 61 ± 4 | 28 ± 2 | 13 ± 0.7 | 7.2 ± 0.9 | 10.7 ± 0.2 | 0.28 ± 0.03 | 0.08 ± 0.03 |
| PHE-1 | 13 ± 2 | 50 ± 3 | 32 ± 2 | 6.7 ± 0.8 | 4.1 ± 0.2 | 2.8 ± 0.7 | 3.8 ± 0.6 | 0.05 ± 0 | <LOQ |
| PHE-3,6 | 4 ± 0.8 | 20 ± 1 | 13 ± 1 | 2 ± 2 | 3.3 ± 0.1 | 2.3 ± 0.7 | 2.9 ± 0.2 | 0.19 ± 0.02 | 0.07 ± 0.01 |
| PHE-1,7 | 4.8 ± 0.8 | 18 ± 1 | 10 ± 1 | 1.9 ± 0.4 | 2.5 ± 0.3 | 3 ± 1 | 1.7 ± 0.4 | 0.09 ± 0.03 | 0.05 ± 0.02 |
| PHE-1,2 | 1 ± 0.1 | 4 ± 0.2 | 1.52 ± 0.07 | 0.34 ± 0.07 | 0.31 ± 0.04 | 0.4 ± 0.2 | 0.29 ± 0.06 | <LOQ | <LOQ |
| PHE-2,6,9 | 2.6 ± 0.7 | 9 ± 1 | 7 ± 1 | 1.7 ± 0.1 | 2.7 ± 0.2 | 3 ± 1 | 1.9 ± 0.6 | 0.09 ± 0.08 | <LOQ |
| PHE-1,2,6 | 0.6 ± 0.1 | 1.7 ± 0.2 | 0.9 ± 0.2 | 0.33 ± 0.04 | 0.53 ± 0.02 | 0.4 ± 0.1 | 0.5 ± 0.2 | <LOQ | <LOQ |
| PHE-1,2,7 | 0.3 ± 0.1 | 0.69 ± 0.07 | 0.27 ± 0.04 | 0.13 ± 0.01 | 0.21 ± 0.02 | 0.13 ± 0.06 | 0.2 ± 0.1 | <LOQ | <LOQ |
| PHE-1,2,6,9 | 0.1 ± 0.1 | 0.31 ± 0.06 | 0.32 ± 0.08 | 0.01 ± 0.01 | 0.14 ± 0.03 | 0.2 ± 0.1 | 0.1 ± 0.05 | <LOQ | <LOQ |
| FLA | 1.7 ± 0.5 | 7.6 ± 0.7 | 6.9 ± 0.5 | 1.54 ± 0.16 | 0.73 ± 0.06 | 0.5 ± 0.2 | 0.5 ± 0.01 | <LOQ | <LOQ |
| PYR | 1.8 ± 0.3 | 8.4 ± 0.4 | 4.5 ± 0.3 | 0.69 ± 0.08 | 0.68 ± 0.06 | 0.8 ± 0.3 | 0.68 ± 0.05 | 0.03 ± 0.01 | <LOQ |
| FLA-2 | 0.4 ± 0.1 | 1.7 ± 0.2 | 1 ± 0.1 | 0.2 ± 0.01 | 0.34 ± 0.01 | <LOQ | 0.36 ± 0.02 | <LOQ | <LOQ |
| PYR-1 | 0.6 ± 0.1 | 2.1 ± 0.2 | 1.1 ± 0.2 | 0.26 ± 0.07 | 0.4 ± 0.03 | 0.4 ± 0.1 | 0.38 ± 0.05 | 0.07 ± 0.05 | 0.03 ± 0.01 |
| PYR-4,5 | <LOQ | <LOQ | 1.5 ± 0.2 | <LOQ | <LOQ | <LOQ | <LOQ | <LOQ | <LOQ |
| PYR-1pro | <LOQ | <LOQ | <LOQ | <LOQ | <LOQ | <LOQ | <LOQ | <LOQ | <LOQ |
| PYR-1et | 0 ± 0.01 | 0.07 ± 0.03 | <LOQ | <LOQ | 0.04 ± 0.03 | 0.05 ± 0.03 | 0.07 ± 0.02 | <LOQ | <LOQ |
| PYR-1but | 0.01 ± 0.01 | 0.03 ± 0.01 | <LOQ | 0.01 ± 0.01 | 0.03 ± 0.01 | <LOQ | 0.1 ± 0.1 | <LOQ | <LOQ |
| BAA | 0.35 ± 0.03 | 0.84 ± 0.01 | 0.09 ± 0.08 | 0.31 ± 0.06 | 0.27 ± 0.01 | 0.17 ± 0.06 | <LOQ | 0.05 ± 0.09 | <LOQ |
| CHR | 1 ± 0.3 | 4.2 ± 0.3 | 4.7 ± 0.5 | 1.15 ± 0.11 | 1.27 ± 0.13 | 1.2 ± 0.4 | 1 ± 0.2 | 0.06 ± 0.02 | <LOQ |
| CHR-1 | 0.12 ± 0.03 | 0.33 ± 0.02 | 0.32 ± 0.02 | 0.13 ± 0.02 | 0.18 ± 0.03 | 0.2 ± 0.1 | 0.09 ± 0.03 | <LOQ | <LOQ |
| CHR-6et | <LOQ | <LOQ | <LOQ | <LOQ | <LOQ | <LOQ | <LOQ | <LOQ | <LOQ |
| CHR-6pro | <LOQ | <LOQ | <LOQ | <LOQ | <LOQ | <LOQ | <LOQ | <LOQ | <LOQ |
| CHR-6but | <LOQ | <LOQ | <LOQ | <LOQ | <LOQ | <LOQ | <LOQ | <LOQ | <LOQ |
| BBF | 0.21 ± 0.03 | 0.43 ± 0.04 | <LOQ | 0.19 ± 0.02 | 0.29 ± 0.02 | 0.3 ± 0.2 | 0.23 ± 0.06 | 0.07 ± 0.01 | 0.07 ± 0 |
| BKF | 0.12 ± 0.03 | 0.22 ± 0.02 | 0.18 ± 0.04 | 0.11 ± 0.01 | 0.12 ± 0.02 | 0.17 ± 0.07 | <LOQ | <LOQ | <LOQ |
| BEP | 0.16 ± 0.06 | 0.46 ± 0.07 | 1 ± 0.1 | 0.15 ± 0.01 | 0.26 ± 0.04 | 0.4 ± 0.2 | 0.2 ± 0.06 | 0.05 ± 0.01 | <LOQ |
| BAP | <LOQ | 0.01 ± 0.02 | 0.3 ± 0.2 | <LOQ | <LOQ | <LOQ | <LOQ | <LOQ | <LOQ |
| PER | 0.08 ± 0.02 | 0.15 ± 0.02 | <LOQ | 0.1 ± 0.02 | 0.11 ± 0.03 | <LOQ | <LOQ | <LOQ | <LOQ |
| IND | <LOQ | <LOQ | <LOQ | <LOQ | 0.01 ± 0.01 | <LOQ | <LOQ | <LOQ | <LOQ |
| DBA | 0.36 ± 0.05 | 0.4 ± 0.08 | 0.07 ± 0.01 | 0.48 ± 0.09 | 0.35 ± 0.03 | <LOQ | <LOQ | <LOQ | <LOQ |
| BGP | <LOQ | 0.02 ± 0.04 | 0.12 ± 0.05 | <LOQ | 0.03 ± 0.05 | 0.11 ± 0.1 | 0.02 ± 0.02 | <LOQ | <LOQ |
| C1-BT | NA | NA | 0.11 ± 0.02 | NA | NA | 0.17 ± 0.01 | 0.3 ± 0.07 | NA | NA |
| C2-BT | NA | NA | 1.3 ± 0.1 | NA | NA | 1.46 ± 0.08 | 0.7 ± 0.2 | NA | NA |
| C3-BT | NA | NA | 9.9 ± 0.4 | NA | NA | 4.1 ± 0.2 | 4.6 ± 0.7 | NA | NA |
| C4-BT | NA | NA | 6.7 ± 0.1 | NA | NA | 2.5 ± 0.6 | 3 ± 0.3 | NA | NA |
| C1-NAP | NA | NA | 52 ± 2 | NA | NA | 45.6 ± 0.1 | 32 ± 2 | NA | NA |
| C2-NAP | NA | NA | 212 ± 11 | NA | NA | 128 ± 4 | 109 ± 3 | NA | NA |
| C3-NAP | NA | NA | 388 ± 25 | NA | NA | 121 ± 5 | 114 ± 20 | NA | NA |
| C4-NAP | NA | NA | 189 ± 9 | NA | NA | 59 ± 4 | 45 ± 11 | NA | NA |
| C1-FLU | NA | NA | 92 ± 5 | NA | NA | 11 ± 2 | 13 ± 2 | NA | NA |
| C2-FLU | NA | NA | 68 ± 4 | NA | NA | 13 ± 2 | 11 ± 1 | NA | NA |
| C3-FLU | NA | NA | 32 ± 2 | NA | NA | 11 ± 2 | 8.6 ± 0.7 | NA | NA |
| C1-DBT | NA | NA | 58 ± 4 | NA | NA | 6 ± 1 | 7.2 ± 0.4 | NA | NA |
| C2-DBT | NA | NA | 93 ± 5 | NA | NA | 14 ± 5 | 17 ± 1 | NA | NA |
| C3-DBT | NA | NA | 35 ± 2 | NA | NA | 12 ± 6 | 10 ± 1 | NA | NA |
| C4-DBT | NA | NA | 26 ± 4 | NA | NA | 16 ± 8 | 11 ± 2 | NA | NA |
| C1-PHE | NA | NA | 163 ± 11 | NA | NA | 16 ± 3 | 23 ± 1 | NA | NA |
| C2-PHE | NA | NA | 125 ± 10 | NA | NA | 25 ± 10 | 24 ± 3 | NA | NA |
| C3-PHE | NA | NA | 50 ± 5 | NA | NA | 21 ± 9 | 15 ± 3 | NA | NA |
| C4-PHE | NA | NA | 39 ± 6 | NA | NA | 24 ± 11 | 18 ± 2 | NA | NA |
| C1-PYR | NA | NA | 22 ± 2 | NA | NA | 8 ± 3 | 7.2 ± 0.2 | NA | NA |
| C2-PYR | NA | NA | 18 ± 3 | NA | NA | 10 ± 5 | 6.6 ± 0.7 | NA | NA |
| C3-PYR | NA | NA | 17 ± 4 | NA | NA | 12 ± 6 | 7 ± 1 | NA | NA |
| C1-CHR | NA | NA | 6 ± 1 | NA | NA | 4 ± 2 | 3.3 ± 0.8 | NA | NA |
| C2-CHR | NA | NA | 4 ± 1 | NA | NA | 4 ± 1 | 4 ± 1 | NA | NA |
| C3-CHR | NA | NA | 5 ± 1 | NA | NA | 5 ± 3 | 0.02 ± 0.03 | NA | NA |
| C4-CHR | NA | NA | 9 ± 3 | NA | NA | 8 ± 3 | 3 ± 1 | NA | NA |
| ΣPAH | 344 ± 36 | 954 ± 39 | 917 ± 46 | 469 ± 14 | 399 ± 9 | 276 ± 15 | 222 ± 8 | 10 ± 1 | 7 ± 3 |
| tPAH |  |  | 1902 ± 88 |  |  | 639 ± 98 | 538 ± 43 |  |  |

Table S7 continued.

| **Body burden (pg/embryo)** | **Cod 3.6 µg/L Day 1** | **Cod 3.6 µg/L Day 2** | **Cod 3.6 µg/L Day 3** | **Cod 3.6 µg/L Day 5** | **Cod 3.6 µg/L Day 7** | **Cod 3.6 µg/L Day 9** | **Cod 3.6 µg/L Day 11** | **Cod 3.6 µg/L Day 12** | **Cod 3.6 µg/L Day 13** |
| --- | --- | --- | --- | --- | --- | --- | --- | --- | --- |
| BIP | 16 ± 3 | 21.14 ± 0.07 | 24 ± 1 | 22.2 ± 0.4 | 24.6 ± 0.7 | 22 ± 1 | 9 ± 1 | 0.58 ± 0.04 | 0.2 ± 0.1 |
| BT | <LOQ | <LOQ | <LOQ | <LOQ | <LOQ | 0.01 ± 0.01 | <LOQ | <LOQ | <LOQ |
| BT-2,5 | 0.39 ± 0.08 | 0.61 ± 0.02 | 0.62 ± 0.04 | 0.61 ± 0.06 | 0.66 ± 0.03 | 0.57 ± 0.02 | 0.27 ± 0.03 | <LOQ | <LOQ |
| BT-2,5,7 | 0.5 ± 0.2 | 0.9 ± 0.1 | 1.2 ± 0.1 | 1.4 ± 0.1 | 1.3 ± 0.1 | 1 ± 0.03 | 0.5 ± 0.02 | <LOQ | <LOQ |
| NAP | 4.9 ± 0.4 | 5.7 ± 0.2 | 6.9 ± 0.7 | 4.7 ± 0.5 | 5.8 ± 0.8 | 4 ± 1 | 1.2 ± 0.3 | 0.1 ± 0.2 | 0.2 ± 0.3 |
| NAP-2 | 36 ± 5 | 44.4 ± 0.5 | 51 ± 3 | 38 ± 1 | 49 ± 3 | 46 ± 3 | 20 ± 2 | 0.5 ± 0.2 | 0.2 ± 0.3 |
| NAP-1 | 14 ± 2 | 18.84 ± 0.09 | 21 ± 1 | 16.7 ± 0.6 | 21 ± 1 | 19 ± 2 | 7.8 ± 0.9 | 0.2 ± 0.1 | 0.1 ± 0.2 |
| NAP-2,6&2,7 | 44 ± 10 | 70 ± 2 | 75 ± 3 | 68 ± 3 | 68.8 ± 0.6 | 53 ± 1 | 28 ± 2 | 1 ± 0.1 | 0.4 ± 0.2 |
| NAP-1,4 | 31 ± 7 | 53 ± 1 | 58 ± 2 | 59 ± 3 | 60.8 ± 0.7 | 50 ± 2 | 27 ± 2 | 0.93 ± 0.09 | 0.4 ± 0.2 |
| NAP-1,3&2,3 | 23 ± 5 | 37.4 ± 0.4 | 40 ± 2 | 42 ± 2 | 42.7 ± 0.9 | 33 ± 1 | 18 ± 2 | 1.1 ± 0.3 | 0.4 ± 0.09 |
| NAP-1,3,7 | 13 ± 5 | 31 ± 2 | 33 ± 3 | 26 ± 2 | 19 ± 2 | 9 ± 0.6 | 9 ± 0.7 | 0.3 ± 0.1 | 0.05 ± 0.04 |
| NAP-2,3,5 | 37 ± 13 | 84.3 ± 0.6 | 93 ± 3 | 95 ± 9 | 85 ± 1 | 51 ± 3 | 32 ± 1 | 2.6 ± 0.7 | 0.6 ± 0.2 |
| NAP-1,2,3 | 1.5 ± 0.6 | 3.7 ± 0.2 | 3.87 ± 0.03 | 4.5 ± 0.7 | 3.91 ± 0.09 | 3 ± 0.3 | 1.56 ± 0.07 | 0.09 ± 0.05 | 0.01 ± 0.01 |
| NAP-1,2,5,6 | 2 ± 1 | 6.7 ± 0.4 | 7.6 ± 0.2 | 6.8 ± 0.8 | 5.69 ± 0.09 | 4 ± 0.6 | 3.1 ± 0.3 | 0.18 ± 0.08 | <LOQ |
| NAP-1,4,6,7 | 5 ± 2 | 12.5 ± 0.5 | 10.3 ± 0.4 | 5.6 ± 0.8 | 4.5 ± 0.1 | 3.4 ± 0.7 | 3.6 ± 0.5 | 0.11 ± 0.04 | <LOQ |
| ACY | 0.04 ± 0.01 | <LOQ | <LOQ | 0.01 ± 0.01 | 0.02 ± 0.02 | 0.01 ± 0.01 | <LOQ | 0.01 ± 0.01 | <LOQ |
| ACE | 0.8 ± 0.3 | 1.7 ± 0.4 | 1.4 ± 0.1 | 2.6 ± 0.2 | 2.9 ± 0.3 | 2 ± 0.2 | 1.1 ± 0.2 | 0.09 ± 0.03 | <LOQ |
| DBF | 7 ± 1 | 10.2 ± 0.8 | 9.7 ± 0.6 | 10.8 ± 0.7 | 10.8 ± 0.6 | 9 ± 0.9 | 4.2 ± 0.5 | 0.25 ± 0.02 | <LOQ |
| FLU | 16 ± 3 | 21.6 ± 0.8 | 19.7 ± 0.6 | 22 ± 1 | 20.6 ± 0.4 | 16 ± 1 | 8.4 ± 0.6 | 0.4 ± 0.06 | 0.04 ± 0.06 |
| FLU-9et | 0.2 ± 0.07 | 0.58 ± 0.02 | 0.77 ± 0.02 | 1.4 ± 0.2 | 1.53 ± 0.04 | 1.4 ± 0.2 | 0.76 ± 0.05 | 0.21 ± 0.04 | 0.01 ± 0.01 |
| FLU-1 | 18 ± 6 | 36.8 ± 0.2 | 32.9 ± 0.9 | 25 ± 2 | 19.7 ± 0.2 | 11.3 ± 0.7 | 10 ± 0.5 | <LOQ | <LOQ |
| FLU-9pro | 0.01 ± 0.01 | 0.05 ± 0.03 | 0.17 ± 0.03 | 0.38 ± 0.04 | 0.49 ± 0.02 | 0.52 ± 0.09 | 0.26 ± 0.02 | 0.1 ± 0.03 | <LOQ |
| DBT | 14 ± 3 | 26 ± 1 | 22.9 ± 0.7 | 24 ± 2 | 21.2 ± 0.4 | 14 ± 2 | 8.1 ± 0.7 | 0.38 ± 0.07 | 0.01 ± 0.01 |
| DBT-4 | 8 ± 3 | 21 ± 1 | 19.6 ± 0.6 | 12 ± 2 | 8.6 ± 0.3 | 5 ± 0.7 | 5.2 ± 0.3 | 0.3 ± 0.1 | <LOQ |
| DBT-4et | 1.9 ± 0.8 | 5.6 ± 0.3 | 5.4 ± 0.2 | 2.4 ± 0.4 | 2 ± 0.1 | 1.7 ± 0.5 | 1.8 ± 0.3 | 0.17 ± 0.05 | 0.03 ± 0.01 |
| DBT-4pro | 1 ± 0.4 | 2.67 ± 0.07 | 2.9 ± 0.2 | 1.8 ± 0.2 | 1.6 ± 0.1 | 1.6 ± 0.7 | 1.7 ± 0.4 | 0.14 ± 0.07 | 0.07 ± 0.03 |
| DBT-4but | 0.4 ± 0.1 | 0.89 ± 0.06 | 0.8 ± 0.1 | 0.48 ± 0.07 | 0.66 ± 0.07 | 1 ± 0.7 | 1.1 ± 0.4 | 0.05 ± 0.03 | 0.1 ± 0.05 |
| PHE | 27 ± 7 | 49.1 ± 0.7 | 45 ± 2 | 40 ± 4 | 31.8 ± 0.8 | 17.6 ± 0.8 | 13.7 ± 0.5 | 0.7 ± 0.3 | 0.05 ± 0.09 |
| ANT | <LOQ | <LOQ | <LOQ | <LOQ | <LOQ | <LOQ | <LOQ | <LOQ | <LOQ |
| PHE-3 | 13 ± 5 | 31.8 ± 0.4 | 25 ± 1 | 9 ± 2 | 7.3 ± 0.3 | 5 ± 1 | 5.7 ± 0.8 | 0.16 ± 0.06 | <LOQ |
| PHE-2 | 13 ± 5 | 30.7 ± 0.6 | 22 ± 1 | 8 ± 2 | 6.2 ± 0.4 | 4 ± 1 | 5 ± 1 | 0.2 ± 0.1 | <LOQ |
| PHE-9 | 17 ± 7 | 42.3 ± 0.6 | 42 ± 2 | 25 ± 3 | 18.9 ± 0.7 | 13 ± 2 | 12.2 ± 0.6 | 1.1 ± 0.3 | 0.06 ± 0.01 |
| PHE-1 | 13 ± 5 | 31.5 ± 0.2 | 22 ± 1 | 8 ± 1 | 6.3 ± 0.3 | 4.8 ± 0.9 | 5.9 ± 0.8 | 0.21 ± 0.06 | <LOQ |
| PHE-3,6 | 5 ± 2 | 13.4 ± 0.2 | 10.4 ± 0.7 | 4.2 ± 0.5 | 4.1 ± 0.3 | 4 ± 1 | 4.3 ± 0.9 | 0.3 ± 0.2 | 0.04 ± 0.02 |
| PHE-1,7 | 7 ± 3 | 14.1 ± 0.4 | 5 ± 1 | 1.9 ± 0.1 | 2.8 ± 0.6 | 4 ± 2 | 4.6 ± 0.8 | 0.05 ± 0.03 | 0.01 ± 0.02 |
| PHE-1,2 | 1.4 ± 0.6 | 2.94 ± 0.03 | 0.9 ± 0.1 | 0.37 ± 0.03 | 0.46 ± 0.01 | 0.5 ± 0.3 | 0.7 ± 0.2 | <LOQ | <LOQ |
| PHE-2,6,9 | 3 ± 1 | 8.8 ± 0.9 | 3 ± 0.7 | 2 ± 0.4 | 3 ± 0.6 | 5 ± 2 | 6 ± 1 | 0.18 ± 0.08 | 0.15 ± 0.09 |
| PHE-1,2,6 | 0.6 ± 0.3 | 1.5 ± 0.1 | 0.4 ± 0.1 | 0.26 ± 0.06 | 0.42 ± 0.11 | 0.6 ± 0.3 | 0.8 ± 0.2 | <LOQ | <LOQ |
| PHE-1,2,7 | 0.2 ± 0.1 | 0.55 ± 0.04 | 0.19 ± 0.05 | 0.13 ± 0.03 | 0.18 ± 0.04 | 0.2 ± 0.1 | 0.28 ± 0.08 | 0.01 ± 0.01 | <LOQ |
| PHE-1,2,6,9 | 0.17 ± 0.07 | 0.49 ± 0.08 | 0.18 ± 0.03 | 0.09 ± 0.01 | 0.16 ± 0.06 | 0.3 ± 0.2 | 0.36 ± 0.07 | <LOQ | <LOQ |
| FLA | 2.1 ± 0.9 | 5.7 ± 0.2 | 4.61 ± 0.08 | 1.52 ± 0.1 | 1.2 ± 0.2 | 0.9 ± 0.4 | 1 ± 0.2 | 0.04 ± 0.03 | <LOQ |
| PYR | 2.4 ± 0.9 | 5.6 ± 0.2 | 3 ± 0.2 | 0.97 ± 0.12 | 1.13 ± 0.06 | 1.2 ± 0.4 | 1.1 ± 0.2 | 0.18 ± 0.02 | <LOQ |
| FLA-2 | 0.5 ± 0.3 | 1.58 ± 0.08 | 0.7 ± 0.1 | 0.27 ± 0.09 | 0.38 ± 0.08 | <LOQ | 0.6 ± 0.1 | <LOQ | <LOQ |
| PYR-1 | 0.7 ± 0.3 | 1.8 ± 0.1 | 0.55 ± 0.08 | 0.26 ± 0.05 | 0.38 ± 0.09 | 0.6 ± 0.3 | 0.7 ± 0.1 | 0.08 ± 0.02 | 0.05 ± 0.01 |
| PYR-4,5 | <LOQ | <LOQ | <LOQ | <LOQ | <LOQ | <LOQ | <LOQ | <LOQ | <LOQ |
| PYR-1pro | <LOQ | <LOQ | <LOQ | <LOQ | <LOQ | <LOQ | <LOQ | <LOQ | <LOQ |
| PYR-1et | <LOQ | <LOQ | <LOQ | <LOQ | <LOQ | 0.09 ± 0.06 | <LOQ | <LOQ | <LOQ |
| PYR-1but | <LOQ | <LOQ | <LOQ | <LOQ | <LOQ | <LOQ | <LOQ | <LOQ | <LOQ |
| BAA | 0.28 ± 0.1 | 3 ± 4 | 4.9 ± 0.5 | 2.6 ± 0.5 | 2.7 ± 0.5 | 0.2 ± 0.1 | 1 ± 1 | 0.37 ± 0.07 | 0.26 ± 0.04 |
| CHR | 1.66 ± 0.92 | 5.2 ± 0.2 | 3.7 ± 0.3 | 1.6 ± 0.2 | 1.6 ± 0.3 | 2.1 ± 0.7 | 1.9 ± 0.4 | 0.08 ± 0.06 | 0.01 ± 0.01 |
| CHR-1 | 0.19 ± 0.07 | 0.36 ± 0.02 | 0.13 ± 0.05 | 0.09 ± 0.03 | 0.16 ± 0.02 | 0.4 ± 0.3 | 0.3 ± 0.1 | <LOQ | <LOQ |
| CHR-6et | <LOQ | <LOQ | <LOQ | <LOQ | <LOQ | <LOQ | <LOQ | <LOQ | <LOQ |
| CHR-6pro | <LOQ | <LOQ | <LOQ | <LOQ | <LOQ | <LOQ | <LOQ | <LOQ | <LOQ |
| CHR-6but | <LOQ | <LOQ | <LOQ | <LOQ | <LOQ | <LOQ | <LOQ | <LOQ | <LOQ |
| BBF | 0.34 ± 0.08 | 0.46 ± 0.05 | 0.21 ± 0.06 | 0.14 ± 0.04 | 0.31 ± 0.04 | 0.6 ± 0.3 | 0.5 ± 0.2 | <LOQ | <LOQ |
| BKF | 0.17 ± 0.05 | 0.3 ± 0.01 | 0.13 ± 0.04 | 0.08 ± 0.02 | 0.17 ± 0.04 | 0.3 ± 0.1 | 0.3 ± 0.09 | <LOQ | <LOQ |
| BEP | 0.52 ± 0.18 | 0.82 ± 0.08 | 0.45 ± 0.04 | 0.21 ± 0.06 | 0.4 ± 0.02 | 0.6 ± 0.3 | 0.7 ± 0.3 | <LOQ | <LOQ |
| BAP | <LOQ | <LOQ | <LOQ | <LOQ | <LOQ | <LOQ | <LOQ | <LOQ | <LOQ |
| PER | 0.11 ± 0.04 | 0.14 ± 0.01 | 0.09 ± 0.03 | 0.04 ± 0.03 | 0.1 ± 0.01 | 0.03 ± 0.05 | 0.1 ± 0.09 | <LOQ | <LOQ |
| IND | 0.05 ± 0.09 | <LOQ | <LOQ | <LOQ | <LOQ | <LOQ | <LOQ | <LOQ | <LOQ |
| DBA | 0.16 ± 0.07 | 0.14 ± 0.03 | 0.15 ± 0.02 | 0.13 ± 0.02 | 0.17 ± 0.02 | <LOQ | <LOQ | <LOQ | <LOQ |
| BGP | 1 ± 1 | 0.19 ± 0.14 | 0.11 ± 0.03 | 0.1 ± 0.01 | 0.09 ± 0.02 | 0.2 ± 0.2 | 0.25 ± 0.08 | <LOQ | <LOQ |
| C1-BT | NA | NA | 0.21 ± 0.02 | NA | NA | 0.22 ± 0.01 | 0.12 ± 0.02 | NA | NA |
| C2-BT | NA | NA | 2.1 ± 0.1 | NA | NA | 2.3 ± 0.1 | 0.8 ± 0.3 | NA | NA |
| C3-BT | NA | NA | 9.7 ± 0.1 | NA | NA | 6.7 ± 0.9 | 4 ± 0.3 | NA | NA |
| C4-BT | NA | NA | 6.3 ± 0.2 | NA | NA | 4.4 ± 0.6 | 3.5 ± 0.1 | NA | NA |
| C1-NAP | NA | NA | 72 ± 5 | NA | NA | 65.2 ± 4.7 | 27.8 ± 2.9 | NA | NA |
| C2-NAP | NA | NA | 228 ± 7 | NA | NA | 186.9 ± 8 | 62.9 ± 51.9 | NA | NA |
| C3-NAP | NA | NA | 332 ± 5 | NA | NA | 189 ± 14.8 | 76.8 ± 57.9 | NA | NA |
| C4-NAP | NA | NA | 151 ± 2 | NA | NA | 93.5 ± 11.5 | 46.8 ± 29.4 | NA | NA |
| C1-FLU | NA | NA | 62.9 ± 0.8 | NA | NA | 18.6 ± 2.6 | 15.6 ± 0.5 | NA | NA |
| C2-FLU | NA | NA | 48.9 ± 0.3 | NA | NA | 19 ± 2.9 | 21.2 ± 2.7 | NA | NA |
| C3-FLU | NA | NA | 23 ± 2 | NA | NA | 17.8 ± 5 | 19.9 ± 3.4 | NA | NA |
| C1-DBT | NA | NA | 39.16 ± 0.03 | NA | NA | 10.6 ± 1.8 | 6.4 ± 5.1 | NA | NA |
| C2-DBT | NA | NA | 67 ± 3 | NA | NA | 22.5 ± 8.2 | 16.7 ± 12.9 | NA | NA |
| C3-DBT | NA | NA | 27 ± 4 | NA | NA | 20.5 ± 10.9 | 14.6 ± 11.8 | NA | NA |
| C4-DBT | NA | NA | 19 ± 4 | NA | NA | 26.7 ± 15.2 | 17.5 ± 14.3 | NA | NA |
| C1-PHE | NA | NA | 110 ± 5 | NA | NA | 26.7 ± 4.5 | 29.1 ± 3.1 | NA | NA |
| C2-PHE | NA | NA | 91 ± 8 | NA | NA | 40.4 ± 15.9 | 49.4 ± 12.6 | NA | NA |
| C3-PHE | NA | NA | 38 ± 6 | NA | NA | 34.8 ± 17.6 | 42.8 ± 13.5 | NA | NA |
| C4-PHE | NA | NA | 28 ± 6 | NA | NA | 38.9 ± 22.7 | 53.4 ± 16.5 | NA | NA |
| C1-PYR | NA | NA | 17 ± 2 | NA | NA | 11.8 ± 4.3 | 8.3 ± 5.5 | NA | NA |
| C2-PYR | NA | NA | 14 ± 4 | NA | NA | 16.5 ± 9.2 | 10.7 ± 8.3 | NA | NA |
| C3-PYR | NA | NA | 13 ± 4 | NA | NA | 21.9 ± 13.2 | 14.5 ± 12.1 | NA | NA |
| C1-CHR | NA | NA | 4 ± 1 | NA | NA | 6.3 ± 3.9 | 8.6 ± 3.4 | NA | NA |
| C2-CHR | NA | NA | 2.8 ± 0.8 | NA | NA | 7.3 ± 1.8 | 0.06 ± 0.06 | NA | NA |
| C3-CHR | NA | NA | 3 ± 1 | NA | NA | 9.7 ± 5.5 | 0.9 ± 0.5 | NA | NA |
| C4-CHR | NA | NA | 3.1 ± 0.9 | NA | NA | 16.3 ± 5.5 | 9 ± 2.1 | NA | NA |
| ΣPAH | 407 ± 111 | 768 ± 10 | 738 ± 21 | 600 ± 42 | 574 ± 17 | 429 ± 26 | 271 ± 7 | 13 ± 3 | 3 ± 1 |
| tPAH |  |  | 1560 ± 41 |  |  | 1006 ± 193 | 614 ± 252 |  |  |

Table S7 continued.

| **Body burden (pg/embryo)** | **Cod 9.1 µg/L 12 hrs** | **Cod 9.1 µg/L Day 1** | **Cod 9.1 µg/L Day 2** | **Cod 9.1 µg/L Day 3** | **Cod 9.1 µg/L Day 5** | **Cod 9.1 µg/L Day 7** | **Cod 9.1 µg/L Day 9** | **Cod 9.1 µg/L Day 11** | **Cod 9.1 µg/L Day 13** |
| --- | --- | --- | --- | --- | --- | --- | --- | --- | --- |
| BIP | 41 ± 3 | 69 ± 6 | 68 ± 4 | 66 ± 4 | 37.3 ± 0.9 | 49 ± 5 | 40 ± 1 | 19 ± 3 | 0.2 ± 0.2 |
| BT | <LOQ | <LOQ | <LOQ | <LOQ | <LOQ | <LOQ | <LOQ | <LOQ | <LOQ |
| BT-2,5 | 0.97 ± 0.06 | 1.6 ± 0.1 | 2.08 ± 0.08 | 1.9 ± 0.1 | 1.11 ± 0.04 | 1.3 ± 0.2 | 1.08 ± 0.03 | 0.5 ± 0.06 | <LOQ |
| BT-2,5,7 | 0.8 ± 0.1 | 1.3 ± 0.1 | 3.12 ± 0.09 | 3.5 ± 0.4 | 2.9 ± 0.1 | 2.4 ± 0.3 | 1.7 ± 0.1 | 0.84 ± 0.04 | <LOQ |
| NAP | 15 ± 1 | 19 ± 2 | 18 ± 1 | 19 ± 1 | 7.6 ± 0.4 | 11.7 ± 0.8 | 9.3 ± 0.6 | 3 ± 0.3 | <LOQ |
| NAP-2 | 98 ± 6 | 143 ± 9 | 129 ± 10 | 139 ± 9 | 66 ± 1 | 101 ± 17 | 85 ± 2 | 36 ± 5 | 0.3 ± 0.3 |
| NAP-1 | 37 ± 1 | 59 ± 5 | 57 ± 3 | 61 ± 5 | 29 ± 0.6 | 43 ± 9 | 36 ± 1 | 15 ± 2 | 0.1 ± 0.1 |
| NAP-2,6&2,7 | 101 ± 10 | 148 ± 14 | 203 ± 7 | 197 ± 12 | 122 ± 4 | 130 ± 19 | 91 ± 3 | 49 ± 5 | 0.4 ± 0.2 |
| NAP-1,4 | 63 ± 5 | 100 ± 10 | 156 ± 5 | 153 ± 12 | 101 ± 2 | 112 ± 17 | 84 ± 4 | 44 ± 5 | 0.3 ± 0.1 |
| NAP-1,3&2,3 | 47 ± 3 | 75 ± 8 | 107 ± 3 | 105 ± 8 | 67 ± 2 | 74 ± 10 | 57 ± 2 | 30 ± 3 | 0.6 ± 0.2 |
| NAP-1,3,7 | 20 ± 1 | 30 ± 4 | 69.9 ± 0.8 | 75 ± 3 | 38 ± 2 | 22 ± 1 | 16.2 ± 0.5 | 11 ± 1 | 0.05 ± 0.02 |
| NAP-2,3,5 | 66 ± 4 | 100 ± 11 | 231 ± 5 | 245 ± 14 | 181 ± 9 | 120 ± 8 | 80 ± 3 | 47 ± 5 | 0.78 ± 0.04 |
| NAP-1,2,3 | 2.7 ± 0.1 | 4.5 ± 0.6 | 12.1 ± 0.2 | 13 ± 2 | 10.1 ± 0.6 | 6.6 ± 0.4 | 4.9 ± 0.3 | 2.6 ± 0.3 | 0.03 ± 0.02 |
| NAP-1,2,5,6 | 3.9 ± 0.4 | 6 ± 1 | 18.3 ± 0.4 | 22 ± 2 | 15 ± 1 | 8.8 ± 0.8 | 6.2 ± 0.6 | 4.7 ± 0.7 | 0.01 ± 0.01 |
| NAP-1,4,6,7 | 7.3 ± 0.5 | 12 ± 2 | 32.2 ± 0.8 | 31 ± 2 | 12 ± 1 | 8 ± 2 | 5.2 ± 0.5 | 5.4 ± 0.9 | <LOQ |
| ACY | 0.07 ± 0.03 | 0.07 ± 0.03 | 0.02 ± 0.03 | 0.06 ± 0.06 | 0.03 ± 0.02 | 0.08 ± 0.05 | 0.04 ± 0.01 | 0.04 ± 0.01 | 0.01 ± 0.01 |
| ACE | 1.9 ± 0.1 | 3.5 ± 0.8 | 6.2 ± 0.7 | 5.3 ± 0.8 | 3.8 ± 0.7 | 3.8 ± 0.8 | 3.1 ± 0.3 | 1.6 ± 0.4 | <LOQ |
| DBF | 17.3 ± 0.6 | 33 ± 2 | 34 ± 2 | 32 ± 3 | 17 ± 0.6 | 20 ± 2 | 14.5 ± 0.6 | 8 ± 0.8 | <LOQ |
| FLU | 38 ± 3 | 71 ± 8 | 73 ± 3 | 67 ± 6 | 35 ± 2 | 36 ± 4 | 23 ± 2 | 16 ± 1 | 0.1 ± 0.1 |
| FLU-9et | 0.35 ± 0.02 | 0.6 ± 0.1 | 1.85 ± 0.04 | 2.5 ± 0.3 | 3.3 ± 0.2 | 2.8 ± 0.1 | 2.35 ± 0.09 | 1.28 ± 0.08 | 0.04 ± 0.01 |
| FLU-1 | 29 ± 2 | 47 ± 4 | 99 ± 3 | 91 ± 4 | 46 ± 3 | 28 ± 4 | 18 ± 1 | 16.2 ± 0.8 | 0.26 ± 0.05 |
| FLU-9pro | 0.02 ± 0.02 | 0.08 ± 0.08 | 0.26 ± 0.02 | 0.5 ± 0.1 | 0.72 ± 0.04 | 0.88 ± 0.04 | 0.84 ± 0.06 | 0.47 ± 0.04 | 0.01 ± 0.01 |
| DBT | 24 ± 2 | 45 ± 5 | 75 ± 1 | 75 ± 9 | 47 ± 3 | 33 ± 4 | 20 ± 1 | 14.5 ± 0.7 | 0.03 ± 0.04 |
| DBT-4 | 9.6 ± 0.6 | 17 ± 2 | 43 ± 1 | 45 ± 2 | 23 ± 2 | 13 ± 2 | 7.8 ± 0.7 | 8 ± 1 | 0.05 ± 0.04 |
| DBT-4et | 2 ± 0.1 | 4 ± 1 | 10.5 ± 0.4 | 11 ± 1 | 4.9 ± 0.4 | 3 ± 1 | 2.4 ± 0.4 | 3 ± 0.7 | 0.02 ± 0.01 |
| DBT-4pro | 0.78 ± 0.07 | 2 ± 1 | 4.2 ± 0.5 | 5 ± 1 | 2.8 ± 0.2 | 2 ± 1 | 1.7 ± 0.5 | 2.5 ± 0.7 | 0.02 ± 0.01 |
| DBT-4but | 0.27 ± 0.05 | 1.1 ± 0.9 | 1.5 ± 0.4 | 2 ± 1 | 0.69 ± 0.06 | 1 ± 1 | 0.9 ± 0.4 | 1.5 ± 0.4 | 0.05 ± 0.02 |
| PHE | 42 ± 4 | 70 ± 7 | 123 ± 6 | 119 ± 1 | 73 ± 4 | 44 ± 5 | 26.9 ± 0.6 | 22 ± 2 | <LOQ |
| ANT | <LOQ | <LOQ | <LOQ | <LOQ | <LOQ | <LOQ | <LOQ | <LOQ | <LOQ |
| PHE-3 | 17 ± 1 | 28 ± 4 | 71 ± 3 | 64 ± 3 | 21 ± 2 | 14 ± 5 | 9 ± 1 | 11 ± 2 | 0.01 ± 0.01 |
| PHE-2 | 16 ± 1 | 28 ± 4 | 67 ± 3 | 58 ± 2 | 17 ± 2 | 12 ± 5 | 8 ± 1 | 10 ± 2 | <LOQ |
| PHE-9 | 21 ± 2 | 37 ± 5 | 89 ± 4 | 94 ± 2 | 50 ± 4 | 31 ± 7 | 20 ± 2 | 22 ± 3 | 0.2 ± 0.1 |
| PHE-1 | 16 ± 1 | 29 ± 4 | 64 ± 2 | 55 ± 2 | 16 ± 1 | 13 ± 5 | 8 ± 1 | 10 ± 2 | 0.01 ± 0.02 |
| PHE-3,6 | 4.9 ± 0.2 | 9 ± 3 | 23 ± 1 | 21 ± 3 | 7.5 ± 0.5 | 8 ± 5 | 6 ± 1 | 7 ± 2 | 0.07 ± 0.02 |
| PHE-1,7 | 6.1 ± 0.3 | 11 ± 3 | 24 ± 2 | 11 ± 4 | 3.6 ± 0.5 | 7 ± 6 | 6 ± 2 | 6 ± 2 | 0.05 ± 0.01 |
| PHE-1,2 | 1.32 ± 0.07 | 2.3 ± 0.6 | 5.8 ± 0.3 | 2.6 ± 0.5 | 0.7 ± 0.1 | 1 ± 1 | 0.8 ± 0.2 | 1.2 ± 0.4 | <LOQ |
| PHE-2,6,9 | 3.4 ± 0.6 | 9 ± 4 | 17 ± 2 | 10 ± 7 | 3 ± 0.4 | 8 ± 8 | 5 ± 2 | 8 ± 3 | <LOQ |
| PHE-1,2,6 | 0.5 ± 0.1 | 1.3 ± 0.5 | 2.6 ± 0.3 | 1.3 ± 0.9 | 0.36 ± 0.05 | 1 ± 1 | 0.6 ± 0.2 | 1 ± 0.4 | <LOQ |
| PHE-1,2,7 | 0.21 ± 0.03 | 0.5 ± 0.2 | 1 ± 0.1 | 0.5 ± 0.3 | 0.19 ± 0.03 | 0.5 ± 0.4 | 0.25 ± 0.09 | 0.4 ± 0.1 | <LOQ |
| PHE-1,2,6,9 | 0.13 ± 0.02 | 0.6 ± 0.5 | 0.9 ± 0.2 | 0.6 ± 0.5 | 0.12 ± 0.02 | 0.5 ± 0.6 | 0.3 ± 0.2 | 0.5 ± 0.2 | <LOQ |
| FLA | 3 ± 0.9 | 4.2 ± 0.9 | 11.7 ± 0.5 | 10.7 ± 0.9 | 3.4 ± 0.2 | 3 ± 1 | 1.8 ± 0.4 | 2 ± 0.4 | <LOQ |
| PYR | 3.1 ± 0.9 | 5 ± 1 | 12.2 ± 0.3 | 8 ± 1 | 2.2 ± 0.2 | 3 ± 1 | 2 ± 0.3 | 2.3 ± 0.5 | <LOQ |
| FLA-2 | 0.53 ± 0.04 | 1.2 ± 0.4 | 2.8 ± 0.2 | 1.7 ± 0.6 | 0.43 ± 0.05 | 0.9 ± 0.8 | <LOQ | 0.9 ± 0.3 | <LOQ |
| PYR-1 | 0.75 ± 0.09 | 1.7 ± 0.6 | 3.3 ± 0.2 | 1.8 ± 0.3 | 0.4 ± 0.1 | 1 ± 1 | 0.6 ± 0.2 | 0.9 ± 0.3 | 0.05 ± 0.01 |
| PYR-4,5 | <LOQ | <LOQ | <LOQ | <LOQ | <LOQ | <LOQ | <LOQ | <LOQ | <LOQ |
| PYR-1pro | <LOQ | <LOQ | <LOQ | <LOQ | <LOQ | <LOQ | <LOQ | <LOQ | <LOQ |
| PYR-1et | <LOQ | 0.14 ± 0.09 | 0.22 ± 0.07 | 0.2 ± 0.2 | 0.08 ± 0.01 | 0.2 ± 0.2 | 0.14 ± 0.08 | 0.25 ± 0.03 | <LOQ |
| PYR-1but | <LOQ | <LOQ | <LOQ | <LOQ | <LOQ | <LOQ | <LOQ | <LOQ | <LOQ |
| BAA | 0.4 ± 0.1 | 0.6 ± 0.2 | 1.3 ± 0.1 | 0.5 ± 0.2 | 0.2 ± 0.01 | 0.4 ± 0.4 | 0.28 ± 0.09 | 0.4 ± 0.2 | 0.06 ± 0.01 |
| CHR | 1.7 ± 0.2 | 4 ± 1 | 9 ± 1 | 8 ± 2 | 2.9 ± 0.5 | 4 ± 4 | 2.5 ± 0.7 | 4 ± 1 | <LOQ |
| CHR-1 | 0.14 ± 0.01 | 0.4 ± 0.3 | 0.7 ± 0.2 | 0.4 ± 0.4 | 0.17 ± 0.04 | 0.5 ± 0.6 | 0.3 ± 0.1 | 0.4 ± 0.1 | <LOQ |
| CHR-6et | <LOQ | <LOQ | <LOQ | <LOQ | <LOQ | <LOQ | <LOQ | <LOQ | <LOQ |
| CHR-6pro | <LOQ | <LOQ | <LOQ | <LOQ | <LOQ | <LOQ | <LOQ | <LOQ | <LOQ |
| CHR-6but | <LOQ | <LOQ | <LOQ | <LOQ | <LOQ | <LOQ | <LOQ | <LOQ | <LOQ |
| BBF | 0.6 ± 0.5 | 0.6 ± 0.4 | 0.9 ± 0.2 | 0.6 ± 0.6 | 0.23 ± 0.04 | 0.8 ± 0.9 | 0.5 ± 0.2 | 0.7 ± 0.2 | 0.09 ± 0.01 |
| BKF | 0.4 ± 0.5 | 0.4 ± 0.2 | 0.5 ± 0.1 | 0.3 ± 0.3 | 0.1 ± 0.01 | 0.3 ± 0.4 | 0.2 ± 0.09 | 0.4 ± 0.1 | <LOQ |
| BEP | 0.6 ± 0.4 | 1 ± 0.6 | 1.4 ± 0.4 | 1.1 ± 0.8 | 0.33 ± 0.05 | 1 ± 1 | 0.6 ± 0.2 | 0.9 ± 0.4 | <LOQ |
| BAP | 0.1 ± 0.1 | 0.2 ± 0.2 | 0.37 ± 0.09 | 0.3 ± 0.4 | 0.03 ± 0.04 | 0.2 ± 0.4 | <LOQ | <LOQ | <LOQ |
| PER | 0.04 ± 0.03 | 0.1 ± 0.1 | 0.17 ± 0.06 | 0.1 ± 0.1 | 0.02 ± 0.02 | 0.2 ± 0.3 | 0.05 ± 0.04 | 0.06 ± 0.06 | <LOQ |
| IND | 0.1 ± 0.2 | <LOQ | <LOQ | <LOQ | <LOQ | <LOQ | <LOQ | <LOQ | <LOQ |
| DBA | 0.04 ± 0.07 | <LOQ | 0.1 ± 0.02 | 0.1 ± 0.2 | 0 ± 0.01 | 0.2 ± 0.2 | <LOQ | <LOQ | <LOQ |
| BGP | 0.4 ± 0.5 | 0.2 ± 0.3 | 0.2 ± 0.1 | 0.3 ± 0.4 | 0.04 ± 0.03 | 0.3 ± 0.4 | 0.2 ± 0.1 | 0.1 ± 0.1 | <LOQ |
| C1-BT | NA | NA | NA | 0.6 ± 0 | NA | NA | 0.45 ± 0.02 | 0.26 ± 0.04 | NA |
| C2-BT | NA | NA | NA | 8.1 ± 0.5 | NA | NA | 4.7 ± 0.2 | 1.8 ± 0.3 | NA |
| C3-BT | NA | NA | NA | 26.2 ± 0.6 | NA | NA | 12.3 ± 0.1 | 7.2 ± 0.4 | NA |
| C4-BT | NA | NA | NA | 18.7 ± 0.6 | NA | NA | 8 ± 0.4 | 5.9 ± 0.5 | NA |
| C1-NAP | NA | NA | NA | 200 ± 15 | NA | NA | 121 ± 3 | 51 ± 7 | NA |
| C2-NAP | NA | NA | NA | 708 ± 45 | NA | NA | 361 ± 11 | 169 ± 18 | NA |
| C3-NAP | NA | NA | NA | 1050 ± 95 | NA | NA | 322 ± 19 | 194 ± 20 | NA |
| C4-NAP | NA | NA | NA | 465 ± 69 | NA | NA | 160 ± 23 | 107 ± 10 | NA |
| C1-FLU | NA | NA | NA | 211 ± 15 | NA | NA | 30 ± 3 | 33 ± 3 | NA |
| C2-FLU | NA | NA | NA | 140 ± 17 | NA | NA | 31 ± 2 | 36 ± 5 | NA |
| C3-FLU | NA | NA | NA | 50 ± 14 | NA | NA | 26 ± 4 | 30 ± 3 | NA |
| C1-DBT | NA | NA | NA | 117 ± 6 | NA | NA | 17 ± 1 | 18 ± 3 | NA |
| C2-DBT | NA | NA | NA | 150 ± 22 | NA | NA | 32 ± 5 | 46 ± 12 | NA |
| C3-DBT | NA | NA | NA | 56 ± 25 | NA | NA | 23 ± 8 | 37 ± 12 | NA |
| C4-DBT | NA | NA | NA | 44 ± 34 | NA | NA | 24 ± 9 | 42 ± 14 | NA |
| C1-PHE | NA | NA | NA | 271 ± 8 | NA | NA | 45 ± 5 | 53 ± 9 | NA |
| C2-PHE | NA | NA | NA | 228 ± 52 | NA | NA | 61 ± 14 | 84 ± 26 | NA |
| C3-PHE | NA | NA | NA | 82 ± 41 | NA | NA | 39 ± 13 | 64 ± 21 | NA |
| C4-PHE | NA | NA | NA | 67 ± 50 | NA | NA | 38 ± 13 | 74 ± 23 | NA |
| C1-PYR | NA | NA | NA | 38 ± 11 | NA | NA | 16 ± 4 | 22 ± 6 | NA |
| C2-PYR | NA | NA | NA | 32 ± 21 | NA | NA | 18 ± 7 | 29 ± 11 | NA |
| C3-PYR | NA | NA | NA | 34 ± 33 | NA | NA | 21 ± 10 | 38 ± 14 | NA |
| C1-CHR | NA | NA | NA | 10 ± 8 | NA | NA | 6 ± 3 | 15 ± 6 | NA |
| C2-CHR | NA | NA | NA | 8 ± 8 | NA | NA | 6 ± 1 | 0.03 ± 0.06 | NA |
| C3-CHR | NA | NA | NA | 12 ± 13 | NA | NA | 7 ± 4 | 1.2 ± 0.9 | NA |
| C4-CHR | NA | NA | NA | 18 ± 22 | NA | NA | 14 ± 3 | 12 ± 4 | NA |
| ΣPAH | 768 ± 48 | 1237 ± 129 | 1990 ± 58 | 1939 ± 52 | 1077 ± 47 | 989 ± 91 | 711 ± 39 | 452 ± 52 | 4 ± 1 |
| tPAH |  |  |  | 4455 ± 558 |  |  | 1585 ± 153 | 1262 ± 216 |  |

Table S7 continued.

| **Bpdy burden (pg/embryo)** | **Haddock 0.09 µg/L Day 3** | **Haddock 0.09 µg/L Day 9** | **Haddock 0.10 µg/L Day 3** | **Haddock 0.10 µg/L Day 9** | **Haddock 0.17 µg/L Day 3** | **Haddock 0.17 µg/L Day 9** | **Haddock 0.21 µg/L Day 1** | **Haddock 0.21 µg/L Day 2** | **Haddock 0.21 µg/L Day 3** |
| --- | --- | --- | --- | --- | --- | --- | --- | --- | --- |
| BIP | 0.25 ± 0.05 | 0.29 ± 0.1 | 1.19 ± 0.11 | 0.89 ± 0.1 | 1.6 ± 0.4 | 1 ± 0.2 | 2.1 ± 0.1 | 2 ± 0.4 | 2.3 ± 0.5 |
| BT | <LOQ | 0.01 ± 0.01 | <LOQ | <LOQ | <LOQ | <LOQ | <LOQ | <LOQ | <LOQ |
| BT-2,5 | <LOQ | 0.03 ± 0 | <LOQ | 0.05 ± 0.01 | <LOQ | 0.06 ± 0 | <LOQ | <LOQ | <LOQ |
| BT-2,5,7 | <LOQ | 0.05 ± 0.01 | <LOQ | 0.07 ± 0 | 0.1 ± 0.01 | 0.1 ± 0.04 | 0.09 ± 0.01 | 0.12 ± 0.01 | 0.13 ± 0.03 |
| NAP | 0.1 ± 0.2 | <LOQ | 0.4 ± 0.09 | 0.5 ± 0.3 | 0.04 ± 0.07 | 0.4 ± 0.5 | 0.2 ± 0.07 | 3 ± 6 | 7 ± 6 |
| NAP-2 | 0.1 ± 0.1 | 0.31 ± 0.07 | 2.6 ± 0.1 | 2.44 ± 0.17 | 3 ± 0.05 | 2.6 ± 0.5 | 3.7 ± 0.2 | 3 ± 1 | 4.2 ± 0.7 |
| NAP-1 | <LOQ | <LOQ | 1.1 ± 0.1 | 0.85 ± 0.09 | 1.1 ± 0.2 | 0.9 ± 0.2 | 1.3 ± 0.2 | 1.3 ± 0.6 | 1.8 ± 0.4 |
| NAP-2,6&2,7 | 1.1 ± 0.1 | 0.47 ± 0.03 | 4.24 ± 0.08 | 3.4 ± 0.1 | 5.81 ± 0.04 | 3.9 ± 0.2 | 7.1 ± 0.3 | 7 ± 1 | 8.3 ± 0.4 |
| NAP-1,4 | 0.9 ± 0.1 | 0.49 ± 0.02 | 2.97 ± 0.06 | 3.13 ± 0.09 | 4 ± 0.2 | 3.3 ± 0.3 | 4.3 ± 0.2 | 5.1 ± 0.9 | 6 ± 0.4 |
| NAP-1,3&2,3 | 0.74 ± 0.09 | 0.55 ± 0.03 | 1.65 ± 0.04 | 2.01 ± 0.05 | 2.5 ± 0.2 | 2.1 ± 0.1 | 3 ± 0.2 | 3.2 ± 0.5 | 3.6 ± 0.2 |
| NAP-1,3,7 | 0.67 ± 0.05 | 0.12 ± 0.04 | 1.99 ± 0.03 | 1 ± 0.2 | 2.5 ± 0.2 | 1.02 ± 0.03 | 2.3 ± 0.1 | 3.3 ± 0.8 | 3.3 ± 0.2 |
| NAP-2,3,5 | 2.8 ± 0.3 | 1.63 ± 0.09 | 6.3 ± 0.3 | 4.26 ± 0.05 | 9 ± 1 | 4.9 ± 0.2 | 8 ± 0.3 | 11 ± 2 | 13.3 ± 0.6 |
| NAP-1,2,3 | 0.08 ± 0.02 | 0.03 ± 0.01 | 0.19 ± 0.06 | 0.19 ± 0.02 | 0.3 ± 0.05 | 0.14 ± 0.05 | 0.3 ± 0.01 | 0.5 ± 0.1 | 0.54 ± 0.07 |
| NAP-1,2,5,6 | 0.3 ± 0.2 | 0.03 ± 0.03 | 0.53 ± 0.04 | 0.37 ± 0.01 | 0.66 ± 0.09 | 0.43 ± 0.04 | 0.57 ± 0.07 | 1.2 ± 0.4 | 0.9 ± 0.07 |
| NAP-1,4,6,7 | 0.5 ± 0.6 | <LOQ | 0.47 ± 0.03 | 0.24 ± 0.01 | 0.6 ± 0.1 | 0.27 ± 0.02 | 0.92 ± 0.08 | 1.7 ± 0.7 | 0.8 ± 0.1 |
| ACY | <LOQ | <LOQ | <LOQ | <LOQ | <LOQ | <LOQ | <LOQ | <LOQ | <LOQ |
| ACE | <LOQ | <LOQ | 0.1 ± 0.02 | 0.07 ± 0.04 | 0.02 ± 0.03 | 0.11 ± 0.06 | 0.03 ± 0.04 | 0.1 ± 0.09 | 0.17 ± 0.02 |
| DBF | <LOQ | <LOQ | 0.38 ± 0.04 | 0.29 ± 0.07 | 0.1 ± 0.2 | 0.4 ± 0.2 | 0.8 ± 0.1 | 0.9 ± 0.4 | 1 ± 0.2 |
| FLU | <LOQ | <LOQ | 0.78 ± 0.09 | 0.8 ± 0.2 | 0.7 ± 0.3 | 0.9 ± 0.2 | 1.81 ± 0.08 | 1.8 ± 0.4 | 1.82 ± 0.02 |
| FLU-9et | 0.04 ± 0.04 | <LOQ | 0.02 ± 0.02 | 0.09 ± 0.01 | 0.07 ± 0.03 | 0.09 ± 0.02 | 0.03 ± 0.03 | 0.07 ± 0.06 | 0.09 ± 0.02 |
| FLU-1 | 0.8 ± 0.7 | 0.17 ± 0.04 | 1.9 ± 0.1 | 0.81 ± 0.09 | 2.4 ± 0.1 | 0.8 ± 0.2 | 3.4 ± 0.2 | 5 ± 1 | 4.5 ± 0.3 |
| FLU-9pro | <LOQ | <LOQ | <LOQ | 0.01 ± 0.01 | <LOQ | 0.01 ± 0.02 | <LOQ | 0.02 ± 0.03 | <LOQ |
| DBT | 0.2 ± 0.3 | <LOQ | 0.68 ± 0.03 | 0.51 ± 0.07 | 1.3 ± 0.6 | 0.7 ± 0.2 | 1.4 ± 0.1 | 2.2 ± 0.5 | 2.1 ± 0.2 |
| DBT-4 | 1 ± 1 | 0.03 ± 0.03 | 0.76 ± 0.03 | 0.37 ± 0.03 | 1.4 ± 0.2 | 0.48 ± 0.02 | 1.54 ± 0.08 | 3.5 ± 0.8 | 2.3 ± 0.3 |
| DBT-4et | 1 ± 1 | 0.11 ± 0.08 | 0.24 ± 0.01 | 0.12 ± 0.01 | 0.4 ± 0.09 | 0.17 ± 0.02 | 0.53 ± 0.03 | 1.7 ± 0.6 | 0.7 ± 0.2 |
| DBT-4pro | 1 ± 1 | 0.3 ± 0.1 | 0.18 ± 0.01 | 0.11 ± 0.01 | 0.34 ± 0.08 | 0.13 ± 0.01 | 0.38 ± 0.01 | 1.6 ± 0.8 | 0.6 ± 0.2 |
| DBT-4but | 1 ± 1 | 0.3 ± 0.2 | 0.05 ± 0.01 | 0.04 ± 0.01 | 0.08 ± 0.01 | 0.06 ± 0.02 | 0.22 ± 0 | 1.1 ± 0.6 | 0.4 ± 0.2 |
| PHE | 1 ± 1 | <LOQ | 2.1 ± 0.3 | 0.8 ± 0.1 | 3.2 ± 0.9 | 1 ± 0.2 | 5.1 ± 0.3 | 8 ± 1 | 7.3 ± 0.4 |
| ANT | <LOQ | <LOQ | <LOQ | <LOQ | <LOQ | <LOQ | <LOQ | <LOQ | <LOQ |
| PHE-3 | 2 ± 2 | 0.1 ± 0.1 | 0.66 ± 0.05 | 0.24 ± 0.05 | 0.9 ± 0.1 | 0.37 ± 0.08 | 2.14 ± 0.06 | 4 ± 1 | 1.4 ± 0.4 |
| PHE-2 | 2 ± 2 | 0.1 ± 0.1 | 0.7 ± 0.04 | 0.13 ± 0.04 | 0.6 ± 0.1 | 0.21 ± 0.05 | 2.4 ± 0.08 | 5 ± 1 | 1.5 ± 0.5 |
| PHE-9 | 3 ± 4 | 0.3 ± 0.3 | 1.85 ± 0.06 | 1.12 ± 0.09 | 3.11 ± 0.08 | 1.51 ± 0.07 | 3 ± 0.1 | 9 ± 2 | 5.7 ± 0.8 |
| PHE-1 | 2 ± 3 | 0.2 ± 0.2 | 0.62 ± 0.08 | 0.24 ± 0.02 | 1 ± 0.3 | 0.39 ± 0.03 | 2.38 ± 0.06 | 5 ± 1 | 1.6 ± 0.5 |
| PHE-3,6 | 2 ± 3 | 0.5 ± 0.3 | 0.38 ± 0.03 | 0.31 ± 0.03 | 0.65 ± 0.08 | 0.42 ± 0.04 | 1.15 ± 0.02 | 4 ± 1 | 1.3 ± 0.6 |
| PHE-1,7 | 3 ± 4 | 1 ± 0.5 | 0.07 ± 0.03 | 0.2 ± 0.03 | 0.4 ± 0.3 | 0.3 ± 0.03 | 1.14 ± 0.03 | 4 ± 2 | 1 ± 0.7 |
| PHE-1,2 | 0.5 ± 0.8 | 0.14 ± 0.06 | 0.02 ± 0.01 | 0.03 ± 0.01 | 0.08 ± 0.06 | 0.03 ± 0.01 | 0.23 ± 0.02 | 0.7 ± 0.3 | 0.2 ± 0.2 |
| PHE-2,6,9 | 4 ± 6 | 1.5 ± 0.7 | 0.16 ± 0.04 | 0.12 ± 0.02 | 0.3 ± 0.3 | 0.2 ± 0.02 | 0.88 ± 0.04 | 5 ± 3 | 2 ± 1 |
| PHE-1,2,6 | 1 ± 1 | 0.2 ± 0.1 | <LOQ | 0 ± 0.01 | <LOQ | 0 ± 0.01 | 0.11 ± 0.03 | 0.8 ± 0.4 | 0.2 ± 0.2 |
| PHE-1,2,7 | 0.2 ± 0.3 | 0.06 ± 0.03 | <LOQ | <LOQ | <LOQ | <LOQ | <LOQ | 0.2 ± 0.2 | 0.03 ± 0.06 |
| PHE-1,2,6,9 | 0.3 ± 0.5 | 0.08 ± 0.08 | <LOQ | <LOQ | <LOQ | <LOQ | 0.04 ± 0.02 | 0.4 ± 0.3 | 0.15 ± 0.08 |
| FLA | 1 ± 0.7 | 0.13 ± 0.03 | 0.47 ± 0.06 | 0.02 ± 0.01 | 0.4 ± 0.08 | 0.09 ± 0.06 | 1.06 ± 0.07 | 1.4 ± 0.3 | 0.5 ± 0.1 |
| PYR | 1 ± 1 | 0.07 ± 0.09 | 0.15 ± 0.04 | <LOQ | 0.2 ± 0.4 | 0.01 ± 0.01 | 0.55 ± 0.06 | 1.1 ± 0.4 | 0.3 ± 0.2 |
| FLA-2 | 0.3 ± 0.6 | <LOQ | <LOQ | <LOQ | <LOQ | <LOQ | 0.06 ± 0.03 | 0.4 ± 0.3 | 0.05 ± 0.08 |
| PYR-1 | 0.5 ± 0.8 | 0.13 ± 0.06 | <LOQ | <LOQ | 0.3 ± 0.5 | 0.03 ± 0.01 | 0.12 ± 0.02 | 0.6 ± 0.3 | 0.2 ± 0.1 |
| PYR-4,5 | 1 ± 1 | <LOQ | <LOQ | <LOQ | 0.03 ± 0.05 | <LOQ | 0.3 ± 0.1 | 1.2 ± 0.7 | 0.4 ± 0.2 |
| PYR-1pro | <LOQ | <LOQ | <LOQ | <LOQ | <LOQ | <LOQ | <LOQ | <LOQ | <LOQ |
| PYR-1et | 0.1 ± 0.2 | 0.02 ± 0.03 | <LOQ | <LOQ | <LOQ | <LOQ | <LOQ | 0.1 ± 0.2 | <LOQ |
| PYR-1but | 0.03 ± 0.06 | <LOQ | <LOQ | <LOQ | 0.04 ± 0.07 | <LOQ | <LOQ | 0.04 ± 0.06 | <LOQ |
| BAA | 0.2 ± 0.3 | <LOQ | <LOQ | 0.03 ± 0.05 | <LOQ | <LOQ | <LOQ | 0.1 ± 0.1 | <LOQ |
| CHR | 1 ± 2 | 0.6 ± 0.2 | 0.17 ± 0.04 | 0.08 ± 0.02 | 0.2 ± 0.1 | 0.17 ± 0.01 | 0.4 ± 0.09 | 1.4 ± 0.3 | 0.7 ± 0.4 |
| CHR-1 | 0.3 ± 0.5 | <LOQ | <LOQ | <LOQ | 0.1 ± 0.1 | <LOQ | 0.08 ± 0.01 | 0.4 ± 0.2 | 0.14 ± 0.09 |
| CHR-6et | <LOQ | <LOQ | <LOQ | <LOQ | <LOQ | <LOQ | <LOQ | <LOQ | <LOQ |
| CHR-6pro | <LOQ | <LOQ | <LOQ | <LOQ | <LOQ | <LOQ | <LOQ | <LOQ | <LOQ |
| CHR-6but | <LOQ | <LOQ | <LOQ | <LOQ | 0.01 ± 0.02 | <LOQ | <LOQ | <LOQ | <LOQ |
| BBF | 1 ± 1 | 0.2 ± 0.1 | <LOQ | 0.05 ± 0.01 | 0.2 ± 0.4 | 0.07 ± 0.02 | 0.2 ± 0.01 | 0.7 ± 0.5 | 0.5 ± 0.4 |
| BKF | 0.2 ± 0.3 | <LOQ | <LOQ | <LOQ | 0.03 ± 0.05 | <LOQ | 0.02 ± 0.03 | 0.3 ± 0.1 | 0.1 ± 0.1 |
| BEP | 1 ± 1 | 0.3 ± 0.2 | 0.04 ± 0.06 | 0.03 ± 0.01 | 0.4 ± 0.6 | 0.06 ± 0.01 | 0.31 ± 0.01 | 1 ± 0.5 | 0.4 ± 0.2 |
| BAP | 0.2 ± 0.3 | <LOQ | <LOQ | <LOQ | 0.1 ± 0.2 | <LOQ | <LOQ | 0.3 ± 0.2 | <LOQ |
| PER | 0.1 ± 0.1 | <LOQ | <LOQ | <LOQ | <LOQ | <LOQ | <LOQ | <LOQ | <LOQ |
| IND | 0.02 ± 0.04 | <LOQ | <LOQ | <LOQ | 0.1 ± 0.1 | <LOQ | <LOQ | 0.04 ± 0.06 | <LOQ |
| DBA | 0.1 ± 0.1 | <LOQ | <LOQ | <LOQ | <LOQ | <LOQ | 0.01 ± 0.01 | 0.13 ± 0.06 | 0.03 ± 0.05 |
| BGP | 0.2 ± 0.3 | 0.02 ± 0.03 | <LOQ | <LOQ | 1 ± 2 | 0.003 ± 0.006 | 0.02 ± 0.03 | 0.3 ± 0.2 | 0.17 ± 0.06 |
| C1-BT | <LOQ | 0 ± 0.01 | <LOQ | 0.01 ± 0.01 | 0.03 ± 0.01 | 0.003 ± 0.006 | NA | NA | 0.06 ± 0.03 |
| C2-BT | <LOQ | <LOQ | 0.06 ± 0.06 | <LOQ | <LOQ | <LOQ | NA | NA | 0.08 ± 0.07 |
| C3-BT | 0.7 ± 0.2 | 0.1 ± 0.2 | 0.5 ± 0.1 | 0.6 ± 0.9 | 1.4 ± 0.5 | <LOQ | NA | NA | 1.4 ± 0.1 |
| C4-BT | 0.05 ± 0.04 | 0.05 ± 0.03 | 0.3 ± 0.03 | 0.4 ± 0.1 | 0.4 ± 0.3 | 0.54 ± 0.08 | NA | NA | 0.62 ± 0.02 |
| C1-NAP | 0.1 ± 0.1 | 0.31 ± 0.07 | 3.7 ± 0.2 | 3.3 ± 0.3 | 4.1 ± 0.2 | 3.5 ± 0.8 | NA | NA | 6 ± 1 |
| C2-NAP | 3.6 ± 0.5 | 2 ± 0.1 | 10.7 ± 0.3 | 11.2 ± 0.3 | 16 ± 1 | 11.5 ± 0.4 | NA | NA | 21 ± 1 |
| C3-NAP | 9 ± 0.8 | 5.5 ± 0.3 | 17.1 ± 0.4 | 15 ± 1 | 27 ± 4 | 18.3 ± 0.3 | NA | NA | 37 ± 2 |
| C4-NAP | 6 ± 2 | 2.4 ± 0.2 | 8 ± 0.2 | 7.3 ± 0.4 | 13 ± 2 | 9.8 ± 0.1 | NA | NA | 16.1 ± 0.9 |
| C1-FLU | 2 ± 1 | <LOQ | 2.3 ± 0.08 | 1.1 ± 0.2 | 3.4 ± 0.3 | 1.1 ± 0.2 | NA | NA | 6.2 ± 0.5 |
| C2-FLU | 4 ± 6 | 0.1 ± 0.2 | 1.36 ± 0.05 | 1.2 ± 0.3 | 2.7 ± 0.7 | 1.5 ± 0.2 | NA | NA | 4.9 ± 0.4 |
| C3-FLU | 6 ± 9 | 1 ± 0.7 | 0.87 ± 0.06 | 1.15 ± 0.07 | 2 ± 1 | 1.69 ± 0.07 | NA | NA | 4 ± 2 |
| C1-DBT | 2 ± 2 | 0.09 ± 0.09 | 1.4 ± 0.03 | 0.72 ± 0.06 | 2.5 ± 0.6 | 0.93 ± 0.02 | NA | NA | 4 ± 0.6 |
| C2-DBT | 12 ± 19 | 3 ± 2 | 2.5 ± 0.1 | 1.6 ± 0.1 | 5 ± 2 | 2.2 ± 0.1 | NA | NA | 7 ± 3 |
| C3-DBT | 14 ± 21 | 5 ± 3 | 1.36 ± 0.09 | 0.64 ± 0.04 | 3 ± 2 | 0.8 ± 0.3 | NA | NA | 6 ± 4 |
| C4-DBT | 19 ± 29 | 8 ± 5 | 1.4 ± 0.1 | 2.5 ± 0.6 | 5 ± 6 | 2.5 ± 0.6 | NA | NA | 10 ± 6 |
| C1-PHE | 8 ± 11 | 0.8 ± 0.7 | 3.8 ± 0.2 | 1.7 ± 0.2 | 5.7 ± 0.4 | 2.5 ± 0.2 | NA | NA | 10 ± 2 |
| C2-PHE | 25 ± 40 | 7 ± 4 | 3 ± 0.6 | 1.2 ± 0.2 | 5 ± 4 | 2.2 ± 0.6 | NA | NA | 11 ± 8 |
| C3-PHE | 26 ± 39 | 9 ± 5 | 2.2 ± 0.1 | 2.6 ± 0.4 | 4 ± 4 | 3.5 ± 0.1 | NA | NA | 13 ± 8 |
| C4-PHE | 29 ± 43 | 10 ± 6 | 1.5 ± 0.4 | 3 ± 1 | 7 ± 8 | 3.6 ± 0.4 | NA | NA | 16 ± 9 |
| C1-PYR | 8 ± 11 | 3 ± 1 | 0.7 ± 0.1 | 0.97 ± 0.02 | 3 ± 3 | 1.5 ± 0.1 | NA | NA | 4 ± 2 |
| C2-PYR | 13 ± 20 | 4 ± 2 | 0.67 ± 0.04 | 0.8 ± 0.1 | 4 ± 6 | 1.2 ± 0.3 | NA | NA | 6 ± 4 |
| C3-PYR | 16 ± 25 | 5 ± 3 | 0.93 ± 0.05 | 0.9 ± 0.2 | 6 ± 9 | 1.3 ± 0.2 | NA | NA | 10 ± 5 |
| C1-CHR | 6 ± 9 | 2 ± 1 | 0.28 ± 0.01 | 0.24 ± 0.06 | 0.7 ± 0.8 | 0.32 ± 0.08 | NA | NA | 2 ± 2 |
| C2-CHR | 5 ± 8 | 1 ± 1 | 0.2 ± 0.2 | 0.59 ± 0.04 | 3 ± 4 | 0.71 ± 0.08 | NA | NA | 3 ± 1 |
| C3-CHR | 6 ± 9 | 3 ± 1 | 0.85 ± 0.09 | 0.41 ± 0.07 | 9 ± 12 | 0.6 ± 0.09 | NA | NA | 4 ± 2 |
| C4-CHR | 12 ± 18 | 4 ± 3 | 1 ± 0.2 | 0.9 ± 0.8 | 19 ± 30 | 1.9 ± 0.3 | NA | NA | 8 ± 4 |
| ΣPAH | 39 ± 45 | 11 ± 4 | 36 ± 2 | 26 ± 2 | 52 ± 7 | 30 ± 2 | 65 ± 4 | 117 ± 30 | 91 ± 5 |
| tPAH | 239 ± 330 | 78 ± 37 | 73 ± 3 | 65 ± 7 | 161 ± 96 | 78 ± 4 |  |  | 238 ± 56 |

Table S7 continued.

| **Body burden (pg/embryo)** | **Haddock 0.21 µg/L Day 5** | **Haddock 0.21 µg/L Day 7** | **Haddock 0.21 µg/L Day 9** | **Haddock 0.76 µg/L Day 1** | **Haddock 0.76 µg/L Day 2** | **Haddock 0.76 µg/L Day 3** | **Haddock 0.76 µg/L Day 5** | **Haddock 0.76 µg/L Day 7** | **Haddock 0.76 µg/L Day 9** |
| --- | --- | --- | --- | --- | --- | --- | --- | --- | --- |
| BIP | 1.9 ± 0.2 | 1.8 ± 0.1 | 2.2 ± 0.2 | 4.5 ± 0.1 | 5.7 ± 0.3 | 5.9 ± 0.5 | 4.4 ± 0.3 | 5.2 ± 0.6 | 5 ± 0.3 |
| BT | <LOQ | <LOQ | 0.01 ± 0 | <LOQ | <LOQ | <LOQ | <LOQ | <LOQ | <LOQ |
| BT-2,5 | <LOQ | <LOQ | 0.07 ± 0.01 | 0.14 ± 0.01 | 0.19 ± 0.01 | 0.2 ± 0 | 0.16 ± 0.01 | 0.18 ± 0.02 | 0.14 ± 0.06 |
| BT-2,5,7 | 0.11 ± 0.01 | 0.12 ± 0 | 0.13 ± 0.01 | 0.18 ± 0.04 | 0.39 ± 0.06 | 0.51 ± 0.07 | 0.34 ± 0.01 | 0.37 ± 0.06 | 0.37 ± 0.03 |
| NAP | 3 ± 4 | 7 ± 3 | 8 ± 4 | 0.9 ± 0.4 | 0.8 ± 0.7 | 0.6 ± 0 | 0.2 ± 0.2 | 0.1 ± 0.1 | 0.03 ± 0.03 |
| NAP-2 | 4.3 ± 0.4 | 4 ± 0.3 | 5.1 ± 0.7 | 10.4 ± 0.1 | 10.6 ± 0.3 | 11 ± 1 | 8.2 ± 0.7 | 11 ± 1 | 10 ± 0.9 |
| NAP-1 | 1.7 ± 0.3 | 1.7 ± 0.1 | 2 ± 0.5 | 4.05 ± 0.06 | 4.1 ± 0.3 | 4.7 ± 0.6 | 3.3 ± 0.2 | 4.5 ± 0.4 | 3.8 ± 0.5 |
| NAP-2,6&2,7 | 6.7 ± 0.2 | 6.8 ± 0.4 | 5.6 ± 0.2 | 16.7 ± 0.2 | 25 ± 1 | 27 ± 0.8 | 18 ± 1 | 19 ± 1 | 17 ± 0.4 |
| NAP-1,4 | 5.1 ± 0.1 | 5.4 ± 0.1 | 5.2 ± 0.2 | 10.7 ± 0.2 | 17.7 ± 0.7 | 20.2 ± 0.6 | 14.5 ± 0.8 | 16 ± 1 | 16.4 ± 0.4 |
| NAP-1,3&2,3 | 3 ± 0.06 | 3.18 ± 0.02 | 3.18 ± 0.09 | 7.2 ± 0.3 | 11.7 ± 0.3 | 12.7 ± 0.5 | 9.3 ± 0.7 | 10.2 ± 0.8 | 10.6 ± 0.3 |
| NAP-1,3,7 | 2.6 ± 0.1 | 2.6 ± 0.1 | 1.1 ± 0.3 | 4 ± 0.1 | 11.15 ± 0.05 | 13.2 ± 0.3 | 5.9 ± 0.4 | 4.7 ± 0.4 | 5 ± 0.6 |
| NAP-2,3,5 | 11.2 ± 0.6 | 11.08 ± 0.08 | 6.1 ± 0.3 | 14.1 ± 0.4 | 38.5 ± 0.7 | 48.1 ± 0.3 | 33 ± 2 | 28 ± 2 | 21 ± 2 |
| NAP-1,2,3 | 0.51 ± 0.02 | 0.5 ± 0 | 0.3 ± 0.03 | 0.52 ± 0.03 | 1.58 ± 0.05 | 2 ± 0.1 | 1.48 ± 0.01 | 1.33 ± 0.06 | 1.1 ± 0.2 |
| NAP-1,2,5,6 | 0.8 ± 0.1 | 0.81 ± 0.02 | 0.49 ± 0.05 | 1.1 ± 0.1 | 5 ± 0.1 | 6.35 ± 0.04 | 3.8 ± 0.2 | 3.1 ± 0.3 | 3.1 ± 0.6 |
| NAP-1,4,6,7 | 0.7 ± 0.3 | 0.48 ± 0.01 | 0.27 ± 0.05 | 1.73 ± 0.09 | 6.4 ± 0.1 | 6.2 ± 0.3 | 2.56 ± 0.06 | 2.1 ± 0.3 | 3 ± 0.5 |
| ACY | <LOQ | <LOQ | 0.04 ± 0.05 | <LOQ | <LOQ | <LOQ | <LOQ | <LOQ | <LOQ |
| ACE | 0.17 ± 0.07 | 0.2 ± 0.04 | 0.16 ± 0.02 | 0.33 ± 0.02 | 0.6 ± 0.1 | 0.8 ± 0.2 | 0.49 ± 0.02 | 0.54 ± 0.09 | 0.57 ± 0.09 |
| DBF | 0.81 ± 0.09 | 0.76 ± 0.05 | 0.8 ± 0.2 | 1.74 ± 0.06 | 2.5 ± 0.3 | 2.7 ± 0.4 | 1.6 ± 0.1 | 2.1 ± 0.1 | 2.1 ± 0.2 |
| FLU | 1.6 ± 0.09 | 1.42 ± 0.04 | 1.6 ± 0.2 | 4.4 ± 0.1 | 7.6 ± 0.5 | 8.28 ± 0.01 | 5.4 ± 0.3 | 5.1 ± 0.5 | 5.1 ± 0.3 |
| FLU-9et | 0.15 ± 0.02 | 0.15 ± 0.01 | 0.14 ± 0.01 | 0.09 ± 0.01 | 0.43 ± 0.02 | 0.72 ± 0.05 | 0.73 ± 0.02 | 0.75 ± 0.05 | 0.61 ± 0.04 |
| FLU-1 | 2.8 ± 0.4 | 2.5 ± 0.1 | 1.26 ± 0.09 | 5.5 ± 0.2 | 15.3 ± 0.3 | 17.5 ± 0.2 | 7.9 ± 0.1 | 5.9 ± 0.5 | 6.2 ± 0.9 |
| FLU-9pro | 0.1 ± 0.1 | <LOQ | 0.01 ± 0.01 | <LOQ | 0.13 ± 0.04 | 0.32 ± 0 | 0.37 ± 0.05 | 0.44 ± 0.01 | 0.4 ± 0.01 |
| DBT | 1.7 ± 0.2 | 1.2 ± 0.02 | 0.92 ± 0.06 | 3.6 ± 0.1 | 8.2 ± 0.2 | 11 ± 1 | 6.1 ± 0.1 | 4.4 ± 0.2 | 4.1 ± 0.3 |
| DBT-4 | 1.7 ± 0.5 | 0.96 ± 0.02 | 0.47 ± 0.04 | 2.4 ± 0.1 | 8.6 ± 0.2 | 10.5 ± 0.1 | 4 ± 0.08 | 2.8 ± 0.3 | 3.2 ± 0.5 |
| DBT-4et | 0.8 ± 0.5 | 0.23 ± 0.01 | 0.2 ± 0.06 | 0.8 ± 0.06 | 3.77 ± 0.08 | 4.29 ± 0.08 | 1.38 ± 0.06 | 1.2 ± 0.2 | 2 ± 0.5 |
| DBT-4pro | 1.1 ± 0.6 | 0.23 ± 0.02 | 0.3 ± 0.1 | 0.58 ± 0.07 | 3.49 ± 0.06 | 4.6 ± 0.2 | 1.84 ± 0.05 | 1.5 ± 0.2 | 3.2 ± 0.7 |
| DBT-4but | 0.8 ± 0.6 | 0.15 ± 0.03 | 0.2 ± 0.1 | 0.32 ± 0.06 | 1.97 ± 0.03 | 2 ± 0.2 | 1.12 ± 0.02 | 1.3 ± 0.1 | 3.8 ± 0.9 |
| PHE | 4.8 ± 0.4 | 3.8 ± 0.4 | 1.6 ± 0.4 | 6.6 ± 0.4 | 16.6 ± 0.6 | 19.2 ± 0.2 | 8.8 ± 0.2 | 6.4 ± 0.5 | 6 ± 0.7 |
| ANT | <LOQ | <LOQ | <LOQ | <LOQ | <LOQ | <LOQ | <LOQ | <LOQ | <LOQ |
| PHE-3 | 1.6 ± 0.9 | 0.57 ± 0.03 | 0.5 ± 0.1 | 3.8 ± 0.2 | 11.8 ± 0.3 | 8.6 ± 0.5 | 2.6 ± 0.1 | 2.4 ± 0.2 | 4 ± 1 |
| PHE-2 | 1.6 ± 0.9 | 0.5 ± 0.05 | 0.3 ± 0.1 | 4 ± 0.2 | 11.9 ± 0.5 | 8.6 ± 0.7 | 2.7 ± 0.3 | 2.3 ± 0.3 | 3.6 ± 0.9 |
| PHE-9 | 4.89 ± 1.42 | 2.77 ± 0.05 | 1.7 ± 0.3 | 5.5 ± 0.2 | 19.5 ± 0.5 | 25.1 ± 0.7 | 10.3 ± 0.3 | 7.5 ± 0.9 | 8 ± 1 |
| PHE-1 | 2.04 ± 0.97 | 0.56 ± 0.06 | 0.5 ± 0.2 | 3.8 ± 0.2 | 11.5 ± 0.4 | 8 ± 1 | 2.4 ± 0.2 | 2.1 ± 0.5 | 3.7 ± 0.6 |
| PHE-3,6 | 2.2 ± 1.12 | 0.6 ± 0.02 | 0.7 ± 0.3 | 1.9 ± 0.2 | 7.7 ± 0.1 | 7 ± 0.4 | 2.9 ± 0.2 | 2.6 ± 0.4 | 5 ± 1 |
| PHE-1,7 | 2.32 ± 1.51 | 0.33 ± 0.02 | 0.8 ± 0.4 | 1.8 ± 0.2 | 4.29 ± 0.08 | 2.3 ± 0.1 | 1.4 ± 0.1 | 1.7 ± 0.4 | 7 ± 2 |
| PHE-1,2 | 0.4 ± 0.2 | 0.07 ± 0.01 | 0.12 ± 0.06 | 0.4 ± 0.04 | 0.89 ± 0.05 | 0.4 ± 0.03 | 0.23 ± 0.01 | 0.6 ± 0.3 | 0.8 ± 0.2 |
| PHE-2,6,9 | 4 ± 3 | 0.5 ± 0.2 | 0.9 ± 0.5 | 1.6 ± 0.1 | 5.8 ± 0.6 | 4.5 ± 0.6 | 2.6 ± 0.3 | 2.9 ± 0.4 | 9 ± 2 |
| PHE-1,2,6 | 0.7 ± 0.4 | 0.06 ± 0.04 | 0.12 ± 0.05 | 0.19 ± 0.04 | 0.59 ± 0.04 | 0.34 ± 0.01 | 0.19 ± 0.02 | 0.24 ± 0.06 | 1.22 ± 0.09 |
| PHE-1,2,7 | 0.2 ± 0.1 | <LOQ | 0.04 ± 0.02 | 0.09 ± 0.02 | 0.26 ± 0.04 | 0.19 ± 0.03 | 0.13 ± 0.01 | 0.12 ± 0.02 | 0.5 ± 0.02 |
| PHE-1,2,6,9 | 0.3 ± 0.3 | 0.01 ± 0.02 | 0.05 ± 0.03 | 0.1 ± 0.01 | 0.58 ± 0.04 | 0.5 ± 0.06 | 0.33 ± 0.03 | 0.36 ± 0.05 | 1 ± 0.2 |
| FLA | 0.5 ± 0.2 | 0.21 ± 0.01 | 0.2 ± 0.1 | 1.18 ± 0.05 | 2.56 ± 0.07 | 2.1 ± 0.2 | 0.71 ± 0.02 | 0.61 ± 0.06 | 1.1 ± 0.4 |
| PYR | 0.7 ± 0.3 | 0.13 ± 0.02 | 0.2 ± 0.1 | 0.78 ± 0.04 | 2.21 ± 0.03 | 1.3 ± 0.1 | 0.66 ± 0.01 | 0.64 ± 0.07 | 1.5 ± 0.3 |
| FLA-2 | 0.3 ± 0.2 | <LOQ | <LOQ | 0.25 ± 0.02 | 0.93 ± 0.09 | <LOQ | <LOQ | <LOQ | <LOQ |
| PYR-1 | 0.5 ± 0.3 | 0.09 ± 0.03 | 0.13 ± 0.06 | 0.29 ± 0.01 | 0.8 ± 0.1 | 0.71 ± 0.01 | 0.52 ± 0.06 | 0.6 ± 0.1 | 1.2 ± 0.09 |
| PYR-4,5 | 0.9 ± 0.6 | 0.12 ± 0.07 | <LOQ | <LOQ | <LOQ | <LOQ | <LOQ | <LOQ | <LOQ |
| PYR-1pro | <LOQ | <LOQ | <LOQ | <LOQ | <LOQ | <LOQ | <LOQ | <LOQ | <LOQ |
| PYR-1et | 0.03 ± 0.06 | <LOQ | 0.02 ± 0.01 | 0.01 ± 0.02 | 0.25 ± 0.01 | 0.24 ± 0.02 | 0.18 ± 0.05 | 0.16 ± 0.03 | 0.5 ± 0.3 |
| PYR-1but | 0.1 ± 0.1 | <LOQ | <LOQ | 0.03 ± 0.01 | 0.15 ± 0.03 | 0.17 ± 0.01 | 0.09 ± 0.08 | <LOQ | 0.2 ± 0.2 |
| BAA | 0.02 ± 0.04 | <LOQ | 0.07 ± 0.06 | 0.15 ± 0.02 | 0.34 ± 0.03 | 0.22 ± 0.01 | 0.18 ± 0.02 | 0.21 ± 0.04 | 0.42 ± 0.04 |
| CHR | 1.3 ± 0.7 | 0.25 ± 0.08 | 0.4 ± 0.2 | 0.85 ± 0.02 | 3.7 ± 0.4 | 4.6 ± 0.8 | 1.5 ± 0.2 | 1.5 ± 0.2 | 2.3 ± 0.9 |
| CHR-1 | 0.3 ± 0.2 | 0.06 ± 0.01 | 0.12 ± 0.05 | 0.09 ± 0.02 | 0.53 ± 0.08 | 0.37 ± 0.01 | 0.29 ± 0.04 | 0.26 ± 0.04 | 0.8 ± 0.2 |
| CHR-6et | <LOQ | <LOQ | <LOQ | <LOQ | <LOQ | <LOQ | <LOQ | <LOQ | <LOQ |
| CHR-6pro | <LOQ | <LOQ | <LOQ | <LOQ | <LOQ | <LOQ | <LOQ | <LOQ | <LOQ |
| CHR-6but | <LOQ | <LOQ | <LOQ | <LOQ | <LOQ | <LOQ | <LOQ | <LOQ | <LOQ |
| BBF | 0.7 ± 0.4 | 0.14 ± 0.03 | 0.18 ± 0.08 | 0.2 ± 0.05 | 0.87 ± 0.07 | 0.73 ± 0.03 | 0.69 ± 0.08 | 0.58 ± 0.02 | 1.8 ± 0.5 |
| BKF | 0.3 ± 0.2 | <LOQ | 0.04 ± 0.06 | 0.11 ± 0.01 | 0.47 ± 0.06 | 0.39 ± 0.06 | 0.31 ± 0.03 | 0.33 ± 0.04 | 0.8 ± 0.2 |
| BEP | 0.7 ± 0.4 | 0.16 ± 0.03 | 0.2 ± 0.1 | 0.28 ± 0.03 | 1.75 ± 0.06 | 1.42 ± 0.09 | 0.8 ± 0.09 | 0.85 ± 0.08 | 2 ± 0.3 |
| BAP | 0.2 ± 0.2 | <LOQ | <LOQ | <LOQ | <LOQ | <LOQ | <LOQ | <LOQ | <LOQ |
| PER | <LOQ | <LOQ | <LOQ | <LOQ | 0.17 ± 0.03 | 0.1 ± 0.04 | 0.05 ± 0.03 | 0.05 ± 0.05 | 0.3 ± 0.4 |
| IND | 0.02 ± 0.03 | <LOQ | <LOQ | <LOQ | <LOQ | <LOQ | <LOQ | <LOQ | 0.1 ± 0.1 |
| DBA | 0.11 ± 0.06 | 0.02 ± 0.03 | <LOQ | 0.15 ± 0.02 | 0.29 ± 0.02 | 0.2 ± 0.3 | 0.37 ± 0.03 | <LOQ | 0.6 ± 0.1 |
| BGP | 0.3 ± 0.1 | 0.1 ± 0.03 | 0.11 ± 0.01 | <LOQ | 0.7 ± 0.2 | 0.8 ± 0.2 | 0.57 ± 0.05 | 0.72 ± 0.04 | 1.2 ± 0.3 |
| C1-BT | NA | NA | 0.07 ± 0.01 | NA | NA | 0.06 ± 0 | NA | NA | 0.07 ± 0.01 |
| C2-BT | NA | NA | <LOQ | NA | NA | <LOQ | NA | NA | 0.05 ± 0.06 |
| C3-BT | NA | NA | 0.5 ± 0.3 | NA | NA | 4.75 ± 0.02 | NA | NA | 2.9 ± 0.3 |
| C4-BT | NA | NA | 0.56 ± 0.06 | NA | NA | 4.8 ± 0.2 | NA | NA | 2.7 ± 0.3 |
| C1-NAP | NA | NA | 7 ± 1 | NA | NA | 16 ± 2 | NA | NA | 14 ± 1 |
| C2-NAP | NA | NA | 18.1 ± 0.6 | NA | NA | 75 ± 2 | NA | NA | 56 ± 3 |
| C3-NAP | NA | NA | 21 ± 1 | NA | NA | 181 ± 5 | NA | NA | 83 ± 8 |
| C4-NAP | NA | NA | 10 ± 0.9 | NA | NA | 121 ± 2 | NA | NA | 62 ± 9 |
| C1-FLU | NA | NA | 1.2 ± 0.3 | NA | NA | 31.9 ± 0.2 | NA | NA | 10 ± 2 |
| C2-FLU | NA | NA | 2.8 ± 0.4 | NA | NA | 30 ± 4 | NA | NA | 20 ± 4 |
| C3-FLU | NA | NA | 2.7 ± 0.5 | NA | NA | 21 ± 1 | NA | NA | 22 ± 4 |
| C1-DBT | NA | NA | 1.02 ± 0.09 | NA | NA | 20.74 ± 0.02 | NA | NA | 6 ± 1 |
| C2-DBT | NA | NA | 3 ± 1 | NA | NA | 47 ± 1 | NA | NA | 29 ± 6 |
| C3-DBT | NA | NA | 3 ± 3 | NA | NA | 35.5 ± 0.5 | NA | NA | 48 ± 12 |
| C4-DBT | NA | NA | 7 ± 4 | NA | NA | 47 ± 4 | NA | NA | 95 ± 23 |
| C1-PHE | NA | NA | 3 ± 0.6 | NA | NA | 51 ± 3 | NA | NA | 19 ± 4 |
| C2-PHE | NA | NA | 7 ± 4 | NA | NA | 53 ± 4 | NA | NA | 57 ± 14 |
| C3-PHE | NA | NA | 9 ± 4 | NA | NA | 46 ± 4 | NA | NA | 75 ± 19 |
| C4-PHE | NA | NA | 12 ± 5 | NA | NA | 63 ± 3 | NA | NA | 126 ± 26 |
| C1-PYR | NA | NA | 3.4 ± 0.8 | NA | NA | 15.1 ± 0.3 | NA | NA | 23 ± 3 |
| C2-PYR | NA | NA | 4 ± 2 | NA | NA | 19 ± 2 | NA | NA | 45 ± 10 |
| C3-PYR | NA | NA | 6 ± 3 | NA | NA | 33 ± 2 | NA | NA | 85 ± 24 |
| C1-CHR | NA | NA | 2 ± 1 | NA | NA | 7.7 ± 0.6 | NA | NA | 22 ± 6 |
| C2-CHR | NA | NA | 2 ± 0.7 | NA | NA | 10.4 ± 0.6 | NA | NA | 26 ± 7 |
| C3-CHR | NA | NA | 3 ± 1 | NA | NA | 20.6 ± 0.9 | NA | NA | 60 ± 11 |
| C4-CHR | NA | NA | 8 ± 2 | NA | NA | 35 ± 4 | NA | NA | 105 ± 16 |
| ΣPAH | 85 ± 14 | 64 ± 4 | 55 ± 9 | 130 ± 4 | 299 ± 6 | 321 ± 12 | 177 ± 8 | 167 ± 12 | 192 ± 20 |
| tPAH |  |  | 153 ± 41 |  |  | 1050 ± 46 |  |  | 1128 ± 209 |

Table S7 continued.

| **Body burden (pg/embryo)** | **Haddock 0.76 µg/L Day 10** | **Haddock 0.76 µg/L Day 11** | **Haddock 0.76 µg/L Day 12** | **Haddock 2.7 µg/L Day 1** | **Haddock 2.7 µg/L Day 2** | **Haddock 2.7 µg/L Day 3** | **Haddock 2.7 µg/L Day 5** | **Haddock 2.7 µg/L Day 7** | **Haddock 2.7 µg/L Day 9** |
| --- | --- | --- | --- | --- | --- | --- | --- | --- | --- |
| BIP | 2.6 ± 0.6 | 0.08 ± 0.08 | 0.03 ± 0.05 | 19.7 ± 0.6 | 16.8 ± 0.7 | 18.1 ± 0.8 | 16.6 ± 0.4 | 20.1 ± 0.5 | 15 ± 1 |
| BT | <LOQ | <LOQ | <LOQ | <LOQ | <LOQ | <LOQ | <LOQ | <LOQ | 0 ± 0.01 |
| BT-2,5 | 0.1 ± 0.02 | <LOQ | <LOQ | 0.62 ± 0.02 | 0.56 ± 0.02 | 0.6 ± 0.03 | 0.57 ± 0.01 | 0.71 ± 0.01 | 0.52 ± 0.03 |
| BT-2,5,7 | 0.22 ± 0.06 | <LOQ | <LOQ | 0.79 ± 0.04 | 1.1 ± 0.3 | 1.7 ± 0.1 | 1.61 ± 0.07 | 1.71 ± 0.04 | 1.3 ± 0.2 |
| NAP | 0 ± 0.01 | <LOQ | <LOQ | 4.4 ± 0.3 | 1.8 ± 0.4 | 1.8 ± 0.4 | 1.7 ± 0.6 | 3.4 ± 0.4 | 0.8 ± 0.2 |
| NAP-2 | 6 ± 1 | 0.1 ± 0.2 | 0.1 ± 0.1 | 46 ± 1 | 31 ± 1 | 32 ± 2 | 31 ± 1 | 42.9 ± 0.9 | 28 ± 1 |
| NAP-1 | 2.2 ± 0.4 | <LOQ | <LOQ | 19.6 ± 0.4 | 13 ± 0.8 | 14.1 ± 0.9 | 13.5 ± 0.4 | 18.7 ± 0.4 | 11.2 ± 0.7 |
| NAP-2,6&2,7 | 8 ± 2 | 0.5 ± 0.1 | 0.3 ± 0.1 | 69 ± 3 | 71 ± 2 | 81 ± 1 | 71 ± 2 | 79.9 ± 0.6 | 57 ± 3 |
| NAP-1,4 | 7 ± 1 | 0.5 ± 0.08 | 0.2 ± 0.1 | 52 ± 2 | 57 ± 2 | 68 ± 2 | 61 ± 1 | 69.4 ± 0.9 | 51 ± 3 |
| NAP-1,3&2,3 | 4.7 ± 0.9 | 0.55 ± 0.05 | 0.36 ± 0.08 | 37 ± 1 | 41 ± 2 | 47 ± 1 | 43 ± 1 | 49.3 ± 0.5 | 35 ± 3 |
| NAP-1,3,7 | 1.9 ± 0.3 | 0.16 ± 0.07 | 0.15 ± 0.06 | 29 ± 2 | 45 ± 3 | 55 ± 3 | 49 ± 2 | 40 ± 2 | 19.1 ± 0.4 |
| NAP-2,3,5 | 9 ± 2 | 1.8 ± 0.3 | 1 ± 0.2 | 79 ± 4 | 124 ± 6 | 158 ± 8 | 155 ± 3 | 141 ± 7 | 88 ± 9 |
| NAP-1,2,3 | 0.5 ± 0.1 | 0.04 ± 0.01 | 0.01 ± 0.01 | 3.1 ± 0.2 | 5.4 ± 0.3 | 7 ± 0.4 | 7.1 ± 0.2 | 6.4 ± 0.1 | 4.4 ± 0.4 |
| NAP-1,2,5,6 | 0.8 ± 0.2 | 0.2 ± 0.1 | 0.1 ± 0.03 | 7.5 ± 0.6 | 15.1 ± 0.9 | 20 ± 1 | 21 ± 0.5 | 17.5 ± 0.5 | 15 ± 2 |
| NAP-1,4,6,7 | 0.8 ± 0.2 | 0.08 ± 0.05 | 0.06 ± 0.02 | 12.7 ± 0.9 | 22 ± 1 | 24.7 ± 0.2 | 17.3 ± 0.6 | 14.1 ± 0.7 | 12 ± 2 |
| ACY | <LOQ | <LOQ | <LOQ | <LOQ | 0.02 ± 0.01 | 0.05 ± 0.01 | 0.05 ± 0.02 | 0.02 ± 0.01 | 0.01 ± 0.01 |
| ACE | 0.27 ± 0.07 | 0.05 ± 0.02 | 0 ± 0.01 | 2.7 ± 0.8 | 2.4 ± 0.4 | 2.5 ± 0.07 | 2.5 ± 0.1 | 2.62 ± 0.04 | 2.1 ± 0.4 |
| DBF | 0.8 ± 0.2 | <LOQ | <LOQ | 9.7 ± 0.3 | 9.9 ± 0.2 | 11 ± 1 | 9.7 ± 0.3 | 10.3 ± 0.2 | 7.2 ± 0.8 |
| FLU | 2.3 ± 0.3 | 0.04 ± 0.05 | 0.01 ± 0.02 | 24 ± 1 | 25 ± 1 | 29 ± 1 | 24 ± 1 | 25 ± 2 | 18 ± 3 |
| FLU-9et | 0.26 ± 0.06 | 0.11 ± 0.03 | 0.04 ± 0.01 | 0.67 ± 0.05 | 1.44 ± 0.08 | 2.2 ± 0.1 | 2.9 ± 0.08 | 3.12 ± 0.06 | 2.7 ± 0.1 |
| FLU-1 | 2.6 ± 0.3 | 0.24 ± 0.04 | 0.18 ± 0.04 | 33 ± 2 | 47 ± 2 | 55 ± 2 | 42 ± 1 | 25 ± 17 | 22 ± 3 |
| FLU-9pro | 0.13 ± 0.03 | 0.05 ± 0.02 | 0.02 ± 0.01 | 0.19 ± 0.02 | 0.53 ± 0.04 | 1 ± 0.1 | 1.36 ± 0.09 | 1.69 ± 0.05 | 1.68 ± 0.06 |
| DBT | 1.8 ± 0.3 | 0.11 ± 0.03 | 0.02 ± 0.02 | 19 ± 1 | 26.8 ± 0.7 | 35 ± 3 | 26.7 ± 0.7 | 23 ± 1 | 14 ± 1 |
| DBT-4 | 1 ± 0.3 | 0.13 ± 0.08 | 0.11 ± 0.04 | 16 ± 1 | 27.5 ± 0.8 | 34.8 ± 0.9 | 25.6 ± 0.7 | 18.8 ± 0.3 | 13 ± 2 |
| DBT-4et | 0.4 ± 0.1 | 0.1 ± 0.07 | 0.12 ± 0.05 | 5.5 ± 0.5 | 11.2 ± 0.4 | 14.1 ± 0.2 | 9.7 ± 0.4 | 7.3 ± 0.5 | 9 ± 2 |
| DBT-4pro | 0.5 ± 0.2 | 0.08 ± 0.09 | 0.16 ± 0.1 | 4 ± 0.3 | 8.6 ± 0.3 | 11.44 ± 0.05 | 9.8 ± 0.2 | 8.2 ± 0.7 | 13 ± 2 |
| DBT-4but | 0.4 ± 0.2 | 0.04 ± 0.04 | 0.07 ± 0.05 | 2.1 ± 0.2 | 3.83 ± 0.09 | 4.8 ± 0.2 | 4.6 ± 0.1 | 4.6 ± 0.9 | 13 ± 2 |
| PHE | 2.5 ± 0.5 | 0.2 ± 0.2 | 0.03 ± 0.04 | 39 ± 2 | 54 ± 3 | 64 ± 2 | 51 ± 2 | 39.45 ± 0.03 | 21 ± 2 |
| ANT | <LOQ | <LOQ | <LOQ | <LOQ | <LOQ | <LOQ | <LOQ | <LOQ | <LOQ |
| PHE-3 | 0.9 ± 0.4 | 0.08 ± 0.09 | 0.05 ± 0.02 | 24 ± 2 | 34 ± 1 | 36 ± 1 | 20.6 ± 0.9 | 16 ± 1 | 15 ± 2 |
| PHE-2 | 1 ± 0.3 | 0.11 ± 0.09 | 0.07 ± 0.02 | 24 ± 2 | 35 ± 2 | 35.9 ± 0.9 | 18 ± 1 | 15.3 ± 0.9 | 15 ± 2 |
| PHE-9 | 2.7 ± 0.7 | 0.4 ± 0.3 | 0.4 ± 0.1 | 33 ± 2 | 54 ± 2 | 68 ± 2 | 51 ± 1 | 39.6 ± 0.9 | 29 ± 4 |
| PHE-1 | 0.9 ± 0.3 | 0.1 ± 0.09 | 0.06 ± 0.03 | 24 ± 2 | 33 ± 1 | 33.8 ± 0.6 | 17.4 ± 0.6 | 14 ± 1 | 14 ± 3 |
| PHE-3,6 | 0.9 ± 0.4 | 0.2 ± 0.1 | 0.2 ± 0.1 | 13 ± 1 | 21 ± 1 | 23 ± 0.7 | 16.7 ± 0.6 | 15 ± 1 | 19 ± 3 |
| PHE-1,7 | 0.7 ± 0.3 | 0.06 ± 0.05 | 0.01 ± 0.02 | 12.6 ± 0.9 | 11 ± 1 | 11 ± 1 | 9 ± 1 | 11 ± 3 | 26 ± 4 |
| PHE-1,2 | 0.11 ± 0.05 | <LOQ | <LOQ | 2.4 ± 0.3 | 2.3 ± 0.2 | 2.1 ± 0.2 | 4 ± 2 | 1.7 ± 0.3 | 3.8 ± 0.4 |
| PHE-2,6,9 | 1.3 ± 0.7 | 0.14 ± 0.06 | 0.11 ± 0.04 | 12.3 ± 0.8 | 13.1 ± 0.7 | 14 ± 1 | 13 ± 2 | 12 ± 1 | 29 ± 7 |
| PHE-1,2,6 | <LOQ | <LOQ | <LOQ | 1.9 ± 0.1 | 1.3 ± 0.2 | 1.3 ± 0.3 | 1.6 ± 0.4 | 1.4 ± 0.3 | 4 ± 0.9 |
| PHE-1,2,7 | 0.06 ± 0.02 | 0.02 ± 0.02 | 0.02 ± 0.02 | 0.66 ± 0.05 | 0.66 ± 0.07 | 0.66 ± 0.06 | 0.7 ± 0.1 | 0.66 ± 0.04 | 1.5 ± 0.4 |
| PHE-1,2,6,9 | 0.09 ± 0.07 | <LOQ | <LOQ | 0.94 ± 0.03 | 1.2 ± 0.1 | 1.3 ± 0.2 | 1.5 ± 0.1 | 1.39 ± 0.06 | 3.4 ± 0.7 |
| FLA | 0.2 ± 0.1 | <LOQ | <LOQ | 4.3 ± 0.4 | 6.5 ± 0.4 | 6.5 ± 0.2 | 4.8 ± 0.1 | 4.1 ± 0.5 | 5.2 ± 0.8 |
| PYR | 0.2 ± 0.1 | 0.01 ± 0.02 | <LOQ | 4.3 ± 0.4 | 6.2 ± 0.4 | 5.7 ± 0.4 | 4 ± 0.1 | 3.8 ± 0.5 | 6.2 ± 0.9 |
| FLA-2 | <LOQ | <LOQ | 0.05 ± 0.08 | 1.7 ± 0.1 | 2.22 ± 0.05 | 2.1 ± 0.1 | 1.6 ± 0.3 | 1 ± 0.2 | <LOQ |
| PYR-1 | 0.29 ± 0.09 | 0.11 ± 0.03 | 0.1 ± 0.02 | 2 ± 0.1 | 2 ± 0.3 | 1.8 ± 0.2 | 1.7 ± 0.2 | 1.7 ± 0.3 | 3.6 ± 0.7 |
| PYR-4,5 | <LOQ | <LOQ | <LOQ | <LOQ | <LOQ | <LOQ | <LOQ | <LOQ | <LOQ |
| PYR-1pro | <LOQ | <LOQ | <LOQ | <LOQ | <LOQ | <LOQ | <LOQ | <LOQ | <LOQ |
| PYR-1et | <LOQ | <LOQ | <LOQ | <LOQ | <LOQ | <LOQ | <LOQ | <LOQ | 1.6 ± 0.7 |
| PYR-1but | <LOQ | <LOQ | <LOQ | <LOQ | <LOQ | <LOQ | <LOQ | <LOQ | 1.1 ± 0.2 |
| BAA | 0.1 ± 0.02 | <LOQ | <LOQ | 0.74 ± 0.06 | 0.72 ± 0.05 | 0.7 ± 0.1 | 0.72 ± 0.07 | 0.7 ± 0.04 | 1.5 ± 0.3 |
| CHR | 0.5 ± 0.2 | 0.07 ± 0.08 | 0.19 ± 0.13 | 4.9 ± 0.5 | 8.3 ± 0.3 | 9.3 ± 0.8 | 7 ± 0.8 | 5.9 ± 0.6 | 9 ± 2 |
| CHR-1 | <LOQ | <LOQ | <LOQ | 0.7 ± 0.06 | 0.98 ± 0.05 | 1.1 ± 0.2 | 1.1 ± 0.1 | 1.2 ± 0.2 | 3.9 ± 0.9 |
| CHR-6et | <LOQ | <LOQ | <LOQ | <LOQ | <LOQ | <LOQ | <LOQ | <LOQ | <LOQ |
| CHR-6pro | <LOQ | <LOQ | <LOQ | <LOQ | <LOQ | <LOQ | <LOQ | <LOQ | <LOQ |
| CHR-6but | <LOQ | <LOQ | <LOQ | <LOQ | <LOQ | <LOQ | <LOQ | <LOQ | <LOQ |
| BBF | 0.2 ± 0.1 | <LOQ | <LOQ | 1 ± 0.1 | 1.4 ± 0.1 | 1.6 ± 0.2 | 2 ± 0.2 | 1.9 ± 0.4 | 6.4 ± 0.6 |
| BKF | 0.07 ± 0.04 | <LOQ | <LOQ | 0.6 ± 0.09 | 0.9 ± 0.1 | 0.94 ± 0.09 | 1.1 ± 0.1 | 1.2 ± 0.3 | 2.9 ± 0.6 |
| BEP | 0.2 ± 0.1 | 0 ± 0.01 | 0.02 ± 0.03 | 1.6 ± 0.2 | 3.2 ± 0.3 | 3.4 ± 0.3 | 3 ± 0.5 | 3 ± 0.5 | 7 ± 1 |
| BAP | <LOQ | 0.1 ± 0.2 | <LOQ | 0.26 ± 0.09 | 0.7 ± 0.2 | 0.9 ± 0.2 | 0.6 ± 0.2 | 0.8 ± 0.3 | 1.9 ± 0.2 |
| PER | <LOQ | <LOQ | <LOQ | 0.22 ± 0 | 0.27 ± 0.06 | 0.39 ± 0.02 | 0.4 ± 0.01 | 0.5 ± 0.2 | 1.9 ± 0.5 |
| IND | <LOQ | <LOQ | <LOQ | <LOQ | <LOQ | 0.01 ± 0.01 | 0.1 ± 0.2 | 0.1 ± 0.2 | 0.6 ± 0.2 |
| DBA | <LOQ | <LOQ | <LOQ | 0.34 ± 0.03 | 0.45 ± 0.03 | 0.67 ± 0.05 | 0.74 ± 0.05 | 0.8 ± 0.2 | 1.3 ± 0.3 |
| BGP | <LOQ | <LOQ | <LOQ | 0.29 ± 0.09 | 1.1 ± 0.1 | 1.3 ± 0.1 | 1.9 ± 0.9 | 2 ± 1 | 3.1 ± 0.6 |
| C1-BT | 0.08 ± 0.01 | NA | NA | NA | NA | 0.16 ± 0.01 | NA | NA | 0.2 ± 0.02 |
| C2-BT | <LOQ | NA | NA | NA | NA | 2.2 ± 0.1 | NA | NA | 1.1 ± 0.1 |
| C3-BT | 1.8 ± 0.6 | NA | NA | NA | NA | 14 ± 0.9 | NA | NA | 9.8 ± 0.4 |
| C4-BT | 1.6 ± 0.3 | NA | NA | NA | NA | 15.9 ± 0.6 | NA | NA | 13 ± 1 |
| C1-NAP | 8 ± 2 | NA | NA | NA | NA | 46 ± 3 | NA | NA | 39 ± 2 |
| C2-NAP | 27 ± 5 | NA | NA | NA | NA | 248 ± 6 | NA | NA | 196 ± 12 |
| C3-NAP | 37 ± 8 | NA | NA | NA | NA | 580 ± 15 | NA | NA | 375 ± 36 |
| C4-NAP | 23 ± 6 | NA | NA | NA | NA | 397 ± 12 | NA | NA | 311 ± 29 |
| C1-FLU | 4.3 ± 0.7 | NA | NA | NA | NA | 111 ± 4 | NA | NA | 41 ± 5 |
| C2-FLU | 7 ± 2 | NA | NA | NA | NA | 126 ± 8 | NA | NA | 77 ± 4 |
| C3-FLU | 9 ± 2 | NA | NA | NA | NA | 73 ± 4 | NA | NA | 94 ± 11 |
| C1-DBT | 2.1 ± 0.6 | NA | NA | NA | NA | 69 ± 3 | NA | NA | 27 ± 4 |
| C2-DBT | 6 ± 2 | NA | NA | NA | NA | 162 ± 9 | NA | NA | 126 ± 24 |
| C3-DBT | 6 ± 3 | NA | NA | NA | NA | 106 ± 3 | NA | NA | 192 ± 30 |
| C4-DBT | 9 ± 5 | NA | NA | NA | NA | 112 ± 4 | NA | NA | 323 ± 50 |
| C1-PHE | 6 ± 2 | NA | NA | NA | NA | 174 ± 4 | NA | NA | 74 ± 11 |
| C2-PHE | 9 ± 3 | NA | NA | NA | NA | 214 ± 8 | NA | NA | 153 ± 101 |
| C3-PHE | 12 ± 5 | NA | NA | NA | NA | 144 ± 13 | NA | NA | 314 ± 89 |
| C4-PHE | 23 ± 10 | NA | NA | NA | NA | 161 ± 9 | NA | NA | 453 ± 80 |
| C1-PYR | 5 ± 1 | NA | NA | NA | NA | 43 ± 4 | NA | NA | 83 ± 13 |
| C2-PYR | 6 ± 2 | NA | NA | NA | NA | 61 ± 5 | NA | NA | 166 ± 27 |
| C3-PYR | 7 ± 3 | NA | NA | NA | NA | 85 ± 6 | NA | NA | 305 ± 52 |
| C1-CHR | 2 ± 1 | NA | NA | NA | NA | 23 ± 3 | NA | NA | 81 ± 14 |
| C2-CHR | 0.01 ± 0.01 | NA | NA | NA | NA | 23 ± 2 | NA | NA | 88 ± 17 |
| C3-CHR | 0.2 ± 0.3 | NA | NA | NA | NA | 38 ± 4 | NA | NA | 174 ± 26 |
| C4-CHR | 3 ± 2 | NA | NA | NA | NA | 58 ± 6 | NA | NA | 266 ± 42 |
| ΣPAH | 68 ± 15 | 7 ± 2 | 4 ± 2 | 708 ± 36 | 903 ± 38 | 1059 ± 12 | 884 ± 7 | 831 ± 33 | 694 ± 70 |
| tPAH | 226 ± 66 |  |  |  |  | 3280 ± 108 |  |  | 4106 ± 482 |

Table S7 continued.

| **Body burden (pg/embryo)** | **Haddock 2.7 µg/L**  **Day 10** | **Haddock 2.7 µg/L**  **Day 11** | **Haddock 2.7 µg/L**  **Day 12** | **Haddock 3.5 µg/L Day 3** | **Haddock 3.5 µg/L Day 9** | **Haddock 8.6 µg/L 12 hrs** | **Haddock 8.6 µg/L Day 1** | **Haddock 8.6 µg/L Day 2** | **Haddock 8.6 µg/L Day 3** |
| --- | --- | --- | --- | --- | --- | --- | --- | --- | --- |
| BIP | 8.4 ± 0.4 | 0.08 ± 0.01 | <LOQ | 17.9 ± 0.6 | 16.3 ± 0.5 | 31 ± 4 | 34 ± 5 | 49 ± 4 | 52 ± 1 |
| BT | <LOQ | <LOQ | <LOQ | <LOQ | 0 ± 0.01 | <LOQ | <LOQ | <LOQ | <LOQ |
| BT-2,5 | 0.29 ± 0.02 | 0 ± 0.01 | <LOQ | 0.68 ± 0.04 | 0.55 ± 0.02 | 0.8 ± 0.1 | 1 ± 0.2 | 1.7 ± 0.2 | 1.74 ± 0.03 |
| BT-2,5,7 | 0.6 ± 0.05 | <LOQ | <LOQ | 2.1 ± 0.1 | 1.36 ± 0.06 | 1 ± 0.3 | 1.2 ± 0.4 | 3.2 ± 0.1 | 3.8 ± 0.3 |
| NAP | 0.2 ± 0.3 | <LOQ | <LOQ | 4 ± 0.2 | 1.4 ± 0.4 | 8 ± 2 | 9 ± 1 | 12 ± 3 | 12.3 ± 0.6 |
| NAP-2 | 16.9 ± 0.4 | <LOQ | <LOQ | 32 ± 2 | 30 ± 1 | 66 ± 10 | 72 ± 4 | 104 ± 13 | 110 ± 2 |
| NAP-1 | 7.1 ± 0.2 | <LOQ | <LOQ | 13.5 ± 0.4 | 11.9 ± 0.4 | 27 ± 4 | 31 ± 2 | 45 ± 6 | 48.5 ± 0.9 |
| NAP-2,6&2,7 | 33 ± 1 | 0.9 ± 0.1 | 0.2 ± 0.1 | 97 ± 1 | 60 ± 2 | 96 ± 15 | 112 ± 25 | 188 ± 12 | 184 ± 4 |
| NAP-1,4 | 30 ± 1 | 1.1 ± 0.2 | 0.34 ± 0.06 | 77 ± 2 | 52.8 ± 0.8 | 64 ± 10 | 85 ± 14 | 146 ± 9 | 149 ± 3 |
| NAP-1,3&2,3 | 21.4 ± 0.9 | 1.2 ± 0.2 | 0.55 ± 0.06 | 47 ± 2 | 36 ± 2 | 45 ± 7 | 54 ± 12 | 98 ± 7 | 100 ± 3 |
| NAP-1,3,7 | 14 ± 2 | 0.5 ± 0.1 | 0.16 ± 0.08 | 93 ± 3 | 21 ± 1 | 25 ± 5 | 31 ± 10 | 75.9 ± 0.9 | 82 ± 1 |
| NAP-2,3,5 | 52 ± 3 | 5.9 ± 0.8 | 2.7 ± 0.2 | 296 ± 11 | 96 ± 6 | 84 ± 15 | 102 ± 29 | 244 ± 2 | 271 ± 4 |
| NAP-1,2,3 | 2.2 ± 0.1 | 0.12 ± 0.03 | <LOQ | 13 ± 0.5 | 5.2 ± 0.3 | 3.5 ± 0.7 | 5 ± 2 | 12.1 ± 0.5 | 14.9 ± 0.2 |
| NAP-1,2,5,6 | 6 ± 0.8 | 0.5 ± 0.08 | 0.24 ± 0.09 | 42 ± 2 | 18 ± 2 | 9 ± 2 | 11 ± 4 | 31.7 ± 0.9 | 38.1 ± 0.3 |
| NAP-1,4,6,7 | 5 ± 1 | 0.26 ± 0.05 | 0.06 ± 0.08 | 44 ± 1 | 16 ± 2 | 16 ± 3 | 19 ± 7 | 47 ± 0.8 | 49.4 ± 0.7 |
| ACY | 0.02 ± 0.01 | <LOQ | <LOQ | 0.02 ± 0.04 | 0.02 ± 0.02 | 0.08 ± 0.01 | 0.08 ± 0.02 | 0.04 ± 0.03 | 0.06 ± 0.02 |
| ACE | 1.22 ± 0.01 | 0.16 ± 0.01 | 0.15 ± 0.01 | 3.1 ± 0.7 | 3 ± 0.3 | 2.1 ± 0.3 | 2.5 ± 0.9 | 5.1 ± 0.1 | 7 ± 1 |
| DBF | 4.7 ± 0.2 | <LOQ | <LOQ | 11.3 ± 0.4 | 7.7 ± 0.4 | 14 ± 2 | 17 ± 3 | 26 ± 1 | 25.9 ± 0.7 |
| FLU | 12 ± 1 | 0.18 ± 0.09 | 0.23 ± 0.03 | 28 ± 2 | 19.1 ± 0.9 | 35 ± 5 | 41 ± 9 | 65 ± 2 | 64 ± 2 |
| FLU-9et | 1.41 ± 0.04 | 0.4 ± 0.07 | 0.38 ± 0.05 | 4.3 ± 0.1 | 3.1 ± 0.1 | 0.8 ± 0.2 | 1 ± 0.4 | 3.18 ± 0.05 | 4.22 ± 0.03 |
| FLU-1 | 15 ± 2 | <LOQ | <LOQ | 95 ± 2 | 28 ± 2 | 36 ± 6 | 47 ± 13 | 100 ± 1 | 103 ± 2 |
| FLU-9pro | 0.78 ± 0.02 | 0.29 ± 0.04 | 0.38 ± 0.08 | 1.71 ± 0.05 | 2.2 ± 0.2 | 0.23 ± 0.07 | 0.3 ± 0.1 | 1.01 ± 0.09 | 1.64 ± 0.04 |
| DBT | 9.5 ± 0.8 | 0.35 ± 0.01 | 0.01 ± 0.01 | 36.9 ± 0.7 | 16.3 ± 0.9 | 28 ± 5 | 34 ± 9 | 68 ± 1 | 67 ± 2 |
| DBT-4 | 7 ± 1 | 0.3 ± 0.02 | 0.05 ± 0.07 | 61.7 ± 0.6 | 17 ± 2 | 20 ± 4 | 23 ± 8 | 59 ± 2 | 63 ± 1 |
| DBT-4et | 2.8 ± 0.7 | 0.26 ± 0.05 | 0.2 ± 0.1 | 24.8 ± 0.3 | 12 ± 2 | 8 ± 2 | 10 ± 4 | 26 ± 1 | 29 ± 0.4 |
| DBT-4pro | 2.7 ± 0.7 | 0.27 ± 0.07 | 0.3 ± 0.2 | 17.1 ± 0.6 | 19 ± 3 | 6 ± 1 | 9 ± 3 | 19 ± 1 | 23.49 ± 0.07 |
| DBT-4but | 1.4 ± 0.3 | 0.19 ± 0.06 | 0.28 ± 0.09 | 6.2 ± 0.4 | 19 ± 4 | 4.3 ± 0.2 | 7 ± 2 | 10.6 ± 0.9 | 14.1 ± 0.4 |
| PHE | 16 ± 2 | 0.6 ± 0.2 | 0.24 ± 0.08 | 104 ± 3 | 26 ± 3 | 50 ± 7 | 61 ± 14 | 117 ± 3 | 118 ± 2 |
| ANT | <LOQ | 0.1 ± 0.2 | <LOQ | <LOQ | <LOQ | <LOQ | <LOQ | <LOQ | <LOQ |
| PHE-3 | 7 ± 2 | 0.1 ± 0.1 | <LOQ | 65 ± 1 | 21 ± 2 | 32 ± 7 | 37 ± 13 | 87 ± 5 | 85 ± 2 |
| PHE-2 | 7 ± 2 | 0.4 ± 0.2 | <LOQ | 63 ± 1 | 20 ± 3 | 33 ± 7 | 37 ± 13 | 85 ± 5 | 81 ± 2 |
| PHE-9 | 17 ± 3 | 0.97 ± 0.03 | 0.6 ± 0.2 | 111 ± 4 | 38 ± 4 | 41 ± 9 | 49 ± 17 | 119 ± 6 | 127 ± 3 |
| PHE-1 | 7 ± 2 | 0.17 ± 0.04 | 0.04 ± 0.07 | 55 ± 2 | 19 ± 3 | 31 ± 6 | 36 ± 10 | 79 ± 5 | 71 ± 2 |
| PHE-3,6 | 7 ± 2 | 0.6 ± 0.2 | 0.4 ± 0.3 | 35.7 ± 0.7 | 27 ± 4 | 19 ± 4 | 23 ± 9 | 52 ± 4 | 53.4 ± 0.7 |
| PHE-1,7 | 6 ± 2 | 0.4 ± 0.2 | 0.2 ± 0.2 | 24 ± 1 | 39 ± 7 | 25 ± 5 | 28 ± 12 | 45 ± 4 | 40.9 ± 0.6 |
| PHE-1,2 | 1.2 ± 0.4 | <LOQ | <LOQ | 4.2 ± 0.3 | 6 ± 1 | 5 ± 1 | 6 ± 3 | 10.5 ± 0.4 | 9.5 ± 0.4 |
| PHE-2,6,9 | 7 ± 2 | 0.7 ± 0.2 | 0.7 ± 0.2 | 24 ± 4 | 33 ± 13 | 27 ± 4 | 32 ± 9 | 45 ± 3 | 45 ± 3 |
| PHE-1,2,6 | 1 ± 0.3 | 0.04 ± 0.02 | 0.05 ± 0.02 | 3.2 ± 0.3 | 6 ± 1 | 4 ± 0.7 | 5 ± 1 | 7.2 ± 0.6 | 6.5 ± 0.2 |
| PHE-1,2,7 | 0.4 ± 0.1 | <LOQ | <LOQ | 0.7 ± 0.1 | 2.4 ± 0.5 | 1.5 ± 0.3 | 1.7 ± 0.5 | 2.6 ± 0.2 | 2.44 ± 0.09 |
| PHE-1,2,6,9 | 0.6 ± 0.1 | 0.07 ± 0.04 | 0.08 ± 0.04 | 1.9 ± 0.4 | 5 ± 1 | 1.9 ± 0.2 | 2.8 ± 0.5 | 4 ± 0.2 | 4.3 ± 0.2 |
| FLA | 1.7 ± 0.3 | 0.16 ± 0.03 | 0.19 ± 0.04 | 13.6 ± 0.7 | 5.8 ± 0.6 | 6 ± 1 | 7 ± 3 | 19 ± 2 | 17.9 ± 0.4 |
| PYR | 1.9 ± 0.4 | 0.21 ± 0.04 | 0.3 ± 0.07 | 11.6 ± 0.3 | 9 ± 1 | 7 ± 2 | 9 ± 4 | 20 ± 1 | 19.1 ± 0.4 |
| FLA-2 | 0.4 ± 0.2 | 0.12 ± 0.21 | <LOQ | 3.6 ± 0.3 | <LOQ | 2.7 ± 0.5 | 3 ± 1 | 6.8 ± 0.5 | 5.67 ± 0.06 |
| PYR-1 | 1.2 ± 0.3 | 0.22 ± 0.05 | 0.29 ± 0.05 | 3.3 ± 0.3 | 5 ± 1 | 3.9 ± 0.8 | 4 ± 1 | 6.8 ± 0.4 | 6.7 ± 0.1 |
| PYR-4,5 | <LOQ | <LOQ | <LOQ | <LOQ | <LOQ | <LOQ | <LOQ | <LOQ | <LOQ |
| PYR-1pro | <LOQ | <LOQ | <LOQ | 0.4 ± 0.1 | <LOQ | <LOQ | <LOQ | <LOQ | <LOQ |
| PYR-1et | <LOQ | <LOQ | <LOQ | 1 ± 0.2 | 1.8 ± 0.3 | 0.53 ± 0.07 | 0.8 ± 0.1 | 1.3 ± 0.1 | 1.5 ± 0.4 |
| PYR-1but | <LOQ | <LOQ | <LOQ | 0.4 ± 0.1 | 1.6 ± 0.5 | <LOQ | <LOQ | <LOQ | <LOQ |
| BAA | 0.4 ± 0.1 | <LOQ | <LOQ | 1.3 ± 0.2 | 2.2 ± 0.4 | 1.8 ± 0.4 | 2 ± 1 | 3.3 ± 0.3 | 2.86 ± 0.09 |
| CHR | 2.9 ± 0.8 | 0.24 ± 0.05 | 0.2 ± 0.2 | 16 ± 2 | 12 ± 3 | 10 ± 2 | 12 ± 4 | 28 ± 4 | 23.9 ± 0.1 |
| CHR-1 | 0.3 ± 0.1 | <LOQ | <LOQ | 1.4 ± 0.2 | 6 ± 2 | 1.9 ± 0.2 | 2.7 ± 0.9 | 4.1 ± 0.6 | 4.4 ± 0.4 |
| CHR-6et | <LOQ | <LOQ | <LOQ | <LOQ | <LOQ | <LOQ | <LOQ | <LOQ | <LOQ |
| CHR-6pro | <LOQ | <LOQ | <LOQ | <LOQ | <LOQ | <LOQ | <LOQ | <LOQ | <LOQ |
| CHR-6but | <LOQ | <LOQ | <LOQ | <LOQ | <LOQ | <LOQ | <LOQ | <LOQ | <LOQ |
| BBF | 0.52 ± 0.09 | 0.1 ± 0.03 | 0.14 ± 0.02 | 2.8 ± 0.4 | 10 ± 2 | 2.6 ± 0.2 | 4 ± 1 | 5 ± 1 | 6.5 ± 0.2 |
| BKF | 0.31 ± 0.05 | <LOQ | <LOQ | 1 ± 0.1 | 4.7 ± 0.6 | 1.4 ± 0.1 | 2 ± 0.6 | 2.5 ± 0.1 | 3 ± 0.4 |
| BEP | 1 ± 0.3 | 0.08 ± 0.04 | 0.13 ± 0.07 | 5 ± 0.3 | 10 ± 2 | 3.7 ± 0.4 | 5 ± 2 | 9 ± 1 | 9.1 ± 0.4 |
| BAP | 0.04 ± 0.07 | <LOQ | <LOQ | 1.4 ± 0.3 | 2 ± 1 | 1.2 ± 0.1 | 1.7 ± 0.7 | 2.4 ± 0.4 | 2.4 ± 0.1 |
| PER | 0.14 ± 0.03 | <LOQ | <LOQ | 0.41 ± 0.08 | 3 ± 2 | 0.54 ± 0.05 | 0.9 ± 0.4 | 1.2 ± 0.3 | 2 ± 1 |
| IND | <LOQ | <LOQ | <LOQ | 0.34 ± 0.06 | 1.4 ± 0.4 | <LOQ | 0.2 ± 0.2 | 0.4 ± 0.3 | 0.79 ± 0.09 |
| DBA | <LOQ | <LOQ | <LOQ | 0.5 ± 0.1 | 2.2 ± 0.3 | 0.54 ± 0.01 | 0.8 ± 0.3 | 1.6 ± 0.2 | 1.8 ± 0.2 |
| BGP | 0.28 ± 0.06 | <LOQ | <LOQ | 1.8 ± 0.5 | 5 ± 1 | 1.4 ± 0.1 | 1.9 ± 0.2 | 3.3 ± 0.3 | 4 ± 0.3 |
| C1-BT | 0.12 ± 0.01 | NA | NA | 0.12 ± 0.01 | 0.22 ± 0.01 | NA | NA | NA | 0.52 ± 0.03 |
| C2-BT | 0.21 ± 0.08 | NA | NA | 2.2 ± 0.1 | 1.6 ± 0.1 | NA | NA | NA | 7.46 ± 0.09 |
| C3-BT | 5.9 ± 0.3 | NA | NA | 18 ± 1 | 10.8 ± 0.4 | NA | NA | NA | 28.4 ± 0.5 |
| C4-BT | 6.1 ± 0.7 | NA | NA | 24.1 ± 0.6 | 16 ± 1 | NA | NA | NA | 29 ± 1 |
| C1-NAP | 23.9 ± 0.6 | NA | NA | 45 ± 2 | 41 ± 2 | NA | NA | NA | 159 ± 3 |
| C2-NAP | 100 ± 5 | NA | NA | 288 ± 7 | 210 ± 4 | NA | NA | NA | 675 ± 8 |
| C3-NAP | 163 ± 5 | NA | NA | 871 ± 33 | 401 ± 28 | NA | NA | NA | 1232 ± 23 |
| C4-NAP | 123 ± 8 | NA | NA | 707 ± 27 | 390 ± 35 | NA | NA | NA | 819 ± 3 |
| C1-FLU | 24 ± 3 | NA | NA | 164 ± 6 | 49 ± 3 | NA | NA | NA | 249 ± 7 |
| C2-FLU | 35 ± 5 | NA | NA | 206 ± 7 | 106 ± 13 | NA | NA | NA | 270 ± 6 |
| C3-FLU | 37 ± 2 | NA | NA | 123 ± 4 | 146 ± 21 | NA | NA | NA | 180 ± 8 |
| C1-DBT | 13 ± 2 | NA | NA | 118.2 ± 0.3 | 34 ± 3 | NA | NA | NA | 162 ± 3 |
| C2-DBT | 41 ± 11 | NA | NA | 281 ± 2 | 184 ± 32 | NA | NA | NA | 421 ± 3 |
| C3-DBT | 35 ± 10 | NA | NA | 170 ± 9 | 286 ± 57 | NA | NA | NA | 331 ± 11 |
| C4-DBT | 38 ± 7 | NA | NA | 153 ± 11 | 489 ± 95 | NA | NA | NA | 375 ± 15 |
| C1-PHE | 38 ± 9 | NA | NA | 294 ± 7 | 98 ± 12 | NA | NA | NA | 364 ± 8 |
| C2-PHE | 70 ± 21 | NA | NA | 362 ± 19 | 362 ± 64 | NA | NA | NA | 673 ± 25 |
| C3-PHE | 57 ± 13 | NA | NA | 218 ± 32 | 451 ± 87 | NA | NA | NA | 478 ± 13 |
| C4-PHE | 83 ± 12 | NA | NA | 215 ± 25 | 686 ± 141 | NA | NA | NA | 531 ± 25 |
| C1-PYR | 23 ± 6 | NA | NA | 70 ± 2 | 127 ± 25 | NA | NA | NA | 155 ± 2 |
| C2-PYR | 26 ± 6 | NA | NA | 83 ± 8 | 265 ± 61 | NA | NA | NA | 223 ± 5 |
| C3-PYR | 28 ± 6 | NA | NA | 105 ± 15 | 486 ± 105 | NA | NA | NA | 339 ± 17 |
| C1-CHR | 10 ± 3 | NA | NA | 35 ± 6 | 127 ± 24 | NA | NA | NA | 108 ± 10 |
| C2-CHR | 8 ± 1 | NA | NA | 29 ± 6 | 153 ± 35 | NA | NA | NA | 103 ± 11 |
| C3-CHR | 7 ± 1 | NA | NA | 37 ± 9 | 279 ± 57 | NA | NA | NA | 181 ± 15 |
| C4-CHR | 7.84 ± 0.55 | NA | NA | 80 ± 25 | 427 ± 79 | NA | NA | NA | 257 ± 25 |
| ΣPAH | 345 ± 30 | 18 ± 1 | 10 ± 2 | 1628 ± 39 | 836 ± 86 | 947 ± 151 | 1134 ± 287 | 2209 ± 9 | 2276 ± 42 |
| tPAH | 1068 ± 125 |  |  | 4959 ± 199 | 5983 ± 991 |  |  |  | 8790 ± 68 |

Table S7 continued.

| **Body burden (pg/embryo)** | **Haddock**  **8.6 µg/L Day 5** | **Haddock**  **8.6 µg/L Day 7** | **Haddock**  **8.6 µg/L Day 9** | **Haddock**  **8.6 µg/L Day 10** | **Haddock**  **8.6 µg/L**  **Day 10 Embryo** | **Haddock**  **8.6 µg/L**  **Day 10 Chorion** | **Haddock WSF**  **Day 1** | **Haddock WSF**  **Day 2** | **Haddock WSF**  **Day 3** |
| --- | --- | --- | --- | --- | --- | --- | --- | --- | --- |
| BIP | 51.4 ± 0.7 | 48 ± 2 | 42 ± 2 | 21 ± 3 | 1.7 | 0.7 | 12 ± 1 | 12 ± 0.2 | 14 ± 2 |
| BT | <LOQ | <LOQ | 0.03 ± 0.01 | 0.26 ± 0.02 | 0.22 | 0.26 | <LOQ | <LOQ | <LOQ |
| BT-2,5 | 1.87 ± 0.02 | 1.72 ± 0.07 | 1.51 ± 0.06 | 1 ± 0.2 | 0.19 | 0.15 | 0.35 ± 0.04 | 0.37 ± 0.01 | 0.44 ± 0.02 |
| BT-2,5,7 | 4.3 ± 0.2 | 3.8 ± 0.1 | 3.6 ± 0.1 | 1.9 ± 0.2 | 0.44 | 0.71 | 0.54 ± 0.08 | 0.8 ± 0.1 | 1.1 ± 0.08 |
| NAP | 11.2 ± 0.7 | 10.5 ± 0.7 | 8.6 ± 0.2 | 12 ± 4 | 5.8 | 5 | 2.2 ± 0.4 | 2 ± 0.3 | 3.3 ± 0.4 |
| NAP-2 | 111 ± 2 | 108 ± 5 | 90 ± 5 | 53 ± 12 | 6.8 | 3.9 | 25 ± 2 | 24.6 ± 0.5 | 30 ± 4 |
| NAP-1 | 49 ± 1 | 47 ± 3 | 40 ± 2 | 22 ± 5 | 1.8 | 1.3 | 10.1 ± 0.5 | 9.8 ± 0.6 | 13 ± 2 |
| NAP-2,6&2,7 | 195 ± 2 | 182 ± 7 | 156 ± 5 | 117 ± 8 | 25 | 27 | 47 ± 4 | 50 ± 2 | 59 ± 4 |
| NAP-1,4 | 161 ± 1 | 151 ± 5 | 132 ± 5 | 97 ± 5 | 21 | 24 | 35 ± 3 | 41 ± 1 | 49 ± 4 |
| NAP-1,3&2,3 | 107.4 ± 0.6 | 99 ± 4 | 91 ± 3 | 65 ± 3 | 14 | 16 | 24 ± 2 | 26.4 ± 0.8 | 31 ± 3 |
| NAP-1,3,7 | 94 ± 2 | 76 ± 2 | 78 ± 7 | 47 ± 4 | 7.9 | 23 | 19.6 ± 0.7 | 25 ± 1 | 32 ± 1 |
| NAP-2,3,5 | 327 ± 7 | 298 ± 12 | 281 ± 13 | 194 ± 28 | 45 | 94 | 54 ± 3 | 72 ± 1 | 91 ± 1 |
| NAP-1,2,3 | 19.02 ± 0.55 | 16 ± 0.4 | 18 ± 2 | 13 ± 2 | 2.7 | 5.3 | 1.8 ± 0.1 | 2.6 ± 0.06 | 3.3 ± 0.1 |
| NAP-1,2,5,6 | 51 ± 2 | 49 ± 2 | 55 ± 5 | 23 ± 7 | 3.2 | 38 | 4.8 ± 0.2 | 6.6 ± 0.1 | 8.96 ± 0.06 |
| NAP-1,4,6,7 | 58 ± 3 | 50 ± 3 | 54 ± 7 | 20 ± 5 | 2.4 | 56 | 7.5 ± 0.4 | 7.68 ± 0.07 | 9.2 ± 0.6 |
| ACY | 0.1 ± 0.01 | 0.03 ± 0.01 | 0.04 ± 0.02 | <LOQ | <LOQ | <LOQ | 0.01 ± 0.01 | <LOQ | 0.01 ± 0.01 |
| ACE | 7.1 ± 0.4 | 5.5 ± 0.6 | 5.4 ± 0.3 | 2.66 ± 0.01 | 1 | 1.1 | 1.18 ± 0.09 | 1.45 ± 0.02 | 2 ± 0.5 |
| DBF | 26.7 ± 0.3 | 23.6 ± 0.9 | 21.2 ± 0.6 | 11.9 ± 0.3 | 0.8 | 0.7 | 5.9 ± 0.6 | 5.8 ± 0.04 | 7 ± 1 |
| FLU | 66 ± 2 | 61 ± 2 | 55 ± 1 | 27.9 ± 0.8 | 4.8 | 9.2 | 14 ± 1 | 13.8 ± 0.5 | 17 ± 2 |
| FLU-9et | 6.2 ± 0.2 | 6.5 ± 0.2 | 7.9 ± 0.7 | 6 ± 2 | 1.2 | 3.5 | 0.4 ± 0.01 | 0.68 ± 0.01 | 1.03 ± 0.05 |
| FLU-1 | 117 ± 1 | 102 ± 7 | 97 ± 6 | 56 ± 9 | 13 | 53 | 19.9 ± 0.8 | 24.27 ± 0.03 | 23 ± 7 |
| FLU-9pro | 2.8 ± 0.1 | 3.5 ± 0.2 | 4.3 ± 0.2 | 2 ± 1 | 0.5 | 3.2 | 0.09 ± 0.04 | 0.17 ± 0.02 | 0.36 ± 0.08 |
| DBT | 76 ± 3 | 70 ± 3 | 65 ± 3 | 18 ± 7 | <LOQ | 5.8 | 12.5 ± 0.8 | 15.2 ± 0.6 | 20 ± 3 |
| DBT-4 | 75 ± 2 | 65 ± 5 | 66 ± 5 | 36 ± 9 | 7.7 | 48 | 10.5 ± 0.4 | 13 ± 0.3 | 16 ± 1 |
| DBT-4et | 35 ± 2 | 34 ± 2 | 37 ± 5 | 14 ± 3 | 3.1 | 45 | 3.3 ± 0.1 | 3.74 ± 0.06 | 4.4 ± 0.3 |
| DBT-4pro | 30 ± 2 | 35 ± 2 | 41 ± 8 | 9 ± 3 | 3.1 | 61 | 2.38 ± 0.02 | 2.68 ± 0.02 | 3.2 ± 0.3 |
| DBT-4but | 19 ± 2 | 25 ± 2 | 31 ± 8 | 4 ± 1 | 2.1 | 54 | 0.78 ± 0.03 | 0.6 ± 0.02 | 0.68 ± 0.03 |
| PHE | 130.3 ± 0.7 | 119 ± 6 | 106 ± 6 | 70 ± 12 | 12 | 36 | 26 ± 2 | 32 ± 0.4 | 37 ± 3 |
| ANT | <LOQ | <LOQ | <LOQ | <LOQ | <LOQ | <LOQ | <LOQ | <LOQ | <LOQ |
| PHE-3 | 88 ± 5 | 75 ± 4 | 76 ± 8 | 28 ± 3 | 5.6 | 61 | 14.8 ± 0.3 | 13.1 ± 0.2 | 14 ± 1 |
| PHE-2 | 79 ± 5 | 68 ± 4 | 72 ± 8 | 29 ± 4 | 6.2 | 69 | 15.4 ± 0.4 | 13.8 ± 0.4 | 15 ± 2 |
| PHE-9 | 150 ± 5 | 140 ± 8 | 141 ± 10 | 67 ± 14 | 16 | 99 | 22.1 ± 0.6 | 28.5 ± 0.8 | 36 ± 3 |
| PHE-1 | 71 ± 4 | 64 ± 5 | 69 ± 9 | 27 ± 3 | 6.1 | 64 | 15.3 ± 0.4 | 13.2 ± 0.6 | 14 ± 2 |
| PHE-3,6 | 62 ± 4 | 65 ± 4 | 75 ± 12 | 22 ± 3 | 5.8 | 89 | 8.3 ± 0.1 | 6.69 ± 0.09 | 7.9 ± 0.8 |
| PHE-1,7 | 50 ± 4 | 68 ± 6 | 78 ± 9 | 18.8 ± 0.2 | 7.5 | 112 | 5.7 ± 0.1 | 1.7 ± 0.2 | 2.5 ± 0.3 |
| PHE-1,2 | 12 ± 1 | 13 ± 1 | 17 ± 4 | 3.7 ± 0.7 | 1.1 | 25 | 1.27 ± 0.09 | 0.35 ± 0.05 | 0.5 ± 0.1 |
| PHE-2,6,9 | 60 ± 4 | 74 ± 7 | 76 ± 21 | 28 ± 2 | 13 | 185 | 5.2 ± 0.2 | 1.7 ± 0.1 | 3 ± 0.3 |
| PHE-1,2,6 | 8.6 ± 0.6 | 12 ± 1 | 13 ± 3 | 3.7 ± 0.4 | 1.7 | 35 | 0.49 ± 0.05 | 0.08 ± 0.01 | 0.18 ± 0.02 |
| PHE-1,2,7 | 3.3 ± 0.2 | 4.6 ± 0.4 | 5 ± 1 | 1.5 ± 0.2 | 0.69 | 15 | 0.27 ± 0.01 | 0.11 ± 0 | 0.18 ± 0.02 |
| PHE-1,2,6,9 | 5.8 ± 0.4 | 8.7 ± 0.6 | 9 ± 3 | 1.7 ± 0.2 | 1.2 | 24 | 0.36 ± 0.05 | 0.13 ± 0.03 | 0.17 ± 0.05 |
| FLA | 20 ± 1 | 16.5 ± 0.9 | 18 ± 2 | 6 ± 2 | 0.36 | 17 | 2.9 ± 0.3 | 2.57 ± 0.02 | 2.4 ± 0.2 |
| PYR | 21 ± 2 | 20 ± 1 | 26 ± 4 | 7 ± 1 | 0.89 | 25 | 2.57 ± 0.08 | 1.47 ± 0.08 | 1.7 ± 0.2 |
| FLA-2 | 6.7 ± 0.3 | 7 ± 1 | 7 ± 1 | 2.8 ± 0.2 | 0.91 | 20 | 0.98 ± 0.04 | 0.41 ± 0.07 | 0.4 ± 0.2 |
| PYR-1 | 7.1 ± 0.6 | 9 ± 1 | 9 ± 3 | 3.8 ± 0.7 | 1.5 | 32 | 0.8 ± 0.1 | 0.28 ± 0.06 | 0.5 ± 0.1 |
| PYR-4,5 | <LOQ | <LOQ | <LOQ | <LOQ | <LOQ | <LOQ | <LOQ | <LOQ | <LOQ |
| PYR-1pro | <LOQ | <LOQ | <LOQ | <LOQ | <LOQ | <LOQ | <LOQ | <LOQ | <LOQ |
| PYR-1et | 3.1 ± 0.2 | 4.4 ± 0.5 | 4 ± 1 | 1.1 ± 0.2 | 0.57 | 15 | <LOQ | <LOQ | <LOQ |
| PYR-1but | <LOQ | <LOQ | 2.5 ± 0.6 | 0.37 ± 0.06 | 0.34 | 5.9 | <LOQ | <LOQ | <LOQ |
| BAA | 3.8 ± 0.3 | 4.6 ± 0.3 | 5 ± 1 | 1.1 ± 0.3 | 0.26 | 11 | 4.4 ± 0.3 | 4 ± 0.3 | 5 ± 0.4 |
| CHR | 29 ± 1 | 34 ± 4 | 32 ± 6 | 10.8 ± 0.4 | 3.4 | 61 | 3.3 ± 0.2 | 3 ± 0.2 | 3.2 ± 0.4 |
| CHR-1 | 5.7 ± 0.6 | 8 ± 1 | 11 ± 3 | 2 ± 0.5 | 0.64 | 22 | 0.18 ± 0.03 | 0.08 ± 0.01 | 0.14 ± 0.02 |
| CHR-6et | <LOQ | <LOQ | <LOQ | <LOQ | <LOQ | <LOQ | <LOQ | <LOQ | <LOQ |
| CHR-6pro | <LOQ | <LOQ | <LOQ | <LOQ | <LOQ | <LOQ | <LOQ | <LOQ | <LOQ |
| CHR-6but | <LOQ | <LOQ | <LOQ | <LOQ | <LOQ | <LOQ | <LOQ | <LOQ | <LOQ |
| BBF | 9 ± 2 | 12 ± 1 | 16 ± 5 | 2.6 ± 0.3 | 1.6 | 33 | 0.26 ± 0.01 | 0.12 ± 0.02 | 0.21 ± 0.03 |
| BKF | 4.1 ± 0.6 | 5 ± 1 | 7 ± 3 | 0.5 ± 0.1 | 0.17 | 12 | 0.15 ± 0.01 | 0.04 ± 0.01 | 0.11 ± 0.02 |
| BEP | 12.6 ± 0.6 | 16 ± 0.6 | 18 ± 5 | 2.8 ± 0.4 | 1.3 | 32 | 0.64 ± 0.03 | 0.32 ± 0.03 | 0.49 ± 0.09 |
| BAP | 3.7 ± 0.2 | 4.3 ± 0.5 | 6 ± 2 | 0.1 ± 0.1 | <LOQ | 10 | <LOQ | <LOQ | <LOQ |
| PER | 2.3 ± 0.3 | 3 ± 0.3 | 4 ± 2 | 0.37 ± 0.05 | 0.24 | 11 | <LOQ | <LOQ | 0.09 ± 0.02 |
| IND | 1.4 ± 0.4 | 1.7 ± 0.3 | 1.9 ± 0.8 | 0.06 ± 0.04 | 0.05 | 2.8 | <LOQ | <LOQ | <LOQ |
| DBA | 2.6 ± 0.6 | 3.5 ± 0.3 | 4 ± 1 | 0.01 ± 0.01 | <LOQ | 4.8 | <LOQ | <LOQ | <LOQ |
| BGP | 5.3 ± 0.5 | 7.2 ± 0.5 | 8 ± 3 | 0.5 ± 0.2 | 0.32 | 13 | <LOQ | <LOQ | 0.2 ± 0.1 |
| C1-BT | NA | NA | 0.47 ± 0.02 | 0.63 ± 0.04 | 0.44 | 0.22 | NA | NA | 0.14 ± 0.01 |
| C2-BT | NA | NA | 6.3 ± 0.4 | 0.2 ± 0.3 | <LOQ | <LOQ | NA | NA | 0.4 ± 0.1 |
| C3-BT | NA | NA | 31 ± 1 | <LOQ | <LOQ | <LOQ | NA | NA | 8.6 ± 0.8 |
| C4-BT | NA | NA | 43 ± 1 | 14 ± 5 | 0.82 | 14 | NA | NA | 7.7 ± 0.2 |
| C1-NAP | NA | NA | 130 ± 6 | 75 ± 17 | 8.6 | 5.3 | NA | NA | 43 ± 5 |
| C2-NAP | NA | NA | 578 ± 29 | 367 ± 9 | 70 | 72 | NA | NA | 173 ± 11 |
| C3-NAP | NA | NA | 1177 ± 67 | 705 ± 75 | 130 | 260 | NA | NA | 313.9 ± 0.5 |
| C4-NAP | NA | NA | 1070 ± 111 | 500 ± 137 | 83 | 612 | NA | NA | 170 ± 5 |
| C1-FLU | NA | NA | 205 ± 22 | 96 ± 19 | 19 | 81 | NA | NA | 50 ± 3 |
| C2-FLU | NA | NA | 343 ± 91 | 154 ± 40 | 32 | 399 | NA | NA | 40 ± 3 |
| C3-FLU | NA | NA | 348 ± 64 | 111 ± 23 | 31 | 467 | NA | NA | 23 ± 2 |
| C1-DBT | NA | NA | 153 ± 15 | 152 ± 24 | 34 | 294 | NA | NA | 30 ± 1 |
| C2-DBT | NA | NA | 543 ± 96 | 248 ± 9 | 79 | 1 437 | NA | NA | 50 ± 3 |
| C3-DBT | NA | NA | 633 ± 142 | 147 ± 7 | 64 | 1 750 | NA | NA | 23 ± 2 |
| C4-DBT | NA | NA | 863 ± 249 | 136 ± 33 | 74 | 2 205 | NA | NA | 19 ± 2 |
| C1-PHE | NA | NA | 359 ± 33 | 74 ± 21 | 13 | 95 | NA | NA | 79 ± 8 |
| C2-PHE | NA | NA | 1013 ± 194 | 161 ± 27 | 38 | 713 | NA | NA | 59 ± 7 |
| C3-PHE | NA | NA | 986 ± 228 | 110 ± 20 | 43 | 1 154 | NA | NA | 30 ± 2 |
| C4-PHE | NA | NA | 1194 ± 307 | 98 ± 23 | 59 | 1 656 | NA | NA | 30 ± 3 |
| C1-PYR | NA | NA | 272 ± 48 | 47 ± 7 | 15 | 418 | NA | NA | 13 ± 2 |
| C2-PYR | NA | NA | 525 ± 130 | 52 ± 4 | 26 | 829 | NA | NA | 11 ± 2 |
| C3-PYR | NA | NA | 845 ± 249 | 54 ± 11 | 35 | 1 262 | NA | NA | 12 ± 2 |
| C1-CHR | NA | NA | 259 ± 83 | 18 ± 1 | 9 | 441 | NA | NA | 3 ± 0.4 |
| C2-CHR | NA | NA | 246 ± 82 | 11 ± 3 | 7.3 | 433 | NA | NA | 2.1 ± 0.4 |
| C3-CHR | NA | NA | 422 ± 127 | 17 ± 6 | 14 | 786 | NA | NA | 4 ± 2 |
| C4-CHR | NA | NA | 619 ± 189 | 27 ± 9 | 21 | 1 147 | NA | NA | 2 ± 2 |
| ΣPAH | 2561 ± 44 | 2439 ± 98 | 2402 ± 204 | 1219 ± 74 | 266 | 1722 | 445 ± 23 | 486 ± 4 | 584 ± 45 |
| tPAH |  |  | 13315 ± 2532 | 3796 ± 500 | 984 | 17 119 |  |  | 1309 ± 70 |

Table S7 continued.

| **Body burden (pg/embryo)** | **Haddock WSF Day 5** | **Haddock WSF Day 7** | **Haddock WSF Day 9** | **Haddock WSF Day 10** | **Haddock WSF Day 11** | **Haddock WSF Day 12** |
| --- | --- | --- | --- | --- | --- | --- |
| BIP | 14 ± 1 | 12.2 ± 0.5 | 9.4 ± 0.4 | 5 ± 0.4 | 0.3 ± 0.2 | <LOQ |
| BT | <LOQ | <LOQ | 0 ± 0.01 | <LOQ | <LOQ | <LOQ |
| BT-2,5 | 0.44 ± 0.04 | 0.3 ± 0.2 | 0.31 ± 0.01 | 0.16 ± 0.02 | <LOQ | <LOQ |
| BT-2,5,7 | 1.11 ± 0.04 | 0.98 ± 0.02 | 0.7 ± 0.1 | 0.45 ± 0.09 | <LOQ | <LOQ |
| NAP | 2.7 ± 0.3 | 2 ± 1 | 2 ± 1 | 0.4 ± 0.3 | <LOQ | <LOQ |
| NAP-2 | 29 ± 2 | 25 ± 1 | 19 ± 0.9 | 9.3 ± 0.8 | 0.2 ± 0.3 | 0.04 ± 0.07 |
| NAP-1 | 12.2 ± 0.8 | 10.1 ± 0.5 | 7.3 ± 0.3 | 3.8 ± 0.5 | <LOQ | 0.05 ± 0.06 |
| NAP-2,6&2,7 | 57 ± 4 | 51 ± 2 | 33 ± 2 | 21 ± 1 | 1.7 ± 0.2 | 0.54 ± 0.03 |
| NAP-1,4 | 48 ± 3 | 45 ± 1 | 30 ± 1 | 19 ± 1 | 1.9 ± 0.2 | 0.5 ± 0.1 |
| NAP-1,3&2,3 | 32 ± 2 | 29 ± 2 | 19.5 ± 0.9 | 13.1 ± 0.7 | 1.7 ± 0.2 | 0.75 ± 0.03 |
| NAP-1,3,7 | 29 ± 3 | 24.7 ± 0.8 | 7.8 ± 0.6 | 7.7 ± 0.5 | 0.49 ± 0.08 | 0.29 ± 0.02 |
| NAP-2,3,5 | 96 ± 8 | 87 ± 12 | 43 ± 3 | 34 ± 3 | ± 1 | 2 ± 0.2 |
| NAP-1,2,3 | 4 ± 0.5 | 3.9 ± 0.8 | 2.1 ± 0.2 | 1.4 ± 0.1 | 0.12 ± 0.02 | <LOQ |
| NAP-1,2,5,6 | 10 ± 1 | 8.5 ± 0.2 | 4.7 ± 0.5 | 3.6 ± 0.2 | 0.6 ± 0.08 | 0.23 ± 0.05 |
| NAP-1,4,6,7 | 7 ± 1 | 5.5 ± 0.3 | 2.9 ± 0.4 | 2 ± 0.1 | 0.2 ± 0.03 | 0.16 ± 0.06 |
| ACY | 0.02 ± 0.01 | 0 ± 0.01 | 0.01 ± 0.01 | 0.01 ± 0.01 | <LOQ | <LOQ |
| ACE | 1.7 ± 0.2 | 1.66 ± 0.02 | 1.3 ± 0.2 | 0.78 ± 0.06 | 0.27 ± 0.04 | 0.11 ± 0.03 |
| DBF | 6.8 ± 0.6 | 6.2 ± 0.3 | 4.4 ± 0.2 | 2.8 ± 0.2 | 0.18 ± 0.08 | <LOQ |
| FLU | 15 ± 1 | 13 ± 1 | 8.9 ± 0.4 | 6.1 ± 0.5 | 0.6 ± 0.2 | 0.18 ± 0.07 |
| FLU-9et | 1.6 ± 0.2 | 1.8 ± 0.3 | 1.38 ± 0.09 | 0.97 ± 0.09 | 0.43 ± 0.08 | 0.19 ± 0.01 |
| FLU-1 | 23 ± 3 | 16.7 ± 0.6 | 8.4 ± 0.5 | 6.9 ± 0.6 | <LOQ | <LOQ |
| FLU-9pro | 0.66 ± 0.05 | 0.81 ± 0.03 | 0.71 ± 0.01 | 0.4 ± 0.1 | 0.28 ± 0.06 | 0.15 ± 0.02 |
| DBT | 17 ± 1 | 12 ± 1 | 6.3 ± 0.3 | 4.9 ± 0.3 | 0.33 ± 0.03 | 0.09 ± 0.01 |
| DBT-4 | 12 ± 2 | 8.4 ± 0.3 | 3.6 ± 0.6 | 2.9 ± 0.2 | 0.22 ± 0.03 | 0.2 ± 0.1 |
| DBT-4et | 3.1 ± 0.7 | 2.4 ± 0.1 | 1.3 ± 0.3 | 0.74 ± 0.04 | 0.17 ± 0.02 | 0.2 ± 0.1 |
| DBT-4pro | 2.6 ± 0.5 | 2.1 ± 0.2 | 1.1 ± 0.3 | 0.4 ± 0.3 | 0.15 ± 0.03 | 0.2 ± 0.1 |
| DBT-4but | 0.8 ± 0.2 | 0.7 ± 0.1 | 0.4 ± 0.1 | 0.16 ± 0.02 | 0.07 ± 0.01 | 0.1 ± 0.04 |
| PHE | 30 ± 2 | 21 ± 1 | 9.2 ± 0.6 | 7 ± 0.4 | 0.5 ± 0.3 | 0.4 ± 0.3 |
| ANT | <LOQ | <LOQ | <LOQ | <LOQ | <LOQ | <LOQ |
| PHE-3 | 8 ± 2 | 6 ± 1 | 3 ± 0.7 | 1.3 ± 0.2 | 0.05 ± 0.05 | 0.1 ± 0.1 |
| PHE-2 | 7 ± 1 | 4.5 ± 0.9 | 2.4 ± 0.4 | 1.4 ± 0.1 | 0.01 ± 0.02 | <LOQ |
| PHE-9 | 29 ± 3 | 21 ± 1 | 10 ± 2 | 7.4 ± 0.5 | 0.84 ± 0.09 | 0.8 ± 0.3 |
| PHE-1 | 7 ± 2 | 5.1 ± 0.5 | 2.6 ± 0.5 | 1.4 ± 0.3 | 0.11 ± 0.02 | 0.1 ± 0.1 |
| PHE-3,6 | 6 ± 1 | 5.2 ± 0.7 | 2.8 ± 0.8 | 1.64 ± 0.07 | 0.32 ± 0.03 | 0.4 ± 0.2 |
| PHE-1,7 | 2 ± 1 | 2.2 ± 0.8 | 1.4 ± 0.4 | 0.5 ± 0.2 | 0.11 ± 0.01 | 0.1 ± 0.07 |
| PHE-1,2 | 0.3 ± 0.1 | 0.4 ± 0.1 | 0.25 ± 0.08 | 0.12 ± 0.01 | <LOQ | <LOQ |
| PHE-2,6,9 | 2.6 ± 0.8 | 2.9 ± 0.7 | 1 ± 0.2 | 0.8 ± 0.1 | 0.26 ± 0.05 | 0.2 ± 0.1 |
| PHE-1,2,6 | 0.18 ± 0.08 | 0.2 ± 0.2 | 0.14 ± 0.05 | 0.04 ± 0.02 | <LOQ | <LOQ |
| PHE-1,2,7 | 0.18 ± 0.05 | 0.1 ± 0.1 | 0.04 ± 0.02 | <LOQ | <LOQ | <LOQ |
| PHE-1,2,6,9 | 0.19 ± 0.04 | 0.23 ± 0.06 | 0.1 ± 0.04 | <LOQ | <LOQ | <LOQ |
| FLA | 1.7 ± 0.6 | 1.4 ± 0.2 | 1 ± 0.3 | 0.46 ± 0.05 | 0.1 ± 0.01 | 0.1 ± 0.06 |
| PYR | 1.3 ± 0.4 | 1.2 ± 0.1 | 1.3 ± 0.2 | 0.44 ± 0.03 | 0.14 ± 0.05 | 0.1 ± 0.04 |
| FLA-2 | 0.2 ± 0.1 | 0.2 ± 0.2 | <LOQ | <LOQ | <LOQ | <LOQ |
| PYR-1 | 0.47 ± 0.09 | 0.59 ± 0.07 | 0.36 ± 0.09 | 0.27 ± 0.07 | 0.17 ± 0.02 | 0.14 ± 0.01 |
| PYR-4,5 | <LOQ | 0.1 ± 0.2 | <LOQ | <LOQ | <LOQ | <LOQ |
| PYR-1pro | <LOQ | <LOQ | <LOQ | <LOQ | <LOQ | <LOQ |
| PYR-1et | <LOQ | <LOQ | <LOQ | <LOQ | <LOQ | <LOQ |
| PYR-1but | <LOQ | <LOQ | <LOQ | <LOQ | <LOQ | <LOQ |
| BAA | 3.6 ± 0.7 | 2 ± 2 | <LOQ | 1.2 ± 0.1 | 0.38 ± 0.03 | 0.6 ± 0.2 |
| CHR | 2.2 ± 0.5 | 1.9 ± 0.3 | 0.8 ± 0.2 | 0.65 ± 0.06 | 0.1 ± 0.04 | 0.2 ± 0.2 |
| CHR-1 | 0.15 ± 0.04 | 0.13 ± 0.04 | 0.15 ± 0.04 | <LOQ | <LOQ | <LOQ |
| CHR-6et | <LOQ | <LOQ | <LOQ | <LOQ | <LOQ | <LOQ |
| CHR-6pro | <LOQ | <LOQ | <LOQ | <LOQ | <LOQ | <LOQ |
| CHR-6but | <LOQ | <LOQ | <LOQ | <LOQ | <LOQ | <LOQ |
| BBF | 0.24 ± 0.03 | 0.24 ± 0.01 | 0.32 ± 0.04 | <LOQ | <LOQ | <LOQ |
| BKF | 0.1 ± 0 | 0.13 ± 0.05 | <LOQ | <LOQ | <LOQ | <LOQ |
| BEP | 0.5 ± 0.1 | 0.39 ± 0.06 | 0.4 ± 0.1 | 0.04 ± 0.02 | 0 ± 0.01 | 0.1 ± 0.1 |
| BAP | <LOQ | <LOQ | <LOQ | <LOQ | <LOQ | <LOQ |
| PER | 0.12 ± 0.03 | 0.07 ± 0.06 | <LOQ | <LOQ | <LOQ | <LOQ |
| IND | <LOQ | <LOQ | <LOQ | <LOQ | <LOQ | <LOQ |
| DBA | <LOQ | <LOQ | <LOQ | <LOQ | <LOQ | <LOQ |
| BGP | 0.22 ± 0.04 | 0.15 ± 0.03 | 0.6 ± 0.5 | <LOQ | <LOQ | 0.1 ± 0.1 |
| C1-BT | NA | NA | 0.16 ± 0.02 | 0.13 ± 0.03 | NA | NA |
| C2-BT | NA | NA | 0.6 ± 0.4 | <LOQ | NA | NA |
| C3-BT | NA | NA | 5 ± 0.4 | 4.6 ± 0.3 | NA | NA |
| C4-BT | NA | NA | 5.8 ± 0.3 | 5.2 ± 0.3 | NA | NA |
| C1-NAP | NA | NA | 26 ± 1 | 13 ± 1 | NA | NA |
| C2-NAP | NA | NA | 110 ± 1 | 62 ± 5 | NA | NA |
| C3-NAP | NA | NA | 163 ± 20 | 117 ± 12 | NA | NA |
| C4-NAP | NA | NA | 108 ± 15 | 80 ± 5 | NA | NA |
| C1-FLU | NA | NA | 12 ± 2 | 10.2 ± 0.8 | NA | NA |
| C2-FLU | NA | NA | 19 ± 4 | 15 ± 1 | NA | NA |
| C3-FLU | NA | NA | 18 ± 2 | 14.6 ± 0.4 | NA | NA |
| C1-DBT | NA | NA | 7 ± 1 | 4.9 ± 0.3 | NA | NA |
| C2-DBT | NA | NA | 16 ± 4 | 8.6 ± 0.7 | NA | NA |
| C3-DBT | NA | NA | 12 ± 4 | 4.5 ± 0.6 | NA | NA |
| C4-DBT | NA | NA | 14 ± 5 | 5.1 ± 0.5 | NA | NA |
| C1-PHE | NA | NA | 18 ± 3 | 11.5 ± 0.9 | NA | NA |
| C2-PHE | NA | NA | 25 ± 9 | 13 ± 2 | NA | NA |
| C3-PHE | NA | NA | 19 ± 6 | 13 ± 1 | NA | NA |
| C4-PHE | NA | NA | 24 ± 4 | 26 ± 2 | NA | NA |
| C1-PYR | NA | NA | 10.8 ± 0.9 | 6.7 ± 0.6 | NA | NA |
| C2-PYR | NA | NA | 9 ± 3 | 4 ± 0.6 | NA | NA |
| C3-PYR | NA | NA | 14 ± 6 | 0.4 ± 0.3 | NA | NA |
| C1-CHR | NA | NA | 2.3 ± 0.9 | 0.7 ± 0.1 | NA | NA |
| C2-CHR | NA | NA | 2 ± 1 | 1.8 ± 0.1 | NA | NA |
| C3-CHR | NA | NA | 9 ± 6 | <LOQ | NA | NA |
| C4-CHR | NA | NA | 19 ± 14 | <LOQ | NA | NA |
| ΣPAH | 529 ± 51 | 448 ± 15 | 257 ± 18 | 173 ± 12 | 18 ± 3 | 9 ± 2 |
| tPAH |  |  | 716 ± 80 | 452 ± 26 |  |  |
