# Supplementary material for: Correction: Oil droplet fouling and differential toxicokinetics of polycyclic aromatic hydrocarbons in embryos of Atlantic haddock and cod
Source: PLoS One. 2026 Feb 23;21(2):e0343487. doi: 10.1371/journal.pone.0343487 (PMC12928428; doi:10.1371/journal.pone.0343487)
Supplement: S5 Table — (DOCX) [file pone.0343487.s001.docx]

**S5 Table. Characterization of cardiac function and morphology at 2 dph (cod) and 3 dph (haddock).**

|  | Dose (μg/L tPAH) | AFS^1^ | VFS^1^ | SV^1^ % | Oedema mm^2^ | Length (μm) |
| --- | --- | --- | --- | --- | --- | --- |
| Cod | Control | 18 ± 4^a,b^ | 16 ± 6^a^ | 0 | 5 ± 5^a,b^ | 4652 ± 193^a^ |
|  | 0,15 | 17 ± 5^a^ | 17 ± 7^a,b^ | 0 | 7 ± 7^a,b^ | 4649 ± 192^a^ |
|  | 0,29 | 20 ± 4^a,b^ | 18 ± 5^a,b^ | 0 | 4 ± 2^a^ | 4480 ± 143^b^ |
|  | 2,8 | 19 ± 6^a,b^ | 20 ± 6^a,b^ | 0 | 8 ± 4^b,c^ | 4385 ± 191^b,c^ |
|  | 3,6 | 20 ± 5^a,b^ | 22 ± 5^b^ | 0 | 7 ± 3^a,b^ | 4265 ± 205^c^ |
|  | 9,1 | 21 ± 6^b^ | 18 ± 7^a,b^ | 3 | 12 ± 10^c,d^ | 4110 ± 201^d^ |
| Haddock 1 | Control | 19 ± 5^a^ | 19 ± 5^a^ | 0 | 4 ± 3^a^ | 4486 ± 99 ^a^ |
|  | 0,09 | 22 ± 5^b^ | 22 ± 5^b^ | 2 | 5 ± 3^a,b^ | 4363 ± 567^a^ |
|  | 0,21 | 22 ± 4^b^ | 22 ± 4^b^ | 2 | 8 ± 8^b^ | 4489 ± 222^a^ |
|  | 8,6 | 11 ± 6^c^ | 11 ± 6^c^ | 97* | 27 ± 11^c^ | 3211 ± 233^b^ |
| Haddock 2 | Control | 20 ± 8^a^ | 19 ± 5^a^ | 0 | 3 ± 3^a^ | 4564 ± 117^a^ |
|  | 0,10 | 16 ± 5^a^ | 20 ± 5^a^ | 0 | 8 ± 4^a^ | 4617 ± 151^a^ |
|  | 0,76 | 18 ± 8^a^ | 16 ± 8^a,b^ | 5 | 20 ± 8^b^ | 4191 ± 266^b^ |
|  | 2,7 | 19 ± 6^a^ | 8 ± 9^b^ | 35 | 35 ± 13^c^ | 3520 ± 410^c^ |
|  | 3,5 | 20 ± 9^a^ | 11 ± 10^b^ | 35 | 18 ± 7^b^ | 3870 ± 580^d^ |
|  | WSF (1.6 μg/L) | 24 ± 21^a^ | 16 ± 6^a,b^ | 0 | 18 ± 6^b^ | 4266 ± 180^b^ |

*very underdeveloped ventricle treated as silent ventricle

^1^VFS, ventriclular fractional shortening; AFS, atrial fractional shortening; SV, silent ventricle; EP, ethmoid plate; N, number of animals

^a–d^Letters indicate significant differences between groups (p=<0.05) (groups with same letters are not significantly different from each other).
